# Neuronal NR4A1 deficiency drives complement-coordinated synaptic stripping by microglia in a mouse model of lupus

**DOI:** 10.1038/s41392-021-00867-y

**Published:** 2022-02-18

**Authors:** Xiaojuan Han, Tianshu Xu, Congzhu Ding, Dandan Wang, Genhong Yao, Hongwei Chen, Qijun Fang, Gang Hu, Lingyun Sun

**Affiliations:** 1 Department of Rheumatology and Immunology, Department of Traditional Chinese Medicine, Nanjing Drum Tower Hospital Clinical College of Traditional Chinese and Western Medicine, Nanjing University of Chinese Medicine, Nanjing Drum Tower Hospital, the Affiliated Hospital of Nanjing University Medical School, Nanjing, China; 2 Department of Traditional Chinese Medicine, Nanjing Drum Tower Hospital, the Affiliated Hospital of Nanjing University Medical School, Nanjing, China; 3 Department of Rheumatology and Immunology, Nanjing Drum Tower Hospital, the Affiliated Hospital of Nanjing University Medical School, Nanjing, China; 4 Department of Pharmacology, Nanjing University of Chinese Medicine, Nanjing, Jiangsu China

**Keywords:** Cellular neuroscience, Neuroimmunology

## Abstract

Neuropsychiatric lupus (NPSLE) is a frequent manifestation of systemic lupus erythematosus (SLE) that occurs in 40–90% of SLE patients; however, the underlying mechanisms remain elusive, causing a severe lack of therapeutic targets for this condition. Here, we show that complement-coordinated elimination of synapses participated in NPSLE in MRL/lpr mice, a lupus-prone murine model. We demonstrated that lupus mice developed increased anxiety-like behaviors and persistent phagocytic microglial reactivation before overt peripheral lupus pathology. In the lupus brain, C1q was increased and localized at synaptic terminals, causing the apposition of phagocytic microglia and ensuing synaptic engulfment. We further determined that neuronal Nr4a1 signaling was essential for attracting C1q synaptic deposition and subsequent microglia-mediated synaptic elimination. Minocycline-mediated deactivation of microglia, antibody blockade of C1q, or neuronal restoration of *Nr4a1* protected lupus mice from synapse loss and NP manifestations. Our findings revealed an active role of neurons in coordinating microglia-mediated synaptic loss and highlighted neuronal *Nr4a1* and C1q as critical components amenable to therapeutic intervention in NPSLE.

## Introduction

Systemic lupus erythematosus (SLE) is an incurable autoimmune disease characterized by abnormal immune responses that attack normal tissues such as the kidney, skin, lung, and brain. Neuropsychiatric (NP) manifestations occur in 40–90% of patients, with symptoms ranging from anxiety, depression and cognitive impairment to psychosis, which are collectively referred to as neuropsychiatric lupus (NPSLE) or central nervous system (CNS) lupus and remain a major cause of mortality^1,2^ in SLE population. Certain autoantibodies, such as anti-NMDAR antibodies (which bind both DNA and the N-methyl-d-aspartate receptor^3^) and anti-phospholipid antibodies,^4^ have been reported as mediators of NPSLE. However, only when a breach in blood-brain barrier (BBB) integrity occurs, mostly due to secondary vasculitis or neuroinflammation, can these antibodies traverse the BBB to enter the hippocampus and other parts of the brain,^5^ and cause excitotoxic neuronal death.^6^ However, BBB compromise does not occur in early SLE cases, and even when barrier integrity is breached, some individuals do not exhibit brain pathology. Moreover, many studies have indicated that the presence of these antibodies in serum is not associated with active diffuse NPSLE,^7,8^ and the removal of B cells and antibodies in chimeric models could not alleviate brain damage in lupus mice,^9^ suggesting that these autoantibodies may not be the primary mediators and are not directly related to brain disease in lupus.

Notably, NPSLE is typically present upon lupus diagnosis or within the first year in patients,^10,11^ and emotional abnormalities are even detected before gross serological pathology in mice,^12,13^ suggesting that primary CNS factors rather than peripheral autoimmunity contributors are involved. However, the etiology and underlying mechanisms of early brain injury remain largely undiscovered.

Here, we investigate the hypothesis that NP symptoms in SLE are caused by increased elimination of synaptic terminals, which is central to the neurodegenerative process and has been observed in Alzheimer’s disease (AD) model mice^14^ and during aging.^15^ In diseased brains, activated microglia can engulf synaptic terminals through complement C4-dependent^16^ or C1q-C3 activation mechanisms,^17^ with C1q involvement particularly highlighted.^18^ Genetic or pharmacologic blockade of the complement pathway can prevent synaptic stripping and alleviate multiple age- and inflammation-related disorders.^18,19^ Despite mounting evidence that C1q assists in microglial synapse stripping, the major source of C1q is not clear, nor is how C1q cooperates with microglia to orchestrate synaptic pruning.

In addition to microglial phagocytosis, neuronal-autonomous disruption of synaptic structures or activity also contributes to synapse changes. Hyperexcitatory electrical activity results in a decreased number of dendritic spines in the diseased brain,^20^ and overexpression of the major postsynaptic density (PSD) scaffolding protein PSD-95 increases the spine size and density.^21^ However, no association between neuronal intrinsic disruption and microglia-mediated synaptic loss has been reported. It has been proposed that neurons can actively release paracrine signals to modulate phagocytic activity.^22^ One may thus hypothesize that neuron-driven complement coordinates microglia to execute synaptic degeneration, which remains to be tested experimentally. Here, we utilized the lupus-prone (MRL/MpJ-Faslpr) murine model to investigate the mediators of dendritic spines loss and to address the crosstalk between microglia, complement C1q, and their target neurons. We observed that both phagocytic microglia and C1q are necessary and highlighted that neuronal intrinsic Nr4a1 defects play an active role in orchestrating synaptic engulfment by the microglia-C1q axis. These data offer novel evidence for therapeutic intervention in SLE-driven neuropsychosis and related disorders.

## Results

### MRL/lpr mice develop behavioral deficits and microglial reactivation before the appearance of overt peripheral SLE lesions

To investigate CNS disease in lupus, we monitored brain and systemic disease development in wild-type control (MRL/mpj) and lupus-prone MRL/MpJ-Fas^lpr^ (hereafter termed MRL/lpr) mice. MRL/lpr are a Fas (CD95) gene-insertion model with defective lymphocyte apoptosis, massive lymphoproliferation, impaired autoreactive B-cell regulation, and high autoantibody titers.^23^ MRL/lpr mice develop NP manifestations early and are extensively used in lupus-related NP studies.^13^ We found that serum anti-dsDNA antibody (Ab, the typical serologic indicator of lupus) was evident by 8 weeks in MRL/lpr mice, with lupus nephritis onset at 18–20 weeks (Supplementary Fig. 1a). At 6 weeks of age, anti-dsDNA Ab titers were similar between MRL/lpr and MRL/mpj mice (Supplementary Fig. 1b), suggesting that no SLE serologic lesion was present in MRL/lpr mice at 6 weeks of age and that the disease onset time was approximately 8 weeks of age, as reported.^12^

**Fig. 1:**
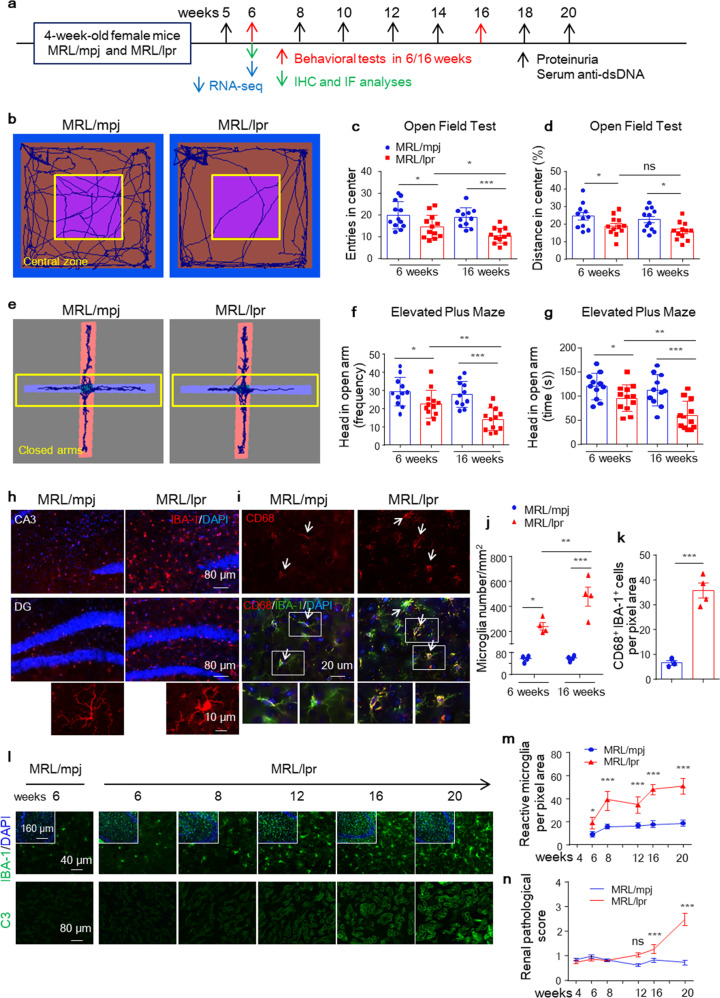
MRL/lpr mice develop anxiety-like phenotypes and reactive microglia before overt peripheral lesions. **a** Schematic of the experiment examining CNS lupus development in SLE mice. **b**–**g** Behavioral performance of 6-week-old and 16-week-old female MRL/lpr mice (red) and matched littermate controls (blue) in the open field test (OFT, **b**–**d**) and elevated plus-maze (EPM, **e**–**g**) test. *n* = 11-12 mice per group, Student’s unpaired *t*-test. **h** and **i** Representative IBA-1 immunofluorescence images of hippocampal sections (**h**). Scale bars, 80 μm and 10 μm under magnification. Immunostaining of CD68, a microglial/macrophage lysosomal activation marker, and IBA-1 in hippocampal sections from MRL/mpj and MRL/lpr mice (**i**). Scale bars, 20 μm. Arrows indicate reactive microglia. **j** and **k** Average microglial density in CA3 areas in control (blue) and MRL/lpr mice (red) (cells/mm^2^; *n* = 3-4 mice per group) (**j**). Quantitation of CD68^+^ microglia in hippocampal sections from MRL/mpj and MRL/lpr mice (*n* = 3–4 mice per group, with an average of 4-5 slices per mouse). **l**–**n** Representative image of IBA-1 in CA3 and complement C3 in kidneys from MRL/mpj and MRL/lpr mice at the indicated times; scale bars as indicated. Dot plots depicting the average number of IBA-1^+^ cells per mm^2^ (**m**) and renal pathological score (**n**) quantified in control (blue) and MRL/lpr mice (red). *n* = 5 mice per group. The data are the mean ± SEM. *P < 0.05; **P < 0.01; ***P < 0.001; and ns, not significant according to Student’s unpaired *t*-test. See also Supplementary Figs. 1 and 2

In most patients, CNS lupus typically presents at an early phase during SLE.^1,10^ To determine whether brain injury is a preexisting systemic pathology, we conducted a battery of behavioral tests on lupus mice at 6 weeks of age (when the mice had not yet developed peripheral pathology) and 16 weeks of age (when the mice began developing mild lupus nephritis) (Supplementary Fig. 1a, b). We assessed the anxiety-like behavior of MRL/lpr mice in open field test (OFT), a widely used indicator of anxiety-like behavior by evaluating the tendency of mice to remain close to the walls and avoid open spaces (central zone),^24^ and elevated plus maze (EPM) test. In the OFT, 6-week-old MRL/lpr mice were less inclined to explore the central area of the OFT chamber than the peripheral zone (Fig. 1b, c), and likewise, the distance traveled in the center (%) by MRL/lpr mice was significantly decreased compared with that by age-paired control mice (Fig. 1b, d). In the EPM test, MRL/lpr mice showed decreased entries (Fig. 1e, f) and time spent (Fig. 1e, g) in the open arms of the EPM compared with MRL/mpj animals. These anxiety-like phenotypes in MRL/lpr mice were further aggravated at 16 weeks of age (Fig. 1b–g). In the novelty Y maze, MRL/lpr mice behaved similarly to MRL/mpj mice at 6 weeks of age but showed reduced cognitive performance at 16 weeks of age (Supplementary Fig. 1c, d). In the tail suspension test (TST) and forced swim test (FST), which measure depression-like behaviors, MRL/lpr mice showed increased immobility time in the TST but not in the FST at 6 weeks of age (Supplementary Fig. 1e, f). No motor or coordination defects were found in MRL/lpr mice at either 6 or 16 weeks of age (Supplementary Fig. 1g–i). Together, these data suggest that anxiety-like behaviors are major NP changes that occur early in lupus mice. We used this phenomenon as an indicator of NP manifestation in subsequent studies.

TUNEL and Nissl’s staining did not detect ongoing apoptosis before 12 weeks of age (Supplementary Fig. 2a) or loss of Nissl’s^+^ neurons within the circuitry of the prefrontal cortex and hippocampus that mediate emotion and learning in MRL/lpr mice (Supplementary Fig. 2b). These findings suggest that apoptotic neuronal death is unlikely to be the cause of early behavioral abnormalities in SLE. Although peripheral inflammatory cells have also been reported as participants,^25^ the MRL/lpr strain showed no cellular infiltration in the brain (Supplementary Fig. 2c), indicating that rather than infiltrating immune cells, CNS-resident nonneuronal cells may be affected. Microglia, resident macrophages in the brain, respond to local inflammation or CNS damage by increasing phagocytic activity and inflammatory cytokine production.^26^ To determine whether microglial activation participates in NPSLE development, we assessed the state and proliferation of microglia via cell morphology and density in lupus brains during the early (6–8 weeks) and active (16–20 weeks) stages of SLE. As noted, in addition to increased IBA-1^+^ cell numbers (Fig. 1j and Supplementary Fig. 1k), reactive microglia in MRL/lpr mice displayed ameboid morphology, as evidenced by increased cellular soma and decreased branching complexity of cytoplasmic processes (Fig. 1h and Supplementary Fig. 1j, l). Additionally, coimmunostaining of IBA-1 with CD68, a lysosomal-localized indicator of microglial phagocytic activity,^27^ confirmed the reactivation of microglia (Fig. 1i, k and Supplementary Fig. 1j). MRL/lpr mice developed significantly increased reactive microglia in the cortex and hippocampus at 6 weeks compared with MRL/mpj mice, which was further exacerbated at 16 weeks (Fig. 1j and Supplementary Fig. 1l), with similar microglial densities in the cerebellum and midbrain (Supplementary Fig. 1k). Microglial activation in MRL/lpr mice was also confirmed by immunostaining for TEME119, another microglia-specific marker^28^ (Supplementary Fig. 1m, n).

Furthermore, relative to the observed progressive increase in IBA-1^+^ microglial intensity in the hippocampus beginning at the 6th postnatal week in MRL/lpr mice compared with age-paired MRL/mpj mice (Fig. 1l, m), complement C3 deposition in the kidneys of MRL/lpr mice was significantly increased at 16 weeks (Fig. 1l, n). Thus, these data together indicate that NP abnormalities occur in the early stage of SLE prior to peripheral pathology and are closely related to the specific activation of microglia in the brain.

### Transcriptional profiling reveals molecular changes in the hippocampus of MRL/lpr mice

**Fig. 2:**
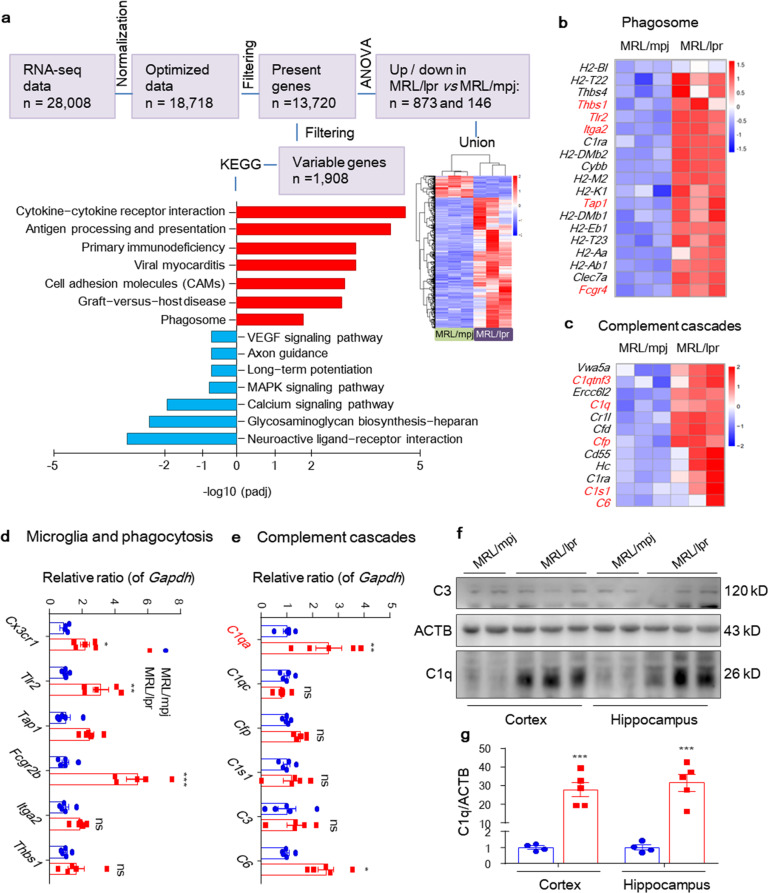
Transcriptional profile revealing increased phagocytosis and complement signal activation in the MRL/lpr mouse brain. **a** Workflow of RNA-seq data analysis of the hippocampus in MRL/lpr and MRL/mpj mice, with a heat map of differentially expressed genes. The bar chart displays 1908 variable genes, which are some of the corresponding KEGG terms and have higher expression in the MRL/mpj- or MRL/lpr- hippocampus (bottom). KEGG, Kyoto Encyclopedia of Genes and Genomes. **b** and **c** Heat maps showing the relative expression of significantly altered genes (see “Methods”) generated from the hippocampal RNA-seq of MRL/mpj vs. MRL/lpr mice at 6 weeks of age. Each column represents an individual mouse. **d** and **e** Validation of select genes and pathways in a unique set of mice by qPCR. *n* = 5–6 mice per group. **f** and **g** Immunoblotting validation of C3 and C1q in the cortex and hippocampus of 6-week-old MRL/mpj (*n* = 4) and MRL/lpr (*n* = 5) mice.The data are the mean ± SEM. *P < 0.05; **P < 0.01; ***P < 0.001; and ns, not significant according to one-way ANOVA with Tukey’s post hoc test in (**d**), (**e**), (**g**). See also Supplementary Figs. 3 and 4

To identify changes in the brain as NP manifestations develop, we performed whole-transcriptome gene expression analysis of the hippocampus in MRL/lpr and MRL/mpj mice at 6 weeks (Fig. 1a, schematic). The transcriptome assay enabled the detection of the differential expression of 1908 transcripts (cutoff of 1.5-fold change; adjusted *P* < 0.05; Fig. 2a and Supplementary Fig. 3a) and further filtering of 1019 transcripts (873 upregulated, 146 downregulated in MRL/lpr; cutoff of 1.5-log_2_fold change; adjusted *P* < 0.05). Enriched reactome pathway analysis showed that MRL/lpr mice exhibited increased SLE signaling and reduced neuronal system targets in the brain (Supplementary Fig. 3b, c), which was consistent with clinical biomarkers related to NPSLE. In addition, KEGG pathway analysis revealed signatures of cytokine-cytokine receptor interaction, antigen processing and presentation, activation of innate immune responses and microglial proteins involved in protein digestion, as well as of complement and coagulation cascades. In detail, MRL/lpr mice showed enrichment of immune-activated pathways, including classes of microglial-mediated phagocytosis (KEGG terms ‘Phagosome’ and ‘Protein digestion and absorption’, Fig. 2b and Supplementary Fig. 3d) and the classical complement pathway (KEGG term ‘the complement and coagulation cascades’, Fig. 2c). In the above category, we identified genes associated with microglial-mediated phagocytosis (*Cx3cr1*, which encodes CX3CR1; *Fcgr2b*, which encodes FCGR2B; *Thbs1*, which encodes Thrombospondin1; *Itga2*, which encodes Integrin α2; *Tlr2*, which encodes TLR2; and *Tap1*, which encodes TAP1) and the classical complement pathway (*C1qa*, which encodes C1QA; *C6*, which encodes C6; *C1s*, which encodes C1S; and *Cfp*, which encodes properdin), which were validated by quantitative PCR (Fig. 2d, e). Microglial reactive phagocytosis was further confirmed by dramatic induction of the immunofluorescence signal for CD68 in IBA-1^+^ cells in the hippocampus of MRL/lpr mice (Fig. 1i, k and Supplementary Fig. 1j). C1q and C3 are reportedly increased and required for synaptic pruning in murine brain development and many disorders.^17,29^ Immunoblotting confirmed the increase in C1q but not C3 in both the cortex and hippocampus of MRL/lpr mice (Fig. 2f, g). Conversely, MRL/lpr mice expressed lower percentages of genes that regulate neuroactive ligand-receptor interactions, long-term potentiation (LTP), axon guidance, and synapse maintenance (Supplementary Fig. 3e and Supplementary Table 1), suggesting that synaptic dysfunction may also be involved.

To determine whether microglial phagocytic activation is required for NPSLE development, we treated MRL/lpr mice with minocycline, a BBB-permeable phagocytic activity inhibitor that can suppress microglial activation,^22,30^ beginning at 5 weeks of age (shortly before reactive microglia were detected) (Supplementary Fig. 4a). Minocycline treatment significantly reduced both the IBA-1^+^ microglial number and phagocytic activity, as evidenced by reduced IBA-1^+^/CD68^+^ fluorescence intensity in the hippocampus (Supplementary Fig. 4b, c), without affecting GFAP^+^ astrocytes and NeuN^+^ neurons (Supplementary Fig. 4d, e). Minocycline also ameliorated anxiety-like behaviors in MRL/lpr mice in both the OFT (Supplementary Fig. 4f, g) and EPM tests (Supplementary Fig. 4h, i). To confirm that the observed minocycline effect was due to suppressed phagocyte activation rather than possible antimicrobial effects, we treated other groups of mice with the broad-spectrum antibiotic amoxicillin-clavulanate.^31^ However, amoxicillin-clavulanate treatment did not protect mice from disease (Supplementary Fig. 4j-l). Collectively, these results suggest that increased phagocytosis reactivation of microglia is a key mediator of anxiety-like behaviors in MRL/lpr mice.

### Synapse loss due to microglial engulfment accounts for early behavioral defects in MRL/lpr mice

**Fig. 3:**
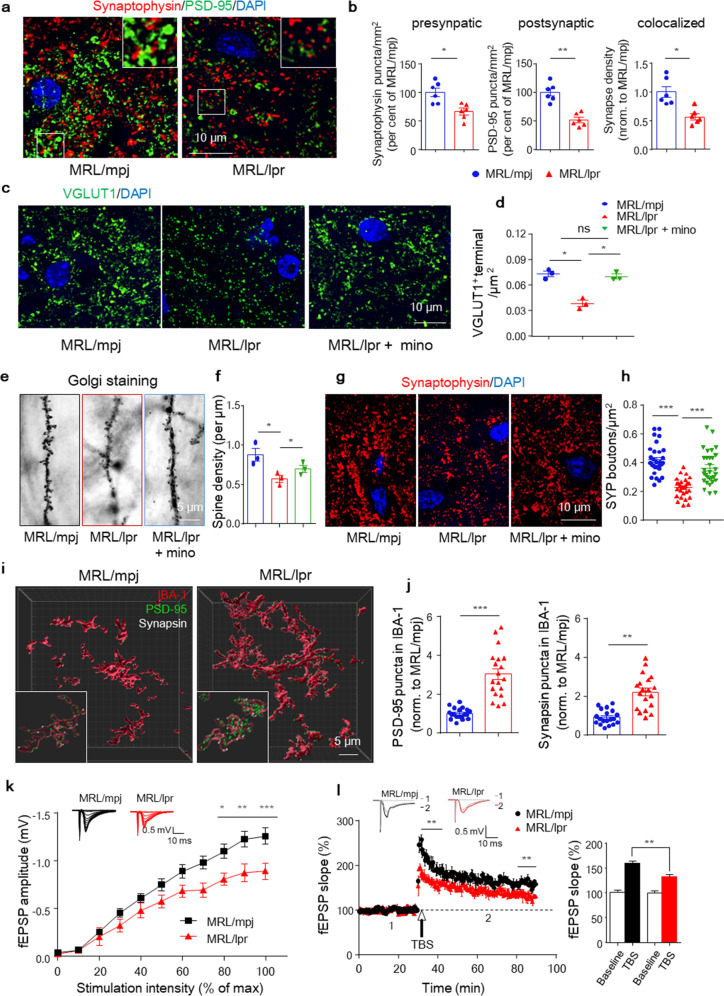
Synapse loss due to microglial engulfment accounts for early NP manifestation in MRL/lpr mice. **a** and **b** Immunostaining and quantification of presynaptic and postsynaptic puncta using the markers synaptophysin (red) and PSD-95 (green), respectively, in 6-week-old MRL/mpj and MRL/lpr hippocampal sections. Scale bar, 10 μm. The data are the means of 3 staining experiments, *n* = 6 mice per group, with an average of 3–4 slices per mouse. **c** and **d** Immunostaining and quantification of VGLUT-1 in MRL/mpj and MRL/lpr mice treated with or without minocycline. Scale bar, 10 μm. *n* = 3, with an average of 3–4 slices per mouse. **e** and **f** Representative images and quantification of Golgi-stained dendritic spines from DG granule neurons in MRL/mpj and MRL/lpr mice treated with or without minocycline. *n* = 3 mice per group. Scale bar, 5 μm. **g** and **h** Representative images and quantification of synaptophysin (SYP) bouton density in brain sections. Scale bar, 10 μm, *n* = 15–16 neurons/mouse, with 3 mice/group. **i** and **j** Representative immunofluorescence images of presynaptic terminal (synapsin, white) and postsynaptic terminal (PSD-95, green) puncta engulfed in IBA-1^+^ microglia (red) in the pyramidal layer of CA1 in the indicated mouse brain sections. Scale bar, 5 μm. A total of 18–19 cells/condition in 3–5 mice/group were analyzed. **k** and **l** Input-output curves illustrating the relationship between the magnitudes of stimulation and evoked responses for fEPSPs recorded in hippocampal slices from 6-week-old MRL/mpj and MRL/lpr mice. Insets in **k** are typical superimposed fEPSPs recorded with increasing stimulation intensity (*n* = 8 slices from three to four mice/group). (**l**, left) Time course of LTP induced by TBS in hippocampal slices. Insets are superimposed fEPSPs recorded under each condition before (1) and 60 min after the application of TBS (2). (**l**, right) Histogram showing the LTP magnitude averaged from the last 10 min of recordings from different groups (*n* = 8-11 slices from three to four mice/group. *P < 0.05, **P < 0.01, ***P < 0.001; two-way ANOVA with Bonferroni’s multiple comparison test in (**k**), repeated-measures ANOVA in (**l**). The data are the mean ± SEM. *P < 0.05; **P < 0.01; ***P < 0.001; and ns, not significant according to unpaired Student’s *t*-test in (**b**) and (**j**) and one-way ANOVA with Tukey’s post hoc test in (**d**), (**f**), and (**h**). See also Supplementary Fig. 5

Sequence analysis detected alterations in synaptic activity-related genes (Supplementary Fig. 3c, e) encouraging us to speculate that synaptic defects might be involved. For confirmation, we quantified synaptic terminals and found that the numbers of synaptic puncta within CA3 (mossy fiber terminals) were decreased at 6 weeks in MRL/lpr mice compared with MRL/mpj controls (Fig. 3a, b). The decrease was traced to a 50% reduction in the number of postsynaptic terminals, with a lesser change in the number of presynaptic terminals (Fig. 3a, b and Supplementary Fig. 5a). In MRL/lpr mice, synapse loss occurred predominantly at excitatory (VGLUT-1^+^) presynaptic terminals (Fig. 3c, d) but not at inhibitory presynaptic (VGAT1^+^) or postsynaptic (Gephyrin^+^) terminals (Supplementary Fig. 5d, e). Nevertheless, the densities of NeuN (Supplementary Fig. 5b) and TUJ1 (Supplementary Fig. 5c) remained unchanged, indicating that both neurons and axons were preserved, rendering neuroaxonal degeneration an unlikely explanation for the observed loss of synaptic input. We further quantified excitatory synaptic element numbers in dentate gyrus (DG) granule neurons of Golgi-stained tissues. Dendritic spine numbers were reduced in MRL/lpr mice (Fig. 3e, f and Supplementary Fig. 5f), consistent with the immunostaining results.

To investigate whether reactive microglial phagocytosis accounted for the observed synapse loss, we further compared the synaptic terminal changes in hippocampal sections from MRL/lpr and minocycline-treated MRL/lpr mice. As noted, minocycline restored the reductions in synaptophysin (SYP^+^) (Fig. 3g, h), especially excitatory VGLUT^+^ terminals (Fig. 3c, d), which was confirmed by Golgi staining results (Fig. 3e, f). To more directly measure synapse engulfment by microglia, we performed high-resolution imaging of synaptic markers and observed a significant increase in the number of PSD-95 and synapsin puncta presented within microglia in the hippocampal CA1 region in MRL/lpr mice (Fig. 3i, j). Ultrastructure analysis also revealed displacement of synapses encapsulated by phagocytes in the hippocampus of MRL/lpr mice (Supplementary Fig. 5g). The observed evidence collectively indicated increased synapse loss due to microglial engulfment in MRL/lpr mice. To investigate the functional correlates of disturbed synaptic terminals, we performed electrophysiological analysis in hippocampal slices from MRL/lpr and MRL/mpj mice. The input/output recordings revealed a significant reduction in basal synaptic activity (Fig. 3k) in lupus mice. LTP indicated that excitatory postsynaptic potentials (EPSPs) were also significantly reduced in MRL/lpr slices (Fig. 3l), which confirmed compromised excitatory synaptic transmission in the lupus brain.

### Increased complement C1q accumulates at synapses in MRL/lpr mice

**Fig. 4:**
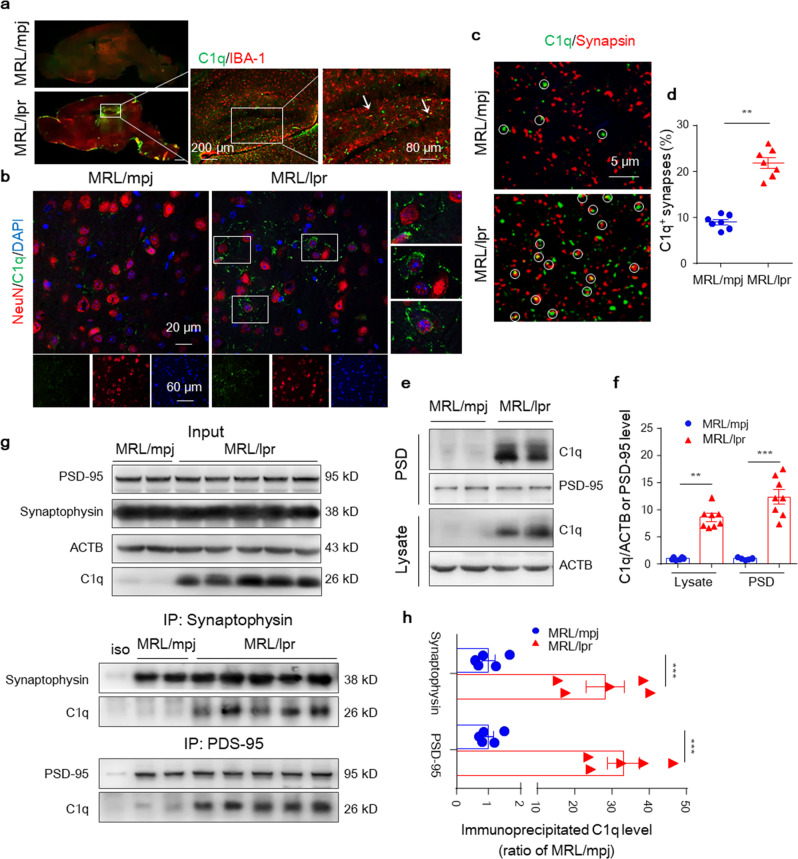
Increased C1q accumulates at synapses in the MRL/lpr hippocampus. **a** and **b** Representative images of C1q stained with IBA-1/NeuN in the dentate gyrus (DG) and CA3 (mossy fiber terminals) region of 6-week-old MRL/mpj and MRL/lpr mice. Scale bars as indicated. **c** and **d** Representative STED stacks and quantification of C1q (green) stained with synapsin (red) puncta in the CA3 region of MRL/mpj and MRL/lpr mice. Colocalized synapsin and C1q puncta are indicated by circles. Scale bar, 5 μm. **d** Graph showing the percentages of total synapsin clusters colocalized with C1q. At least 120 synapses per mouse were analyzed. *n* = 7 mice per group. **e** and **f** Representative immunoblots and quantitation of C1q in PSD fractions and total lysates of hippocampus from MRL/mpj and MRL/lpr mice. **g** and **h** Coimmunoprecipitation analysis of C1q with synaptophysin or PSD-95 antibodies in MRL/mpj or MRL/lpr hippocampal lysates. The data are the mean ± SEM. ^**^P < 0.01 and ^***^P < 0.001 according to a two-tailed Student’s *t*-test in (**d**) and one-way ANOVA with Tukey’s post hoc test in (**f**, **h**). See also Supplementary Fig. 6

Transcriptome analyses combined with immunoblotting revealed a much higher abundance of C1q protein in the lupus hippocampus. During the NPSLE process, the C1q signal was already prominent in the brain at 6 weeks (Fig. 2f), especially in the DG and CA1/3 regions, in MRL/lpr but not MRL/mpj mice (Supplementary Fig. 6a, b). Costaining experiments showed that increased C1q protein mostly located with neurons and only slightly with microglia (Fig. 4a, b) in the MRL/lpr hippocampus. In the periphery, complement C1q could function as a tag, labeling damaged cells for chemotactic phagocytosis.^32^ Coimmunostaining of C1q with a synaptic marker (synapsin) detected a much higher percentage of C1q-labeled synapses in the hippocampal CA3 region of MRL/lpr brains (Fig. 4c, d). Additionally, western blotting of C1q in purified PSD fractions versus total lysates revealed that C1q was greatly increased in the PSD of MRL/lpr hippocampi and barely detectable in MRL/mpj PSD (Fig. 4e, f). To provide further direct evidence that C1q may be involved in synaptic tagging, we performed a coimmunoprecipitation (co-IP) assay. We found that antibodies against synaptophysin and PSD-95 coimmunoprecipitated more C1q from lupus mouse hippocampal lysates (although with interanimal variability) than control mice (Fig.  4g, h). Moreover, consistent with the increased accumulation of C1q in the PSD, there was a strong positive correlation between the amount of C1q and decreased center movement in the OFT in MRL/lpr mice (Supplementary Fig. 6c). These biochemical data indicate that increased C1q accumulates at synapses in the MRL/lpr brain and is related to neurodegeneration.

Next, we wanted to examine the major source of increased C1q protein in the brain of lupus mice. Although activated microglia have been reported to be a major source of C1q in inflammatory brains, inhibition of microglial activation by minocycline did not reverse the C1q protein burden in MRL/lpr mice, although it reduced the increase in C1q mRNA level (Supplementary Fig. 6d), rendering the source of C1q a question in the lupus model. Under autoimmune conditions, many peripheral activated inflammatory cells can generate abundant C1q. By immunoblotting C1q in the brain, serum, and peripheral tissues of MRL/mpj and MRL/lpr mice, we found that C1q protein levels were much higher in the serum and kidneys than in the brain evidenced at 6 weeks of age (Supplementary Fig. 6e, f). To further clarify whether increased brain C1q may be sourced from serum, both MRL/mpj and MRL/lpr mice were intravenously injected with purified C1q (1 μg/ml). The injected C1q signal was detected in the brain within three hours (Supplementary Fig. 6h), as expected due to some permeability of the BBB to C1q, despite intact BBB integrity in MRL/lpr mice at 6 weeks (Supplementary Fig. 6g). For additional confirmation, we used a parabiosis mouse model, which has been used to examine circulatory factors transferred from one animal to another.^33^ As expected, hippocampal homogenates from MRL/mpj mice in parabiosis with MRL/lpr mice but not MRL/mpj mice exhibited increased C1q (Supplementary Fig. 6i, j). In summary, the observed significant increase in C1q protein in the MRL/lpr mouse brain may be at least partially derived from serum.

### C1q tagging at synapses contributes to microglial synaptic elimination-mediated dendritic spine loss

**Fig. 5:**
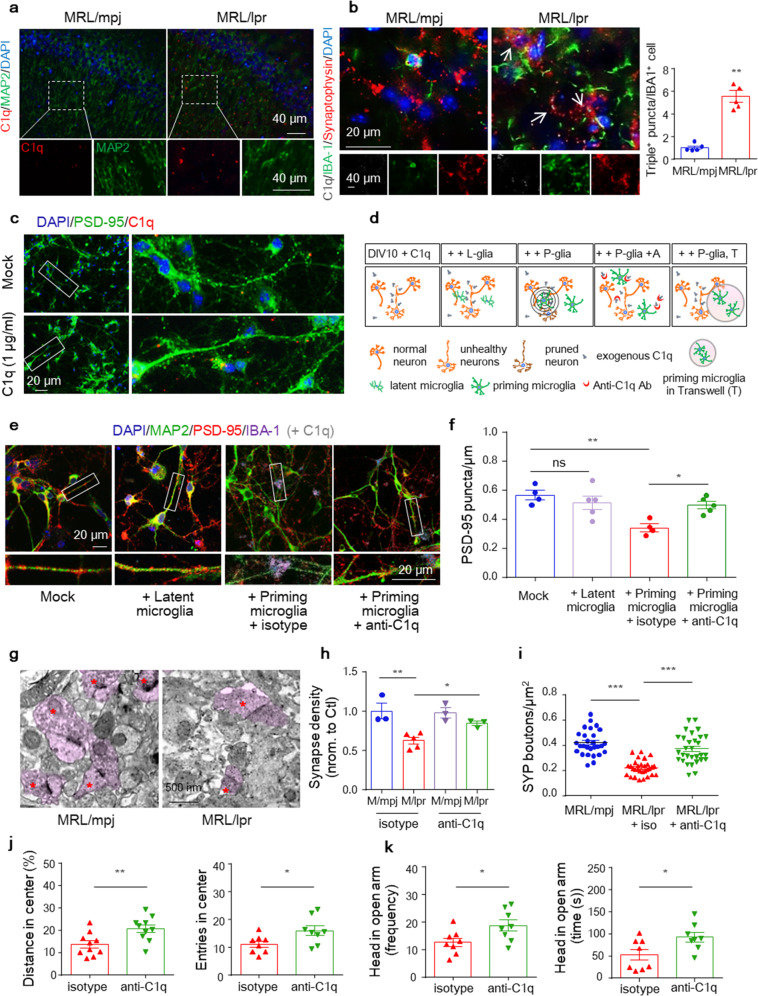
C1q tagging at synapses contributes to microglial synaptic elimination-mediated spine loss. **a** Immunostaining for C1q with the neuronal marker MAP2 in 6-week-old MRL/mpj and MRL/lpr mice. Scale bar, 40 μm. **b** Immunostaining showing colocalized synaptophysin, C1q, and IBA-1 puncta (with magnification insets and arrowheads depicting colocalization) in MRL/mpj and MRL/lpr mice. Scale bars as indicated. The data are the mean ± SEM. *n* = 5 mice per group, with an average of 3–4 slices per mouse. ^**^P < 0.01 according to Student’s *t*-test. **c** MRL/lpr mouse-derived hippocampal neurons were cultured alone or with exogenous C1q and stained for PSD-95 (green), labeled C1q (red), and DAPI (blue). Scale bar, 20 μm. (**d**) Diagrams showing microglia–neuron (or transwell) cocultures and Sholl analyses to quantify synapses around microglia. Representative images of dendritic segments (**e**) and quantification of the dendritic PSD-95 puncta density (**f**) in neuron-microglia cocultures incubated with exogenous C1q and treated with anti-C1q antibodies or the isotype control. Primary neurons of MRL/lpr mice were cocultured with latent or primed microglia and stained for IBA-1 (purple), PSD-95 (red), and MAP2 (green). Scale bar, 20 μm. The data are the means of three independent experiments (**f**). *P < 0.05; **P < 0.01; and ns, not significant by one-way ANOVA with Tukey’s multiple comparisons test. **g**–**i** Representative transmission electron microscopy (TEM) images (**g**) and quantitation of synapse density (**h**) in hippocampal sections. Scale bars, 500 nm. (*n* = 3-5 mice per group). (**i**) Density of synaptophysin (SYP^+^) puncta in the CA3 region in control and anti-C1q-injected MRL/lpr mice. Each dot represents the value for one slice. *P < 0.05; **P < 0.01; and ***P < 0.001 according to one-way ANOVA with Tukey’s post hoc test. Parameters recorded and analyzed in the OFT (**j**) and EMP test (**k**) of MRL/lpr mice post antibody injection (*n* = 8–10 mice per group). ^*^P < 0.05 and ^**^P < 0.01 according to Student’s *t*-test. All data are the mean ± SEM. See also Supplementary Fig. 7

Macrophage-mediated phagocytosis often requires antibody or complement deposition; however, no differences in the amount of IgG coating with VGLUT-1- or PSD-95-positive staining in CA3 synaptic terminals were observed in 6-week-old MRL/lpr mice compared to the controls (Supplementary Fig. 6k, l). Notably, in addition to increased PSD accumulation, C1q also colocalized in cells with neuronal morphology and MAP2-positive neurites (Fig. 5a) in the lupus brain. Moreover, C1q protein was more frequently detected in synaptophysin^+^ (SYP^+^) perisomatic synaptic boutons surrounded by or adjacent to IBA-1^+^ cellular processes in MRL/lpr mice, alongside reactivated IBA-1^+^ morphology and reduced SYP^+^ terminals (Fig. 5b).

To further examine the relationships between C1q, synaptic loss, and microglial activation, we conducted a series of in vitro studies. First, we observed that exogenous C1q colocalized with PSD-95 in neuronal cultures derived from E14 MRL/lpr mice (Fig. 5c). As a classical complement signal, C1q also functions as an initiator to mediate cell death.^34^ However, directly adding C1q to neuronal cultures failed to disturb neuronal survival, dendrites, and synapses (Supplementary Fig. 7a, f). Additionally, in cultured primary microglia, we found that unlike lipopolysaccharide (LPS, a TLR agonist and classic microglia activator) and IFNα (a reported microglia activator in lupus mice), C1q did not alter the transcription of IFN-inducible genes or NF-κB-dependent proinflammatory cytokines at the tested doses (0–50 μg/mL C1q) (Supplementary Fig. 7c) and failed to change phagocytic activity compared to IFNα and LPS (Supplementary Fig. 7d, e). These results indicate that both increased C1q and reactivated microglia may be simultaneously required for synaptic elimination.

For confirmation, we next constructed a microglia-neuron coculture system in which primary MRL/lpr mouse hippocampal neurons were cultured. One week later, the neurons were cocultured with latent or LPS prime-induced reactive microglia (3:1 neuron:microglia ratio) for another 3 days (Fig. 5d). Indeed, dendrites of neurons cultured with both exogenous C1q and primed (reactive) but not latent microglia displayed reduced synaptic density, as measured by PSD-95 clusters (Fig. 5e, f). Furthermore, when primed microglia were cocultured in a Transwell insert, non-direct-contactable coculture failed to induce C1q-microglia-axis-mediated synapse loss (Supplementary Fig. 7b, f), although the neurite length was slightly shortened. These findings highlighted the microglial activation and close microglia-neuron interactions rather than soluble neurotoxic mediators as key factors in microglial orchestration of synaptic removal.

Then, we questioned whether inhibition of C1q could prevent synapse removal by microglia. To test this hypothesis, we used a C1q-blocking antibody as previously reported,^18^ which potently blocked C1q binding to cultured neurons. We first tested whether the anti-C1q antibody could decrease microglial synapse engulfment in vitro. The C1q-blocking antibody or isotype control was added concurrently with microglia to the neuron culture (Fig. 5d), and we found that C1q-blocking antibodies, but not the isotype control, prevented the loss of PSD-95 puncta along microglia-proximal dendrites (Fig. 5e, f). To determine whether C1q-blocking antibodies could prevent synapse engulfment by microglia and rescue synapse loss in vivo, we injected C1q-blocking or isotype control antibodies into the bilateral hippocampus of 5-week-old MRL/lpr mice and analyzed the animals 3 weeks later. Remarkably, we detected significant rescue of the synapsin puncta density in the CA3 region of MRL/lpr mice injected with C1q-blocking antibody relative to control IgG by both transmission electron microscopy (EM) (Fig. 5g, h) and confocal imaging (Fig. 5i). C1q blockade also alleviated anxiety-like behaviors in MRL/lpr mice (Fig. 5j, k) without affecting the IBA-1^+^ microglia or CD68^+^ phagocyte intensity in the hippocampus (Supplementary Fig. 7g, h). Collectively, these data indicated that C1q tagging of synapses is required for synaptic elimination by microglial phagocytosis, following similar rules in the developing^29^ and adult health brains.^35^

### Neuronal *Nr4a1* defects are an endogenous signal critical to the synaptic location of C1q in MRL/lpr mice

**Fig. 6:**
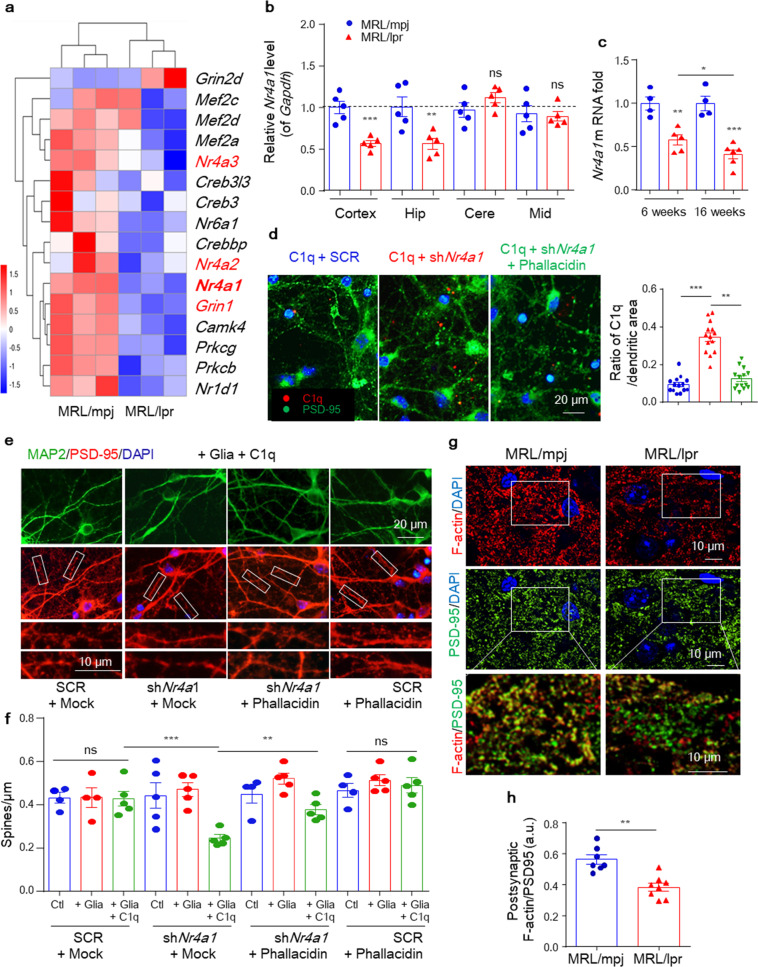
Neuronal *Nr4a1* defect is an endogenous signal critical to the synaptic location of C1q in MRL/lpr mice. **a** Heat maps showing the relative expression of significantly altered genes (involved in *Nr4a* signaling) generated from the hippocampal RNA sequencing of MRL/mpj *vs*. MRL/lpr mice at 6 weeks of age. Representative PCR and quantification of *Nr4a1* mRNA levels in brain lysates from 6-week-old MRL/mpj and MRL/lpr (*n* = 5) mice (**b**) and hippocampal lysates from both 6- and 16-week-old mice (**c**). The relative expression was normalized to the average of MRL/mpj controls. **d** Representative images of dendritic segments and quantification of C1q colocation after treatment of hippocampal neurons with the indicated shRNA or shRNA-plus-phallacidin treatment for 3 days. Scale bar, 20 μm. At least 15 neurons per culture from three independent cultures were used for the analysis. **e** and **f** Dendritic segments of neurons after treatment with control or *Nr4a1*-specific shRNA and incubation with phallacidin or the vehicle (**e**). Scale bars as indicated. The histogram shows the spine density along dendrites after treatment with shRNAs plus or minus phallacidin, as indicated (**f**). At least 11 neurons per culture from three independent neuronal cultures were used for the analysis. **g** and **h** Representative images of F-actin and PSD-95 in the hippocampal CA3 region. (**h**) Quantitation of the intensity of the F-actin signal overlapping PSD-95 puncta. Scale bar, 10 μm. Each dot represents the average for one mouse, with 7–8 mice per group. Data shown are the mean ± SEM. *P < 0.05; **P < 0.01; ***P < 0.001; and ns, not significant by one-way ANOVA with Tukey’s post hoc test in (**b**), (**f**) and unpaired Student’s *t*-test in (**c**), (**d**), (**h**). See also Supplementary Fig. 8

We next investigated the molecular mechanism that guides C1q in synapse tagging. In CNS, local apoptotic-like processes have been reported to be related with C1q label-based synaptic pruning.^36^ Other numerous mechanisms have also been indicated, including HMGB1 (high mobility group box 1),^37^ small GTPase-regulating proteins,^18^ and altered neuronal action potential.^38^ No significant changes were found in either C3 cleavage products or C5b-9 in 6-week-old lupus mice (Supplementary Fig. 3f, Supplementary Fig. 8a). Hippocampal sequencing revealed a cluster of reduced genes in the “Nr4a1 related signal transduction” pathway but not others (Fig. 6a). *Nr4a1* belongs to a family of three immediate-early genes that encode three orphan nuclear receptors (*Nr4a1*, *Nr4a2*, and *Nr4a3*).^39^ In the CNS, neuronal *Nr4a1* expression is controlled by NMDARs, CREB, and MEF2,^20,40^ which are key regulators of synaptic function. Indeed, most *Nr4a1*-controlling genes (*Grin1*, *Creb3l3*, *Mef2a, Mef2c*, and *Mef2d*) were reduced in the lupus hippocampus, as revealed by sequencing assays (Fig. 6a). We confirmed the decrease in *Nr4a1* in the prefrontal cortex and hippocampus of 6-week-old MRL/lpr mice compared with MRL/mpj controls by qPCR (Fig. 6b), and this decrease was further aggravated in 16-week-old MRL/lpr mice (Fig. 6c). Further protein–protein interaction analysis revealed that NR4A1, MEF2D, and TMOD3 act in a highly interconnected network, regulating the cellular response to endogenous stimuli and in turn controlling protein localization (Supplementary Fig. 8b), supporting the idea that NR4A1 may be related to the C1q synaptic location.

To provide more direct evidence, we performed immunofluorescence colocalization analysis. We used LV-shRNA-mediated knockdown in cultured hippocampal neurons (Supplementary Fig. 8d) and found that more exogenous C1q bound to dendrites (colocalized with PSD-95^+^ dendrites) of neurons preincubated with *shNr4a1* (Fig. 6d) than control cells (Supplementary Fig. 8e). This observation was supported by incubating neuronal cultures with AP5 (to specifically inactivate NMDARs), which reduced *Nr4a1* expression in a time-dependent manner (Supplementary Fig. 8c) and resulted in increased dendrite location of C1q (Supplementary Fig. 8e, f). Moreover, neurons incubated with sh*Nr4a1* showed a 38–46% decrease in dendritic spine density compared with the control, with no effect on axon density, when cocultured with both primed microglia and exogenous C1q (Fig. 6e, f). NR4A1 controls synapse density partially by disturbing the actin cytoskeleton,^20^ and defects in the postsynaptic actin network contribute to the elimination of dendritic spines in Tau-P301S mice.^18^ To investigate whether knockdown of *Nr4a1* encouraged C1q recognition via dysregulation of the synaptic cytoskeleton, we used the filamentous actin (F-actin)-stabilizing agent phallacidin. As shown in Fig. 6d, the increased binding of C1q to dendrites was reversed by phallacidin in cultured neurons. Functionally, in coculture analysis, phallacidin had no effect on dendritic spines in control cultures but partially restored the spine density in neurons treated with shRNAs targeting *Nr4a1* (Fig. 6e, f). We also used DIM-C-pPhCO2Me (NR4A1 antagonist) to confirm the effect of NR4A1 signaling on spine elimination. As shown, DIM-C-pPhCO2Me significantly increased the dendrite-located C1q (Supplementary Fig. 8e, f) and dependent spine loss which was alleviated by phallacidin (Supplementary Fig. 8g, h). Then, to probe state of the synaptic skeleton in vivo, we stained F-actin and the postsynaptic marker PSD-95 in the hippocampal CA3 region. Notably, we detected a significant reduction in the fluorescence intensity of the F-actin signal that colocalized with PSD-95 clusters in MRL/lpr hippocampi (Fig. 6g, h). Taken together, these observations indicate that defective neurons facilitate synaptic elimination through an NR4A1- and C1q-microglia coordinated mechanism.

**Fig. 7:**
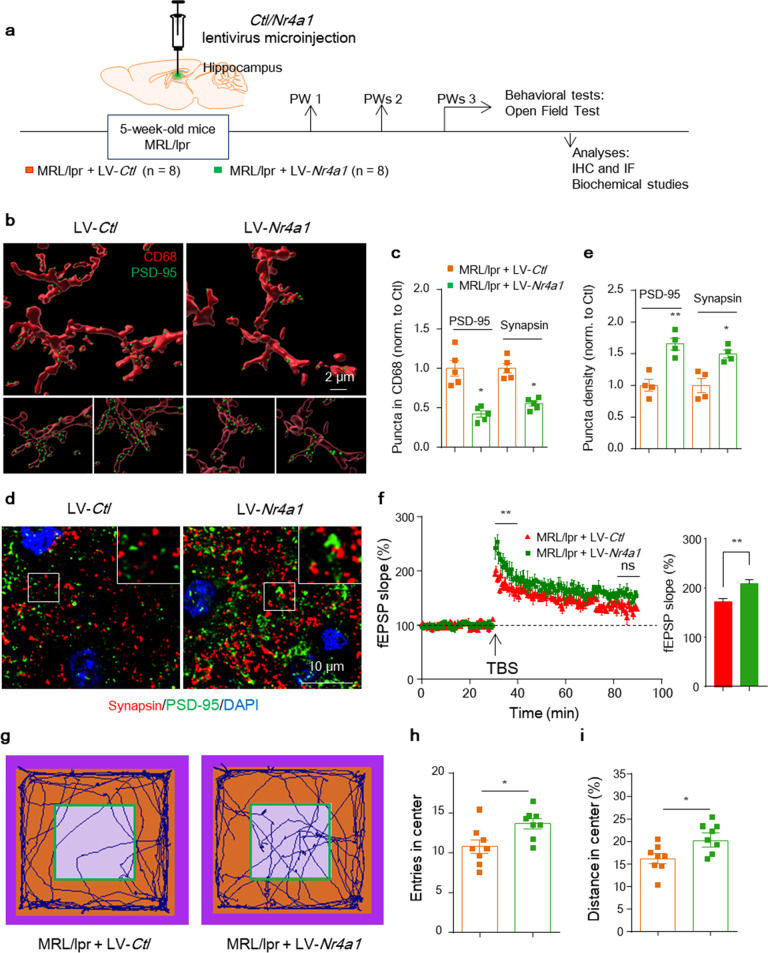
Rescuing neuronal *Nr4a1* in lupus mice protects synaptic elimination, restores hippocampal microcircuit function, and mitigates anxiety-like behaviors. **a** Timeline of the experimental procedure in the lupus-prone mouse model. **b** and **c** Representative confocal stacks of CD68^+^ lysosomes (red) and engulfed PSD-95 (green) puncta in the CA1 region of MRL/lpr mice injected with the control or *Nr4a1* lentivirus. Scale bar, 2 μm. (**c**) Quantitation of engulfed PSD-95 and synapsin puncta in CD68^+^ lysosomes normalized to LV-*Ctl*-injected mice, with 22-25 cells/group and 5 mice per group. **d** and **e** Synapsin (red) and PSD-95 (green) puncta in STED images from the CA1 region of MRL/lpr mice injected with the control or *Nr4a1* lentivirus. Scale bar, 10 μm. **e** Quantitation of the relative number of PSD-95 and synapsin clusters normalized to LV-*Ctl*-injected mice, with 12 slices/group and 4 mice per group. **f** Long-term potentiation (LTP) was induced by theta-burst stimulation over 60 min to evaluate synaptic plasticity in LV-*Ctl*- and LV-*Nr4a1*-injected MRL/lpr mice. The LTP magnitude averaged from the first and last 10 min of recordings represents the induction and maintenance of LTP, with the average from the first 10 min shown in the right histogram (*n* = 8–10 slices from three mice per group); *P < 0.05 and **P < 0.01 according to repeated-measures ANOVA. **g**–**i** Parameters recorded and analyzed in the OFT (**h**, entries into the central area and **i**, distance traveled in the central area) of MRL/lpr mice injected with LV-*Ctl* or LV-*Nr4a1* (*n* = 8 mice per group). ^*^P < 0.05 according to Student’s *t*-test in (**h**), (**i**). The data are the mean ± SEM. See also Supplementary Fig. 9

To further determine whether restoring neuronal NR4A1 expression could prevent synapse engulfment by microglia and rescue synapse loss in vivo, we injected an *Nr4a1-*overexpression lentivirus or vector control into the bilateral hippocampus of 5-week-old MRL/lpr mice and analyzed the animals 3 weeks later (Fig. 7a). The injected *Nr4a1*-GFP lentivirus was expressed in neurons throughout the hippocampus (Supplementary Fig. 9a) and resulted in a higher protein level than the control injection (Supplementary Fig. 9b) confirming the specificity and effectiveness of the *Nr4a1* lentivirus. In brains injected with LV*-Nr4a1*, the total C1q staining intensity was not affected (Supplementary Fig. 9c, d), but neuron- and synapse-located C1q was reduced in the CA1 region (Supplementary Fig. 9e, f). Additionally, a significant reduction was observed in PSD-95 or synapsin puncta within microglial lysosomes compared with LV-*Ctl* (Fig. 7b, c and Supplementary Fig. 9g, h), which alleviated the loss of synapses in hippocampal sections (Fig. 7d, e), suggesting that neuronal *Nr4a1* transcription could blunt C1q tagging and consequently synapse engulfment by microglia in MRL/lpr brains. Functionally, rescuing NRA41 expression partially restored the abnormal basal synaptic activity indicated by the I/O amplitude in MRL/lpr mice (Supplementary Fig. 9i). Compared with the control, LV-*Nr4a1*-treated mice showed enhanced induction of LTP in Schaffer collateral CA1, although the maintenance level (later population spike LTP) was not fully restored at 3 weeks after *Nr4a1* construct injection (Fig. 7f). Restoration of NR4A1 expression also improved the OFT performance of MRL/lpr mice (Fig. 7g–i). In summary, our results demonstrate that neuronal NR4A1 rescue can reduce synapse removal by the C1q-microglial axis and lead to recovery of synapse density and circuit function in vivo (Supplementary Fig. 10).

## Discussion

Our study of CNS lupus establishes that the brain is not merely a target of corrupted systemic immunity but assumes an intrinsic, active role in the early stage of the disease process. In lupus-prone MRL/lpr mice, defective neurons function as intermediaries between complement C1q and microglia. Specifically, NR4A1-deficient neurons orchestrate their own synaptic elimination by C1q-guided microglial phagocytosis, producing an NR4A1-C1q-microglial regulatory axis.

The nervous system is one of the major affected organs, and NPSLE is a major source of morbidity in the SLE population.^10^ Apart from previous considerations that NP symptoms are passive damage attributable to secondary causes such as vasculitis or brain parenchyma injury,^41^ recent evidence has indicated that NPSLE develops early, even before the diagnosis of SLE, and occurs along unique pathogenetic pathways compared with other SLE manifestations.^10^ Moreover, early restoration of systemic immunity (e.g., B cell and/or autoantibody deficiency) does not prevent NP disease in many murine SLE models.^9^ Thus, the mechanistic basis of primary NPSLE manifestations is not yet fully understood. For these reasons, we focused on brain-intrinsic pathology and the molecular mechanisms that contribute to early NP symptoms. First, via a series of combinatorial behavioral analyses, we demonstrated that anxiety-like behaviors appeared first, representing the most specific phenotype before obvious lupus serology. Then, the hippocampal RNA sequencing assay combined with stereology analysis showed that while the core architecture of the brain and total neuron numbers appeared largely intact, multiple molecular changes occurred in the hippocampus. In detail, we found that the lupus brain is enriched in a specific set of genes associated with microglial phagocytosis and the classical complement pathway but exhibits defects in genes related to synaptic function.

Neuronal apoptosis occurs in NRAb-induced NPSLE mice and patients with severe SLE in response to anti-double-stranded DNA antibodies crossreacting with neuronal NMDA receptors.^3,37^ This antibody exposure could mediate the immediate excitotoxic death of binding neurons.^6^ However, the neuronal numbers throughout the hippocampus and cortices of control and lupus mice displayed no differences. Thus, other milder, more insidious lesions may be responsible for early brain disease. Synapse loss is a hallmark central to many neurodegenerative processes, such as aging, AD, and schizophrenia.^14,42^ The unbiased identification of alterations in genes involved in synaptic function, quantified reductions in synaptic terminals within hippocampal CA1/3, and damaged neuronal circuit observed by electrophysiology provide compelling evidence that synapse loss, rather than neuron death, accounted for early-onset NP abnormalities in lupus mice.

Microglia-mediated synapse pruning plays a pivotal role in ensuring correct synaptic connectivity during normal brain development^29,43^ but also contributes to synapse pathology in CNS disorders when the synapse elimination process is reactivated.^18,27^ Indeed, hippocampal RNA sequencing identified genes associated with microglial phagocytosis. This activation was further confirmed by increased microglial numbers, altered phagocytic morphology, and increased lysosomal-localized phagocytic markers. The increased synaptic material engulfed by microglia in lupus was also visualized by EM and light microscopy, and this engulfment could be restored by microglial phagocytosis inhibition, rendering microglial engulfment accountable for synapse elimination. Inhibition of microglial phagocytic activity by minocycline can alleviate synaptic loss, which is consistent with the observed decreased in synapse engulfment by patient-derived microglia achieved by minocycline in schizophrenia models.^42^

The next question concerned why and how activated microglia target synapses, and the answer remains unclear. Antibody and complement deposition are often required for macrophage-mediated phagocytosis in the periphery. However, substantial antibody deposition was not detected early in the lupus brain, suggesting that it may not be the initiating factor. Lines of evidence suggest that C1q, the initiator of the complement pathway, is key in synapse removal by phagocytes in both developing and adult brains independently of complement activation.^15,29^ Accordingly, increased C1q was detected in lupus brains from early disease stages, and C1q was more highly enriched in purified PSD fractions than in bulk tissue. Combined with the localization of C1q at excitatory synapses adjacent to microglia, the close correlation between increased C1q and anxiety behaviors supports a model of lupus in which synapses are tagged by C1q, which guides microglia to perform engulfment, resulting in synapse loss. We further confirmed the critical contributory role of C1q in spine elimination by microglia in vitro. In the absence of C1q, neurons remained intact even when cocultured with primed active microglia. Microglial synaptic engulfment was also blunted by a C1q-blocking antibody in vivo. Thus, both in vivo and in vitro data suggested that a C1q-microglia axis targeting tagged neuronal dendrites for destruction leads to NP manifestations in lupus.

C1q, as a part of the innate immune system, is abundant in serum and many peripheral tissues, and its secretion is increased to facilitate clearance of damaged materials by phagocytes under autoimmune conditions.^44^ Thus, it is important to determine the source of C1q protein accumulation in the lupus brain. Although both neurons and microglia have been shown to secrete C1q,^45^ little direct evidence addressing this question has been presented, especially under pathological autoimmunity conditions. We detected an increased C1q mRNA level in the lupus hippocampus, representing intracerebral transcription; however, microglial deactivation by minocycline did not significantly reduce the C1q load and synaptic deposition, although it restored the increased mRNA level in the brain. Moreover, C1q protein levels displayed a much greater increase in mismatching with transcriptional upregulation, and the amount of C1q in peripheral tissue was significantly higher than that in brain tissue, suggesting that peripheral C1q may be the major source of CNS-deposited C1q in lupus mice. Both intravenously injected exogenous C1q protein and intravenously delivered homologous C1q in the parabiosis model were detected in recipient brains, further confirming serum as the major source under SLE conditions.

We then asked whether synapses are selectively tagged by C1q and, if so, how C1q recognizes tagged synapses. Generally, complement cascade products of C3b, C5b, C4b are involved in opsonization for pathogens. And evidence suggests that terminal complement effectors (C5a, MAC) have been linked to inflammatory neurological diseases.^46^ However, neither active C3 products nor C5b-9 were significantly changed in the lupus brain. These indicated that the classical complement-dependent pathway may not be essential for C1q label-based synaptic removal in the NPSLE setting, as the similar rule reported in the aging brain.^15^ A recent study reported that tau-driven proteomic changes in PSDs could provide a signal for C1q recruitment,^18^ highlighting the concept that neuronal-autonomous disruption of the synaptic composition and/or structure may participate in synapse loss. In principle, during development, intrinsic synaptic activity determines microglia-modulated pruning to preserve active synapses and remove fewer active synapses,^38^ implying that the complement can “detect” morphofunctional changes in the synapse. In these contexts, many “detectable” signals have been identified. For example, surviving DNRAb excitotoxic neurons can secrete HMGB1 to bind C1q directly.^37^ Many other signals, such as weakened neurotrophy, local reduced synaptic activity,^20,35^, and an abnormal spinal skeleton,^18,20^ are also possible.

Based on these findings, by mining sequencing data, we found a specific reduction in a set of activity-dependent genes regulating the actin cytoskeleton in the lupus hippocampus, which constitute the NMDAR/NR4A1/CREB transcriptional mechanisms. Neuronal *Nr4a1* expression is controlled by NMDARs, CREB, and MEF2, which are closely related or directly respond to neuronal activity.^20,47^ We used the NMDAR antagonist AP5 to inhibit the activity and reduce the expression of NR4A1 in cultured neurons, and we found that neurons with reduced either activity or NR4A1 expression were preferentially recognized by C1q and then engulfed by microglia in neuron-microglia coculture. This indicates that Nr4a1-microglia-mediated synapse elimination may be regulated by neuronal activity in the SLE setting, such that microglia preferentially engulf synapses of ‘weaker’ neurons, as similar rules reported in the developing brain^38^ and microglia mediate forgetting in the adult brain.^35^ Lupus mice with restored NR4A1 expression in neurons were largely protected from synapse loss and microcircuit dysfunction. Thus, our data reveal a novel mechanism that operates when microglia prune synapses. Specifically, reduced neuronal NR4A1 and disrupted synaptic skeletal homeostasis collectively function as detectable signals for C1q tagging and promote the juxtaposition of microglia to neurons, which is necessary for synaptic engulfment and displacement. In addition to neuron, a lack of *Nr4a1* in both thymus and peripheral T cells has been reported to exacerbate autoimmunity through inhibition of regulatory T cells and activation of inflammatory T cells,^48,49^ which may contribute to SLE. Collectively, although the molecular mechanisms linking NPSLE and NR4A1 remain enigmatic, our data reveal a new role of neurons in microglia-mediated synapse engulfment and identify NR4A1 as a key regulator of synaptic detection by microglia.

This study suggests that early neuropathology in lupus-prone mice is characterized by new homeostasis initiated by inherent microglial phagocytosis and C1q-dependent dendritic loss. Moreover, our studies reveal that peripheral C1q may be a major source of elevated intracerebral deposition, especially in the early stages of autoimmune encephalopathy, in contrast to C1q generated in situ by brain cells in aging or aging-related neurodegenerative diseases. Thus, in this context, a multitarget therapy with a combined systemic and brain focus may provide a more aggressive strategy than therapies focused on either location alone. Further studies are needed to identify the pathways/factors by which neuronal damage interacts with systemic immune activation. Although astrocytes did not exhibit colocalization with synaptic terminals, we cannot completely rule out their contribution to this process, as their functions in nerve support and neurotrophy differ from those of microglia.^50,51^ While NPSLE remains a heterogeneous disease with many symptoms and probably many causes, our findings suggest that early diagnosis and multiple brain-target interventions may be beneficial. BBB-permeable small-molecule drugs or drugs approved by the United States Food and Drug Administration (FDA) for other purposes^37,52^ may be attractive candidates for exploratory clinical trials.

## Materials and methods

### Mice

MRL/MpJ-Fas^lpr^ (MRL/lpr; stock # 006825) and MRL/MpJ mice (MRL/mpj; stock # 000486) were used and genotyped according to protocols provided by The Jackson Laboratory. For primary cell cultures, healthy C57BL/6J mice (female, 6–8 weeks old) were obtained from the Model Animal Research Center of Nanjing University (Nanjing, China). All animals were housed in groups of four to five mice per cage in the animal facility under standard laboratory conditions (12:12-h light cycle with free access to food and water), except when individually housed after surgery. We used a minimum of *n* = 3–6 mice per group for most analyses (except *n* = 8–15 for the behavioral analyses). The exact sample size (n) in each experiment can be found in each figure legend. All experiments were conducted in accordance with the NIH guidelines for animal research and were approved by the Animal Welfare Committee of the Affiliated Drum Tower Hospital of Nanjing University Medical School (No. 2019AE01084).

### Primary mouse neurons and microglia

Mouse neuronal cultures were prepared from the hippocampi of mouse embryos on embryonic day 13 (E13) or E14, plated on poly-D-lysine-coated coverslips at a density of 80,000/24-well dish and cultured in neurobasal medium (Invitrogen, 21103–049). Then, 50% of the medium was exchanged with fresh medium every 3 days. For primary microglial cultures, postnatal (P1-P2) pups were decapitated, the forebrain was triturated with a 5-ml serological pipette, and the homogenate was spun at 1000 × g for 5 min. The supernatant was discarded, and the pellet was resuspended in a 10-ml pipette and filtered through a 75-μm filter. Forebrains were cultured in 75-cm^2^ flasks in 15 ml of DMEM + 10% fetal bovine serum (FBS, Gibco). After 24 h, the flasks were rinsed with Hank’s balanced salt solution (Thermo Fisher Scientific), and new medium was added. The cultures were grown for an additional 10–14 days before the microglia were shaken off the astrocyte feeder layer on a rocking platform for 2 h, pelleted, resuspended in culture medium and plated.

### Microglia–neuron cocultures and quantification of synaptic density

Microglia and cortical neuron cocultures were established when the neurons reached 7 DIV using a slightly modified protocol.^18^ Primary microglia were recovered by shaking the flasks and harvested as described above. The microglia were resuspended and added to neurons (or cultured in a Transwell insert) at a 1:3 (microglia:neuron) ratio. Coculturing was continued for 3 days before fixation with 4% PFA or 1 day before harvest. To quantify the synaptic density around microglia in the cocultures, the presence of microglia, neurons and synapses was determined by immunofluorescence staining using antibodies against IBA-1 (Wako, 019-19741), MAP2 (Abcam, ab11267), and PSD-95 (Abcam, ab2723). For each coculture coverslip, 10–15 microglial cells were chosen at random for imaging. During capture, microglial cells were centered, and images were acquired by Zeiss LSM710 confocal microscopy (Carl Zeiss Jena, Germany) with a 63 × objective. Subsequent images were analyzed using the listed ModSholl Analysis Plugin Macro. PSD-95^+^ and C1q^+^ puncta were quantified manually by surveying the extracellular periphery of the cocultures.

### Pharmacological intervention

To inhibit phagocyte activation, minocycline (a tetracycline derivative extensively studied for blunting neuroinflammation and phagocyte activation) was induced. Briefly, mice received a once-daily intraperitoneal (i.p.) injection of minocycline (50 mg/kg) or PBS (control) as previously described.^22^ To investigate the potential impact of the antibiotic, mice were given daily oral administration of the broad-spectrum antibiotic amoxicillin-clavulanate (amoxicillin trihydrate: potassium clavulanate (4:1), 50 mg/ml in the drinking water) or mock treatment (nonsupplemented drinking water) as reported.^22^

### Stereotaxic antibody and *Nr4a1* lentivirus microinjection

The blocking anti-C1q antibody was produced using the variable domain sequences previously described,^18^ with isotype mouse IgG2b as the control. Five-week-old female mice were anesthetized with 3% isoflurane and placed on a stereotaxic apparatus (Stoelting) for surgery. All injections were performed with a 10-μl syringe (Hamilton Company) with a pulled-glass pipette tip glued to the end of the needle and a syringe pump (Stoelting Quintessential stereotaxic injector). The injection coordinates were 1.25 mm anterior, ±1.82 mm lateral, and 1.75 mm ventral to bregma. One microliter of anti-C1q or isotype control antibody was injected at a rate of 0.5 μl/min. For *Nr4a1* lentivirus microinjection, mice were randomly assigned and microinjected bilaterally with either the control or *Nr4a1*-GFP lentivirus (1 μl of 1 × 10^9^ viral genomes/μl, Hanbio, Shanghai, China) in the hippocampus according to the above coordinates.

### Tissue imaging and analysis

For total immunofluorescence analysis, sections were scanned by Zeiss LSM710 confocal microscopy (Carl Zeiss Co., Germany). Color thresholds in RGB space were used to identify the positive staining. The mean fluorescence intensities of regions of interest were measured with Fiji software (ImageJ). In total, 3–5 sections per mouse were collected for analysis. For microglial engulfment analysis, sections were imaged using an Olympus SpinSR spinning disk confocal microscope, reaching 32 z-stacks at 0.34-μm steps with a 100× oil objective. Six to eight microglia within the hippocampal CA1 or CA3 region per mouse were analyzed. IBA-1-positive microglia and CD68-positive lysosomes were then 3D-reconstructed with Imaris software (Bitplane) using the surface rendering function, and synapsin or PSD-95 puncta inside the IBA-1^+^ phagocytes and CD68^+^ lysosomes were visualized and quantified using the spot rendering function. A total of 6–10 microglia within the hippocampal CA3 region per mouse, with 3–5 mice per group, were analyzed. For STED superresolution imaging, hippocampal sections stained with appropriate synaptic markers and C1q were imaged using a Leica SP8 STED 3× microscope with a 100x objective. For C1q-Synapsin colocalization, four independent fields of view in the CA3 region per mouse were captured, and for each field a 3-μm stack was imaged at 100× using a 0.1 μm z-step. STED images were analyzed using ImageJ, and when the distance between detected cluster centers was shorter than 200 nm, the clusters were considered colocalized. For synapse quantification, hippocampal sections were stained for synapsin, and PSD-95 in CA1/CA3 regions were imaged with an Olympus spinning disk confocal microscope. Three independent fields of view per slice were imaged at 100× using a 3 μm z stack comprised of 1 μm z-steps. Acquired images were deconvoluted using the Huygens package, and the number of colocalized pre- and postsynaptic puncta in each field was measured using ImageJ software (NIH) based on the synaptic markers used. All imaging and analyses were performed blind to genotype. DAB-stained samples were imaged using a confocal microscope (Axiovert LSM510, Carl Zeiss Co., Germany), and immunostaining signals were quantitatively analyzed by the Optical Fractionator method with Stereo Investigator software (Stereo Investigator software; Microbrightfield) as previously described.^53^ Golgi-stained samples were imaged by confocal microscopy (FV3000 Microscope, Olympus Co., Japan).

### Electrophysiological recordings in hippocampal slices

Coronal brain slices containing the hippocampal formation were prepared as previously described.^15^ Animals were anesthetized with isoflurane, and each brain was rapidly extracted from the skull and transferred into ice-cold slicing artificial cerebrospinal fluid (ACSF) solution (mM: 125 NaCl, 2.5 KCl, 1.25, NaH_2_PO_4_, 25 NaHCO_3_, 10 d-glucose, 2.0 CaCl_2_, 1.5 MgCl_2_, saturated with 95% O_2_/5% CO_2_), where the hippocampus was isolated from the cortices. Transversal slices (380 μm thickness) were prepared with a Vibratome VT1200S (Leica, Germany) and recovered in incubation ACSF (same formula used for slicing ACSF) at 30 °C for 30 min, followed by incubation at RT for an additional 1 h and transfer to a “submerged” -type recording chamber and continuous perfusion with ACSF recording (mM: 125 NaCl, 2.5 KCl, 1.25 NaH_2_PO_4_, 25 NaHCO_3_, 10 d-glucose, 2.0 CaCl_2_, 1.0 MgCl_2_; saturated with 95% O_2_/5% CO_2_) with a peristaltic pump (BT100-2J, LongerPump, China) at 7 ml/min and 30.0 ± 0.1 °C. Recordings were acquired with an Axopatch 700B amplifier and Digidata 1440A (Molecular Devices, USA). pCLAMP 10.7 software was used for recording acquisition. For field excitatory postsynaptic potential (fEPSP) recordings, a recording pipette was placed in the middle of the stratum radiatum in CA1, 75–150 μm deep in the tissue. fEPSPs were evoked by activating Shaffer collaterals with a glass pipette (1–2 MΩ) placed in the middle of the stratum radiatum 200–400 μm away from the recording pipette. Input-output curves were generated by a series of stimuli in 0.01-mA steps. The amplitude of the fEPSPs was set at 35–40% of the maximal responses for paired-pulse facilitation recording. Paired stimuli (25-, 50-, 75-, 100-, and 200-ms intervals) were delivered, and the ratio of the amplitude and/or slope of the second fEPSP to the first fEPSP was calculated. For LTP recording, slices were stimulated with single test pulses every 30 s to elicit a stable baseline response for at least 30 min, and then LTP was induced by theta-burst stimulation (TBS, two trains of 10 bursts (5 Hz) delivered at 20-s intervals; each burst consisted of four pulses of 100 Hz). Following TBS, the stimulus frequency was returned to 30 s for 60 min. Fifty to sixty minutes after stimulation corresponded to the early phase of LTP. All recordings were performed in a blinded manner.

### Mouse behavioral testing

For all behavioral tests, mice were transferred to the test room and equilibrated for at least 3 h prior to testing, which was conducted between 9:00 and 18:00 h. Behavior was monitored through a video camera positioned in front or on top of the testing apparatuses and was recorded and later analyzed with a video tracking system (Top Scan software; Top Scan Software & Instruments, USA) by a blinded, experienced researcher. A panel of behavioral tests was used for phenotypic characterization of MRL/lpr as previously described.^13^ These included the TST and FST to assess depression-like behaviors, the novelty Y maze test to assess memory, the OFT combined with the EPM test to examine anxiety-like behaviors, and the OFT combined with the rotarod test to examine locomotion. The detailed materials and methods of these tests are provided in the Supplementary information.

### Statistical analysis

For all statistical analyses, GraphPad Prism 6 Software was used. The data were expressed as the mean ± SEM and were analyzed using one-way ANOVA, two-way ANOVA, or Student’s *t*-test. For ANOVA, Tukey’s post hoc tests were used. Correlations were measured using Pearson’s correlation coefficient, and interactive effects were analyzed by multivariate linear regression. Significance is reported at *P* < 0.05.

## Supplementary information

Supplementary Materials

Supplementary Data 1

Supplementary Data 2

## Data Availability

The RNA sequencing data supporting the findings of this study have been deposited into the NCBI GEO data repository: GSE154288 (https://www.ncbi.nlm.nih.gov/geo/info/linking.html). Other data supporting the findings of this study are available from the corresponding author upon reasonable request.
