# Correction To: Neuronal NR4A1 deficiency drives complement-coordinated synaptic stripping by microglia in a mouse model of lupus

**DOI:** 10.1038/s41392-022-01155-z

**Published:** 2022-09-17

**Authors:** Xiaojuan Han, Tianshu Xu, Congzhu Ding, Dandan Wang, Genhong Yao, Hongwei Chen, Qijun Fang, Gang Hu, Lingyun Sun

**Affiliations:** 1 Department of Rheumatology and Immunology, Department of Traditional Chinese Medicine, Nanjing Drum Tower Hospital Clinical College of Traditional Chinese and Western Medicine, Nanjing University of Chinese Medicine, Nanjing Drum Tower Hospital, the Affiliated Hospital of Nanjing University Medical School, Nanjing, China; 2 Department of Traditional Chinese Medicine, Nanjing Drum Tower Hospital, the Affiliated Hospital of Nanjing University Medical School, Nanjing, China; 3 Department of Rheumatology and Immunology, Nanjing Drum Tower Hospital, the Affiliated Hospital of Nanjing University Medical School, Nanjing, China; 4 Department of Pharmacology, Nanjing University of Chinese Medicine, Nanjing, Jiangsu China

**Keywords:** Cellular neuroscience, Neuroimmunology

Correction to: *Signal Transduction and Targeted Therapy* 10.1038/s41392-021-00867-y, published online 18 February 2022

In the process of collating the raw data, the authors noticed two inadvertent mistakes occurred in Fig. 4b and Fig. 6g (and the corresponding uncropped images in “Supplementary Data 2-Figure 6g”) that need to be corrected after online publication of the article^1^. The correct data are provided as follows. The key findings of the article are not affected by these corrections. The original article has been corrected.

1. Staining labels in Fig. 4b were mislabeled as “NeuN” (Green) and “C1q” (Red), which should be “NeuN” (Red) and “C1q” (Green) as shown below.
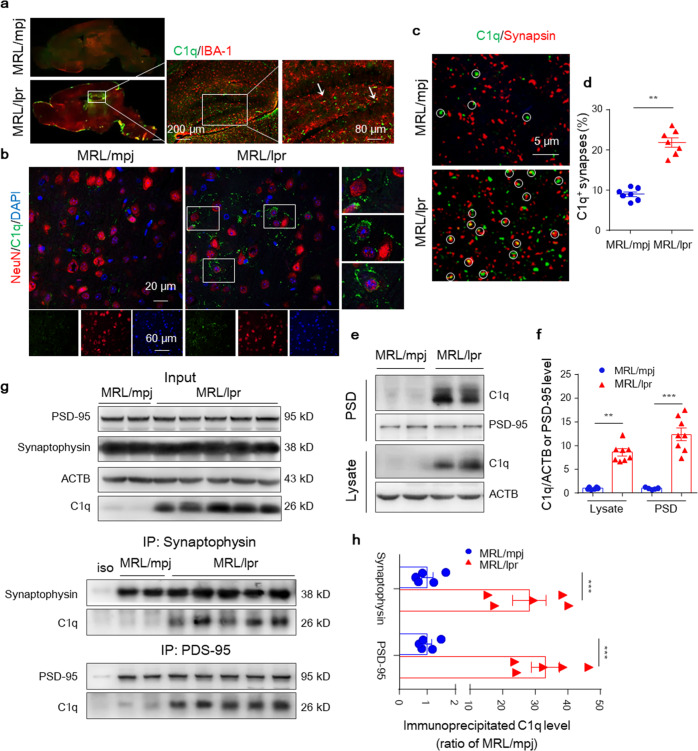
  **Fig. 4b** Representative images of C1q stained with NeuN in the CA3 region of 6-week-old MRL/mpj and MRL/lpr mice.
2. The middle and bottom panels of Fig. 6g that showed the fluorescence images of PSD-95 and F-actin/PSD-95 co-location in the mouse hippocampal sections were wrongly inserted. The correct results should be as shown below.

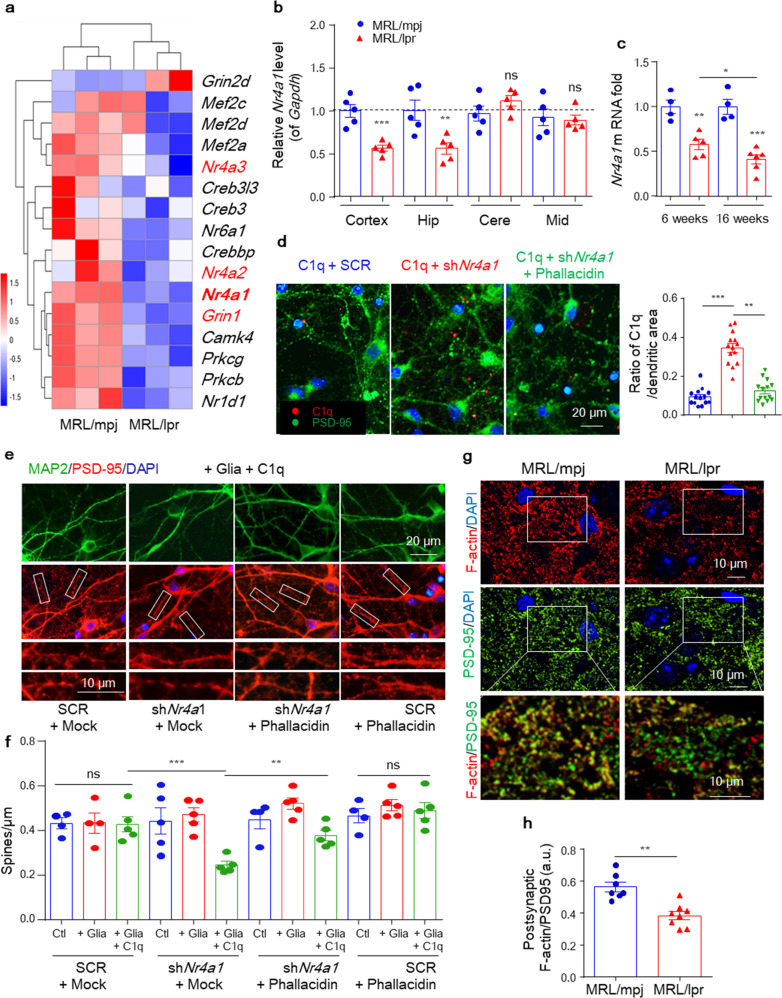


**Fig. 6g** Representative images of F-actin and PSD-95 in the hippocampal CA3 region.

## Supplementary information

Supplementary Data 2 - Updated
