# Supplementary material for: Neuronal NR4A1 deficiency drives complement-coordinated synaptic stripping by microglia in a mouse model of lupus
Source: Signal Transduct Target Ther. 2022 Feb 18;7:50. doi: 10.1038/s41392-021-00867-y (PMC8854434; doi:10.1038/s41392-021-00867-y)
Supplement: Supplementary file 1 — Supplementary Materials [file 41392_2021_867_MOESM1_ESM.docx]

Supplementary Materials for

**Neuronal NR4A1 deficiency drives complement-coordinated synaptic stripping by microglia in a mouse model of lupus**

**Running title: Neurons coordinate synaptic stripping by microglia in lupus**

Xiaojuan Han^1,2,3#^, Tianshu Xu^2#^, Congzhu Ding^1,3#^, Dandan Wang^1,3^, Genhong Yao^1,3^, Hongwei Chen^1,3^, Qijun Fang^2^, Gang Hu^4*^, and Lingyun Sun^1,3,5*^

^1^ Department of Rheumatology and Immunology, Department of Traditional Chinese Medicine, Nanjing Drum Tower Hospital Clinical College of Traditional Chinese and Western Medicine, Nanjing University of Chinese Medicine, Nanjing Drum Tower Hospital, the Affiliated Hospital of Nanjing University Medical School, Nanjing, China;

^2^ Department of Traditional Chinese Medicine, Nanjing Drum Tower Hospital, the Affiliated Hospital of Nanjing University Medical School, Nanjing, China;

^3^ Department of Rheumatology and Immunology, Nanjing Drum Tower Hospital, the Affiliated Hospital of Nanjing University Medical School, Nanjing, China;

^4^ Department of Pharmacology, Nanjing University of Chinese Medicine, Nanjing, Jiangsu, China.

^5^ Lead Contact

^#^ X.H. T.X. and C.D. contributed equally to this work

^*^ Correspondence: lingyunsun@nju.edu.cn (L.S.) and [ghu@njmu.edu.cn](mailto:ghu@njmu.edu.cn) (G.H.)

**This file includes:**

Supplementary materials and methods

Figures. S1 to S10

Tables S1 to S4**Supplementary materials and methods**

**Tissue collection and sample preparation**

Mice were anesthetized with 3% isoflurane and then transcardially perfused with ice-cold PBS. One hemisphere was dissected for isolation of the prefrontal cortex, hippocampus, cerebellum, and midbrain. A fraction of samples was used for RNA extraction, whereas other samples were homogenized in RIPA buffer (Pierce) containing phosphatase and protease inhibitors (Invitrogen) and centrifuged for 15 min at 15,000 × g, and the supernatant was used for Western blotting. For RNA-seq, a fraction of the hippocampal samples was used.

**Brain immunohistochemical and immunofluorescence staining**

Mice were deeply anesthetized and transcardially perfused with PBS, followed by the addition of 4% paraformaldehyde (PFA) in PBS for fixation, postfixation in 4% PFA overnight, and cryoprotection in 20% sucrose. Alternatively, the right hemibrain was drop-fixed for 48 h at 4°C in 4% PFA for immunohistochemistry, and the left forebrain was frozen and later homogenized. Brains were embedded in OTC and sectioned at 30 μm using a freezing microtome. Sections were preserved in cryoprotectant (50% glycerol and 50% PBS) and stored at -20°C. For H&E staining, brains were embedded in paraffin and sectioned at 15 μm. For fluorescence immunostaining, free-floating sections were rinsed in PBS followed by PBS with 0.3% Triton X-100 (PBST), blocked with blocking buffer (5% goat serum, 5% bovine serum albumin in PBST), and incubated overnight with primary antibodies. Secondary antibodies were incubated for 1-2 h at room temperature, extensively washed in PBS, and mounted using ProLong Diamond medium (Invitrogen). For bright field immunochemical staining, endogenous peroxidases were neutralized (PBS/3% H_2_O_2_), and nonspecific binding was blocked. Then, sections were stained with primary and secondary antibodies and visualized by 3’-diaminobenzidine (DAB) immunostaining. The primary and secondary antibodies used are listed in Supplementary information, Table S3. For neuron quantification, sections were stained with Nissl stain (0.05% thionine/0.08 M acetate buffer, pH 4.5). For apoptosis quantification, sections were stained with a TUNEL Kit (KGA7062, Keygen). For synapse quantification, mouse brains were prepared according to the manufacturer’s instructions, and sections were stained with an FD Rapid GolgiStain Kit (FD Neurotechnologies) as previously described^1^.

**Synaptosome fractionation**

Synaptosomes were isolated using a modified version of the protocol outlined^2^. Briefly, tissue was immersed in 10 volumes of HEPES-buffered sucrose (0.32 M, 5 mM HEPES, pH 7.4) and homogenized using a motor driven glass-Teflon homogenizer. The resulting homogenate was spun at 1200 × g to separate the nuclear fraction. A further spin at 15, 000 × g was carried out to generate crude synaptosomes. These were then layered onto a discontinuous sucrose gradient and spun at 150, 000 × g for 2 h. Purified synaptosomes were subsequently extracted and spun down.

**Tissue preparation for TEM**

For TEM, methods were adopted as previously reported^3^. In brief, mice were perfused with a 2% PFA/2.5% glutaraldehyde solution in 0.1 M phosphate buffer (pH 7.4), and coronal sections (150 μm) including the hippocampus, were cut using a vibratome and further fixed overnight. Then, tissues were processed using 2% osmium tetroxide and 4% uranyl acetate, dehydrated, and embedded in Eponate 12 resin (Ted Pella Inc., Redding, CA). Ultrathin sections were cut at a thickness of 1 µm, collected on copper grids, and imaged with FEI Tecnai G2 Spirit Bio TWIN TEM using FEI software. Fifteen distinct regions of the hippocampus were imaged per animal. Images were used to analyze the number of synapses with blinding to the genotype. A synapse was defined as an electron-dense postsynaptic density area juxtaposed to a presynaptic terminal filled with synaptic vesicles. Microglia were identified on the basis of their characteristic nuclear morphology, a single-profile endoplasmic reticulum, and electron-dark shading of the cytoplasm.

**Western blotting**

Equal amounts of protein (30 μg) were separated by electrophoresis in precast 8–12% Bis-Tris Gels (Bio-Rad) and transferred to prewetted polyvinylidene difluoride membranes. The membranes were hybridized with primary and then secondary antibodies. The membranes were hybridized with the primary and then secondary antibodies as listed in Supplementary Table S3.Then, ECL (Pierce®) was used to reveal the immunoreactive proteins, images were acquired using an Image Quant LAS 4000 mini (Uppsala, Sweden) luminescent image analyzer, and protein bands were quantified using ImageJ software (NIH). Protein levels were determined by normalizing to the level of ACTB and are presented relative to the control.

**RNA sequencing**

A total amount of 3 μg of RNA per sample was used as input material for the RNA sample preparations, with mRNA purified from total RNA using poly-T oligo-attached magnetic beads. Sequencing libraries were generated using the NEBNext® Ultra^TM^ RNA Library Prep Kit for Illumina® (NEB, USA) following the manufacturer’s recommendations (Novogene Co., LTD). Sequencing reads were aligned to the mouse reference genome mm10 (GRCm38.90) using STAR aligner (v2.5.1b) guided by the mouse GENCODE gene model release v15. HTSeq v0.6.0 was used to count the read numbers mapped to each gene. Then, the FPKM of each gene was calculated based on the length of the gene and the read count mapped to this gene. Raw count data were normalized by the voom function in the R limma package, and differential expression analysis was then performed using the DESeq2 R package (1.10.1). Differentially expressed genes (DEGs) were defined as having at least a 1.5-fold change in expression and adjusted *P* < 0.05 in comparisons of different genotypes.

**Mouse behavioral testing**

**Open field.** The mouse open field chambers were made of Plexiglas and consisted of a square base (40 × 40 × 30 cm). For each testing session, the mouse was allowed free exploration in the environment for 5 min. A computer-assisted video-tracking system (TopScan software, CleverSys Inc.) was used to record the number of times and entries into the center and peripheral zones. The total distance traveled (cm) and the mean velocity (mm/s) were used as measurements of general locomotor activity. A decrease in the distance in the center (%) or a decrease in entries in the center zone (per trial) was used as an assessment of anxiety-like behavior.

**Elevated plus maze.** The elevated plus maze consists of two open and two closed arms that extend out from a central platform. Each arm of the maze is 30 cm long and 5 cm wide. Mice were placed in the center platform of the maze, facing an open arm, and allowed to explore the apparatus for 6 min. A computer-assisted video-tracking system (TopScan software, CleverSys Inc.) was used to record the number of open and closed arm entries (all four paws in an arm) as well as the total time spent in open, closed, and center compartments. A decrease in the percent time spent in the open arms (sec) or a decrease in the percent entries into the open arms (frequency) was used as a surrogate measure of anxiety-like behavior.

**Rotarod test.** The purpose of the rotarod test is to assess the sensorimotor coordination and/or fatigue resistance of the mouse. The rotarod apparatus consists of a gritted plastic roller flanked by large plates on each end to prevent the animal from escaping. The plastic rod sits at a height of approximately 20 cm above individual electronic sensing platforms. Prior to actual testing, mice were habituated to the rotarod apparatus by placing them on the rod as it revolved at a very low rotation speed (4 r.p.m. for 5 min). Following the habituation session, mice were tested in the accelerated rotarod (4-20 r.p.m. in 3 min). The latency until the mouse fell from the rod onto the sensing platform below was recorded automatically. This test was repeated after a 30-min break for a total of 3 repeats. The maximum time on the rotarod was limited to 6 mins.

**Novelty Y maze.** Testing was conducted in a clear acrylic Y maze with three arms (one start arm and two test arms, all ~ 31 cm in length) with a removable partition to block the appropriate arm. The test consisted of a forced choice trial followed by a free-choice trial. For the forced choice trial, the start arm and one test arm were open, with access to the second test arm blocked by the partition. Individual subjects were placed in the start arm and allowed to explore the open test arm for 3 min, after which they were removed from the maze and placed in a holding cage as the maze was cleaned. The partition was removed, and the mice were then immediately placed back into the Y maze for the free-choice trial and allowed to explore both the open and test arms for 3 min. The delay between the forced choice and free-choice trials was approximately 3 min. The animal behavior was video-recorded during both trials, and the time spent in the previously accessible arm (i.e., the familiar arm; tf) and the previously blocked arm (i.e., the novel arm; tn) was determined during the free-choice trial using TopScan software. For each subject, the entries in the familiar arm (frequency) and percentage of time exploring the novel arm (%) during the free-choice trial were calculated using the formula: percentage of tn = tn / (tn + tf) × 100. A decrease in either parameter was used as a measure of anxiety-like behavior.

**Forced swim test.** This procedure is used to induce a despair-like state and to test the effects of antidepressants in mice. Mice were placed for 5 min in a glass cylinder (height, 35 cm; diameter, 17 cm) filled with water (25 ± 2°C) to a depth of 25 cm. The water depth was adjusted so that the animals had to swim or float without their hind limbs or tail touching the bottom. During testing, the duration of immobility (the time during which the subject made only small movements necessary to keep their heads above water) was scored. The mouse was immediately removed from the cylinder and excluded from the study if it failed to swim or keep its head above water. After completion of the trial, the subject was dried using a paper towel and placed back in its home cage. After every trial, the water was changed, and the cylinder was rinsed with clean water.

**Tail-suspension test.** This procedure is an alternative to the forced swim test. A depression-like state will increase the amount of time the mouse spends immobile in this test. At the beginning of a trial, the mice were suspended by the tail (taped onto a suspension hook so that the animal would hang with its tail in a straight line) above a flat surface covered with soft padded material. During testing (5-min trial), the duration of immobility was scored automatically (TopScan software).

**Parabiosis model**

The mice to be joined in parabiosis were anesthetized and shaved along the opposite lateral flanks as reported in established protocols^4^. The excess hair was wiped off with an alcohol prep pad. After further disinfection with betadine solution and 70% alcohol, identical incisions were created on the corresponding lateral aspects from the olecranon to the knee joint of each mouse. The olecranon and knee joints were each attached by a single 4-0 silk suture and tie, and the dorsal and ventral skins were sewed together with continuous 5-0 Vicryl sutures. The mice were then kept on heating pads and continuously monitored until full recovery. Buprenorphine was used for analgesic treatment by subcutaneous injection every 8-12 h for 48 h after the operation.

**Blood-brain-barrier leakage assay**

Blood-brain barrier leakage was assayed using established protocols^5^. Mice were injected intravenously with 40-kDa FITC-dextran (2 mg per 20 g mouse). After 4 h of circulation, mice were euthanized, and brains were fixed in 4% PFA. Coronal brain sections (30 μm) were stained with anti-CD31 (1:500, Abcam) to mark blood vessels and were imaged by confocal microscopy. Three fields of view from two brain sections per animal were quantified using ImageJ. Leakage was measured as a decrease in colocalization of FITC-dextran with CD31.

**Assessment of lupus**

Mice were monitored for the development of proteinuria and autoantibody titers during the course of the experiment. Urinary protein excretion was measured by a Bradford protein detection kit (KGA801-804, Keygen Biotech). Serum IgG anti-double stranded (ds) DNA antibody titers were measured by ELISA (FUJIFILM, Cat # 631-02699) according to the manufacturer's instructions, and serum samples were diluted 1:250 in buffer solution before being added to the plates for incubation.

**References**

1. Stephan, A.H. et al. A Dramatic Increase of C1q Protein in the CNS during Normal Aging. *J Neurosci* **33**, 13460-13474 (2013).

2. Györffy, B.A. et al. Local apoptotic-like mechanisms underlie complement-mediated synaptic pruning. *Proc Natl Acad Sci USA* **115**, 6303-6308 (2018).

3. Vasek, M.J. et al. A complement–microglial axis drives synapse loss during virus-induced memory impairment. *Nature* **534**, 538-543 (2016).

4. Katsimpardi, L. et al. Vascular and Neurogenic Rejuvenation of the Aging Mouse Brain by Young Systemic Factors. *Science* **344**, 630-634 (2014).

5. Armulik, A. et al. Pericytes regulate the blood-brain barrier. *Nature* **468**, 557-61 (2010).

**Figure. S1.
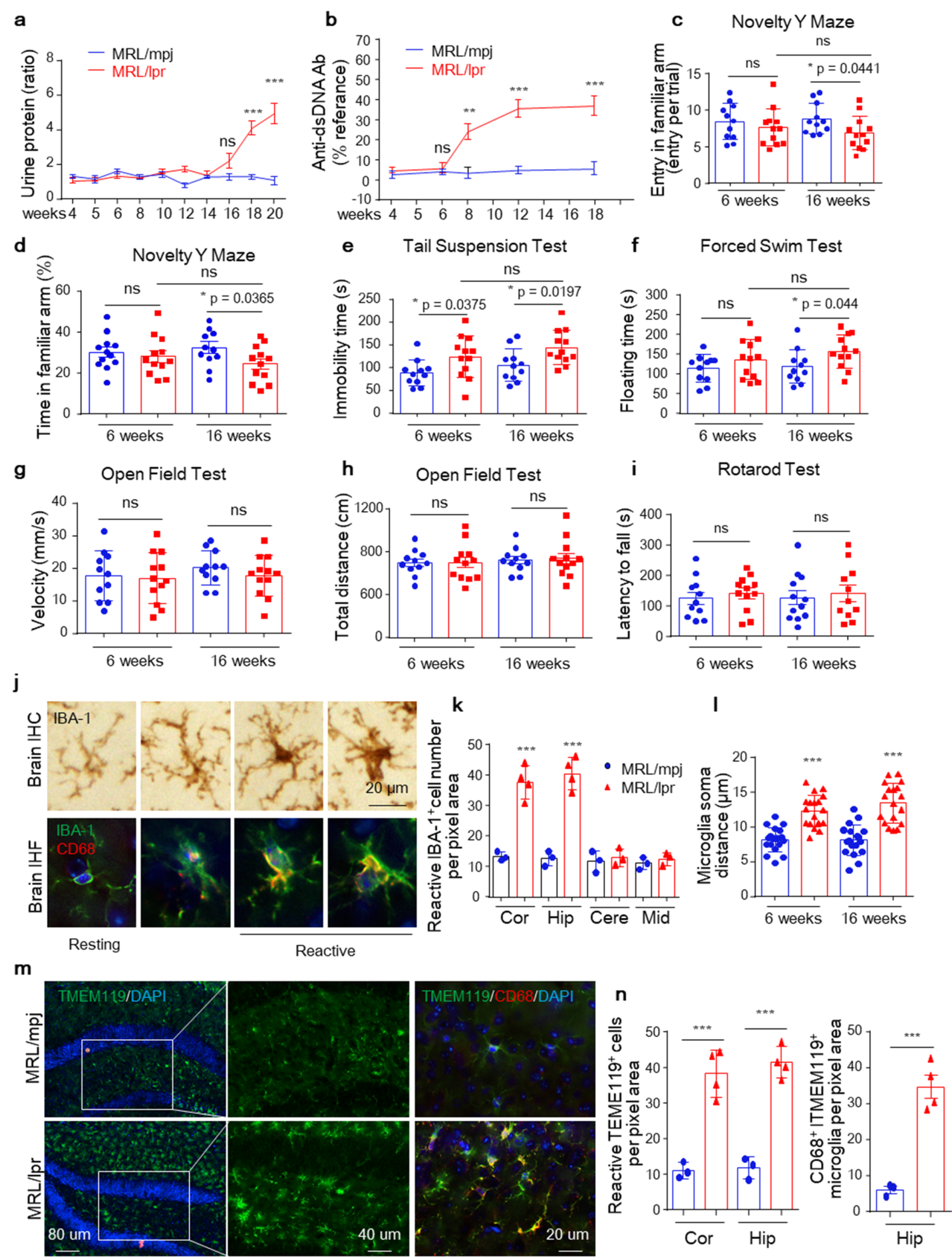
**

**Figure. S1. Behavioral phenotyping, microglial reactivation and systemic disease assessment of MRL/lpr mice, related to Figure 1.**

(**a** and **b**) Proteinuria and autoantibody levels in MRL/lpr and MRL/mpj mice. (**a**) Urine protein concentrations in MRL/mpj and MRL/lpr mice at 4-20 weeks. (**b**) Mouse serum IgG anti-dsDNA titers examined by ELISA from age 4 w to 18 w. *n* = 7-9 (**a**) and *n* = 3-4 (**b**). **P* < 0.05; ***P* < 0.01; ****P* < 0.001 according to ANOVA with Tukey’s post hoc test.

(**c-i**) Behavioral phenotype was assessed from three aspects: motor coordination ability, learning and memory ability, and anxiety- and depression-like mental behaviors. (**c** and **d**) The Novelty Y maze was used to evaluate exploration and cognitive function. Total time in the Novelty Y maze: 3 min. (**e** and **f**) The TST and FST were used to evaluate depression-like behavior. Total time in test: 5 min. (**g-i**) The OFT and rotarod test were used to evaluate general motor ability. The total track lengths and the mean velocity of 6- and 16-week-old MRL/mpj and MRL/lpr mice were measured. Total time in the field: 5 min. Maximum time on the rotarod: 6 min. **P* < 0.05; ***P* < 0.01; ****P* < 0.001 according to unpaired *t*-test. *n* = 11-12 mice/group.

(**j-l**) Microglia indicated by IBA-1^+^ staining and IBA-1/CD68^+^ coimmunostaning were pooled to form resting and reactive microglial populations by morphology. Scale bar, 20 μm. (**k**) Quantitation of microglia in frontal cortex (Cor), hippocampus (Hip), cerebellum (Cere) and midbrain (Mid) sections. *n* = 3-4 mice per group, with an average of 4-5 slices per mouse. (**l**) Diameter of microglial soma in CA3. More than fifteen microglia were quantified per mouse and slice (*n* = 3 mice per group, with 3 - 4 slices per mouse). ns, not significant, **P* < 0.05; ***P* < 0.01; ****P* < 0.0001 according to one-way ANOVA with Tukey’s post hoc test.

(**m** and **n**) Representative images of immunostained hippocampal sections for the microglia specific marker TMEM119, and quantitation of TMEM119^+^, CD68^+^TMEM119^+^ microglia in brain from 6-week-old MRL/mpj and MRL/lpr mice. Scale bars as indicated. *n* = 3-4 mice per group, ****P* < 0.0001 according to unpaired *t*-test.

**
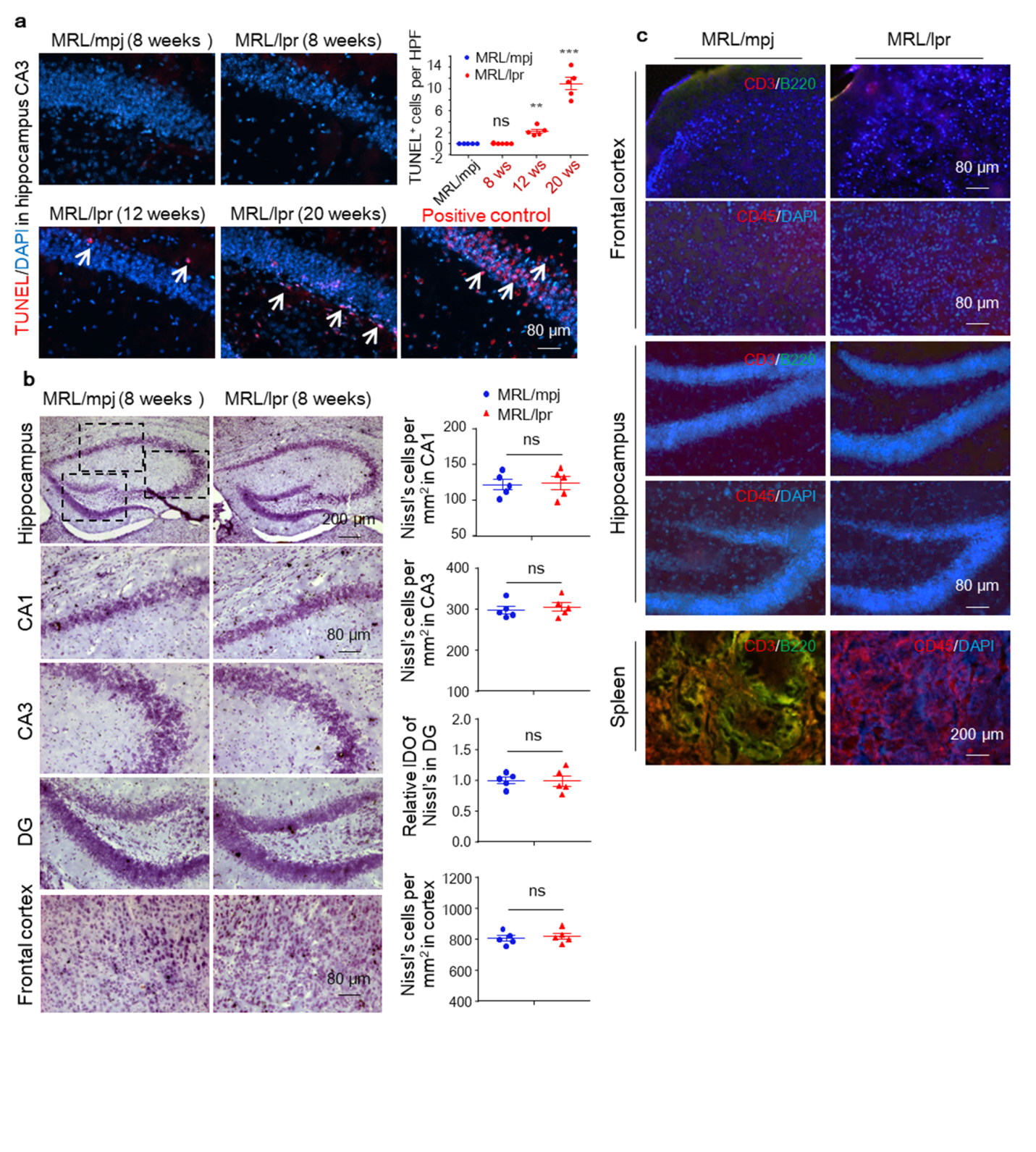
Figure. S2.**

**Figure. S2. MRL/lpr mice do not show appreciable neuron loss, cellular infiltration or astrocyte proliferation in the brain when overt NPSLE develops, related to Figure 1.**

(**a**) Representative images and quantification of TUNEL^+^ apoptotic neurons in hippocampal sections at the indicated time points. Arrow indicates apoptotic neurons. Scale bar, 80 μm. *n* = 5 mice per group, ***P* < 0.01; ****P* < 0.001 according to one-way ANOVA with Tukey’s post hoc test.

(**b**) Immunostaining and quantification of Nissl’s^+^ neurons per mm^2^ within the hippocampus (with amplifications of CA1, CA3 and the dentate gyrus) and the frontal cortex in 8-week-old MRL/lpr and matched control mice. Scale bar as indicated. *n* = 5 mice per group, nonsignificant by Student’s two-tailed t-test.

(**c**) Immunohistochemistry staining of infiltrated B cells or T cells in frontal cortex (above) or hippocampus (DG, middle) sections of 8-week-old MRL/lpr and matched control mice. Spleen sections showed positive staining (representative staining in spleen (below) for T cells (CD3) and B cells (B220), and all bone marrow-derived cells (CD45) showed antibody specificity despite the lack of signal in the brain). Scale bar as indicated.

Data are mean ± SEM.

**
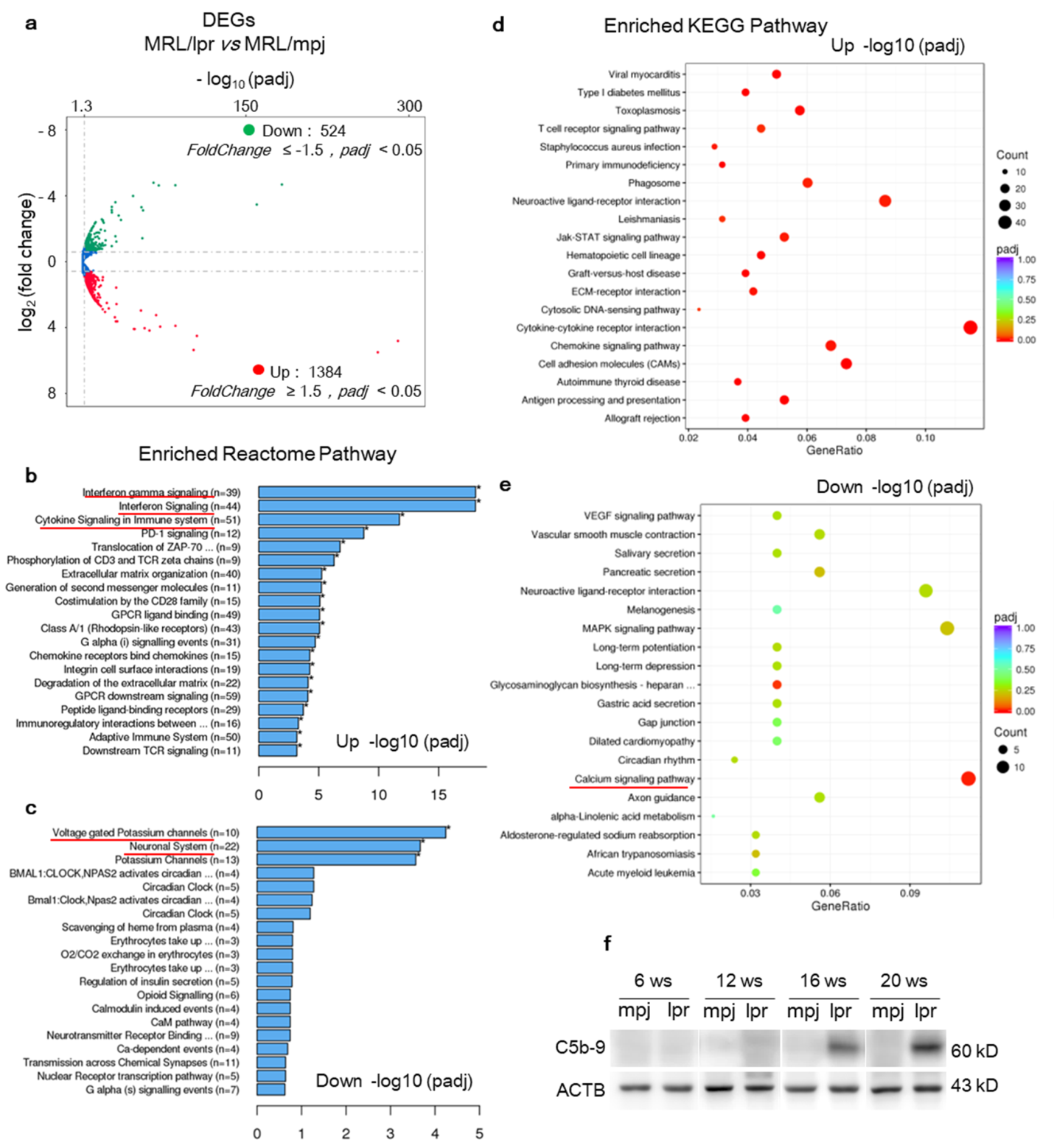
Figure. S3.**

**Figure. S3. RNA-seq and Gene Ontology analysis, related to Figure 2.**

(**a**) Volcano plot of RNA-seq quantified genes with significant fold changes between MRL/mpj and MRL/lpr mice. Only genes identified with a fold change ≥ 1.5 and a *P*-value < 0.05 were regarded as significantly changed.

(**b-e**) Enriched reactome pathway analysis showed upregulated (**b**) and downregulated pathways (**c**) in MRL/lpr mice brain. KEGG pathway analysis of upregulated (**d**) and downregulated pathways (**e**) identified by sequencing.

(**f**) Immunoblotting of C5b-9 in the hippocampus of 6-, 12- 16-, and 20-week-old MRL/lpr and MRL/mpj mice.


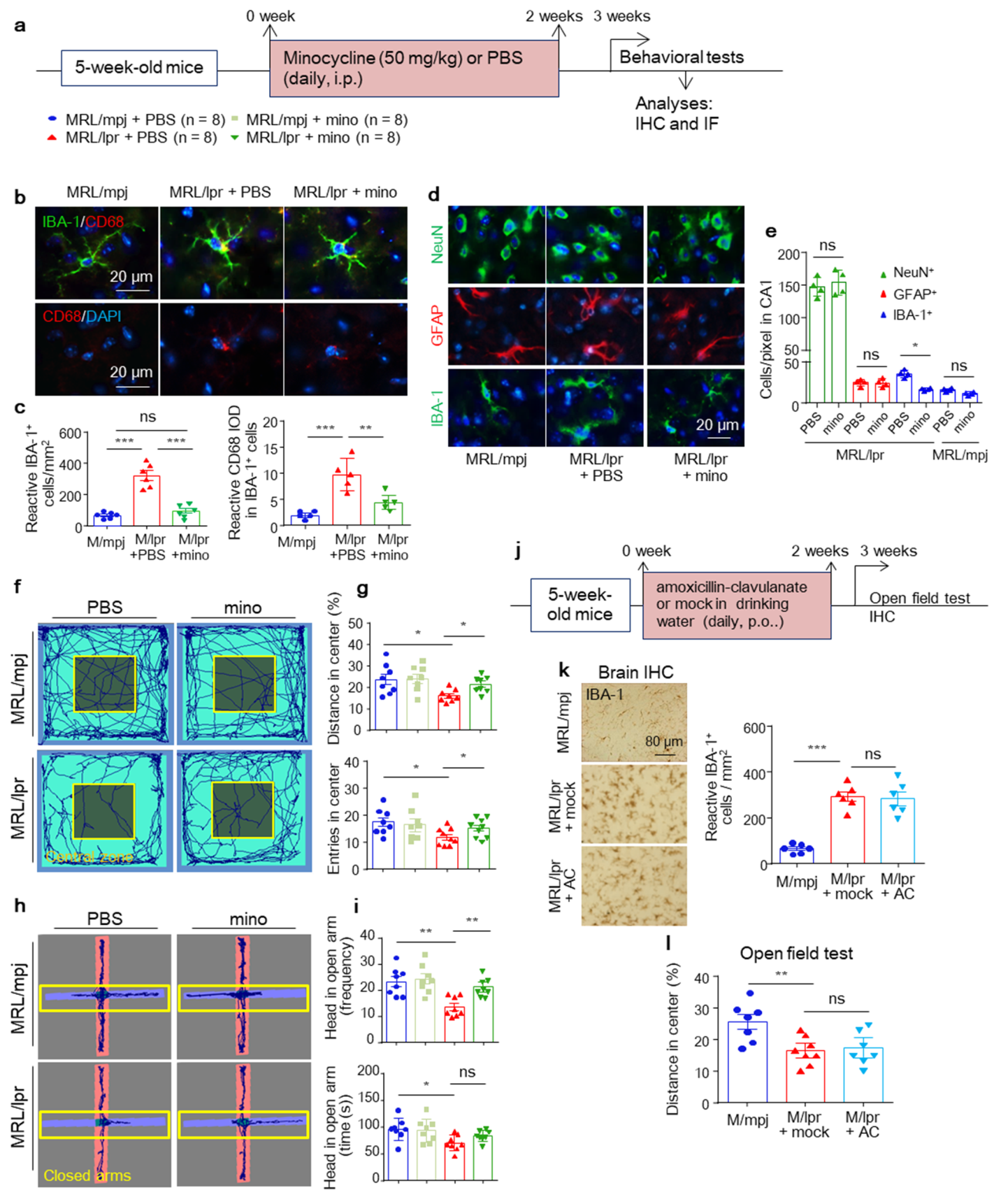
**Figure. S4.**

Figure. S4. Pharmacological inhibition of phagocytosis activation ameliorates NPSLE-related behavioral changes, related to Figure 2.

(**a**) Experimental setup for administration of minocycline (mino, intraperitoneally [i.p.], daily) or PBS in MRL/mpj and MRL/lpr mice.

(**b** and **c**) Representative images and quantification of IBA-1^+^ phagocytes in CA1 regions of the indicated mice. Scale bar as indicated. *n* = 5-6 mice per group.

(**d** and **e**) Representative images and quantification of cells evaluated by immunofluorescence staining for neuronal (NeuN) and glial cell markers (GFAP for astrocytes, IBA-1 for microglia) in hippocampal sections. Scale bar, 20 μm. *n* = 4-5 mice per group.

**(f-i**) Open field test performance (**f**), quantified distance and entries in center (**g**), elevated plus maze test performance (**h**), quantified frequency and time of head in open arm (**i**) at the end of the experiment (8-week-old). Total time in the field: 5 min, Total time in the maze: 6 min. *n* = 8 mice per group.

(**j**) Experimental setup for administration of amoxicillin-clavulanate (AC, oral, daily).

(**k**) Representative images and quantification of IBA-1^+^ microglia in CA1 regions of the indicated mice. Scale bar as indicated. *n* = 6 mice per group.

**(l**) Open field test performance, quantified distance in center, at the end of the experiment in mice from **j**. Total time in the field: 5 min. *n* = 8 mice per group.

Data are the mean ± SEM. **P* < 0.05; ***P* < 0.01; ****P* < 0.001; and ns, not significant according to one-way ANOVA with Tukey’s correction for multiple comparisons.

**Figure. S5.
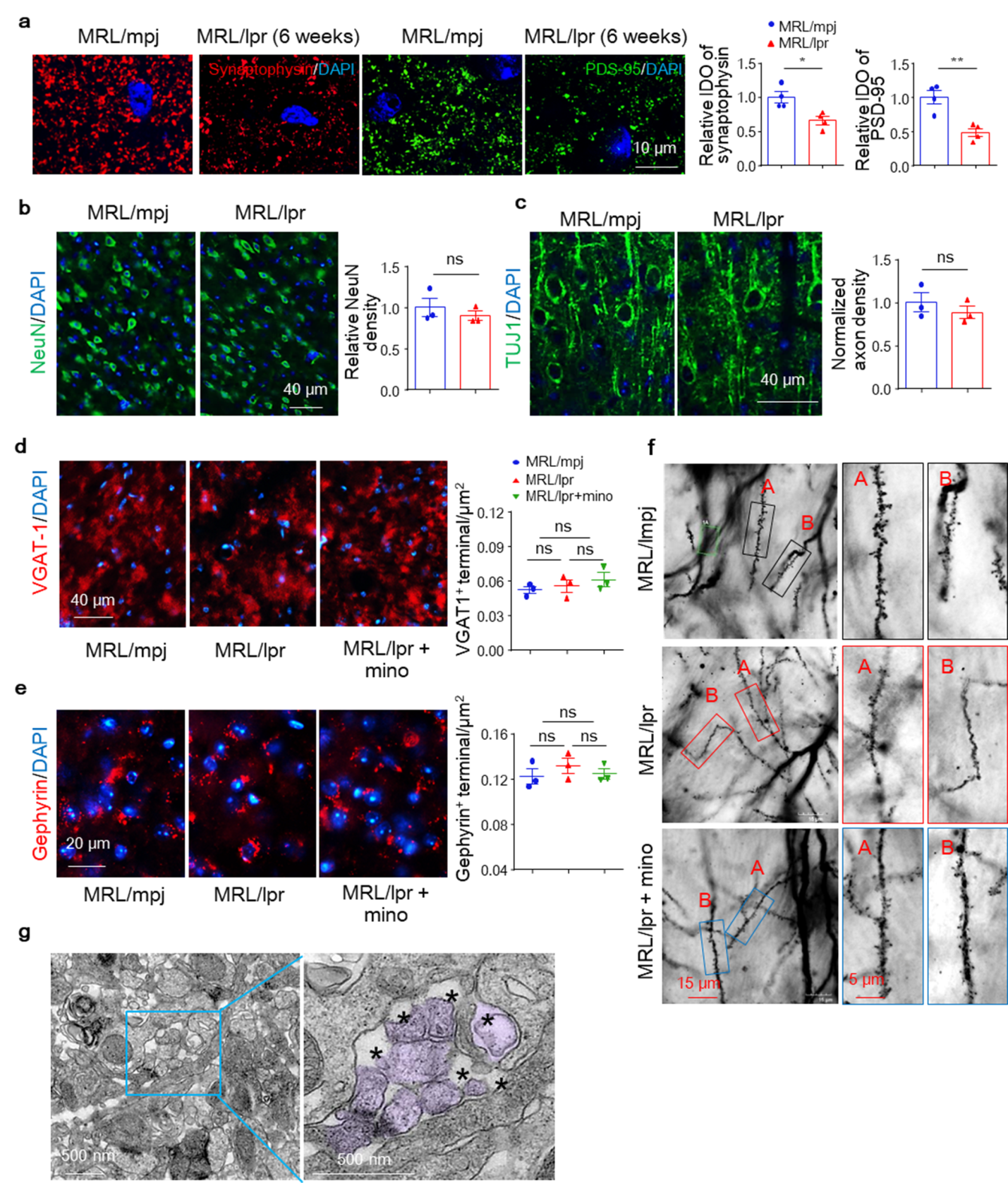
**

Figure. S5. Synapse loss, but not neuron or axon defects, is observed in the brains of MRL/lpr mice, related to Figure 3.

(**a**) Staining and quantification of presynaptic (synaptophysin) and postsynaptic (PSD-95) markers revealed structural synapses. Data are the mean of 3 staining experiments. *n* = 4 mice per group, **P* < 0.05; ***P* < 0.01 according to unpaired *t*-test. Scale bar, 10 μm.

(**b** and **c**) Staining for the neuronal marker NeuN (**b**) and axonal marker TUJ1 (**c**) in hippocampal sections. *n* = 3 mice per group, *P* > 0.05 (not significant), unpaired *t*-test.

(**d** and **e**) Confocal immunofluorescence images and quantification of the staining area for inhibitory presynaptic terminals (VGAT-1) and inhibitory postsynaptic terminals (Gephyrin) in the pyramidal layer of CA1, in MRL/mpj or MRL/lpr mice treated with or without minocycline (8-week-old). Scale bar as indicated. *n* = 3 mice per group, ns, not significant according to one-way ANOVA with Tukey’s test.

(**f**) Representative images of Golgi-stained dendritic spines from DG granule neurons in MRL/mpj and MRL/lpr mice treated with or without minocycline. Scale bars as indicated.

(**g**) TEM revealed synaptic inclusions within microglial cytoplasm (blue box) and proteolytic lysosomes (black asterisk) at higher magnification, with some inclusion-containing structures, consistent with synaptic vesicles (purple) in the hippocampus of MRL/lpr mice.

Data expressed as the mean ± SEM.

**Figure. S6.
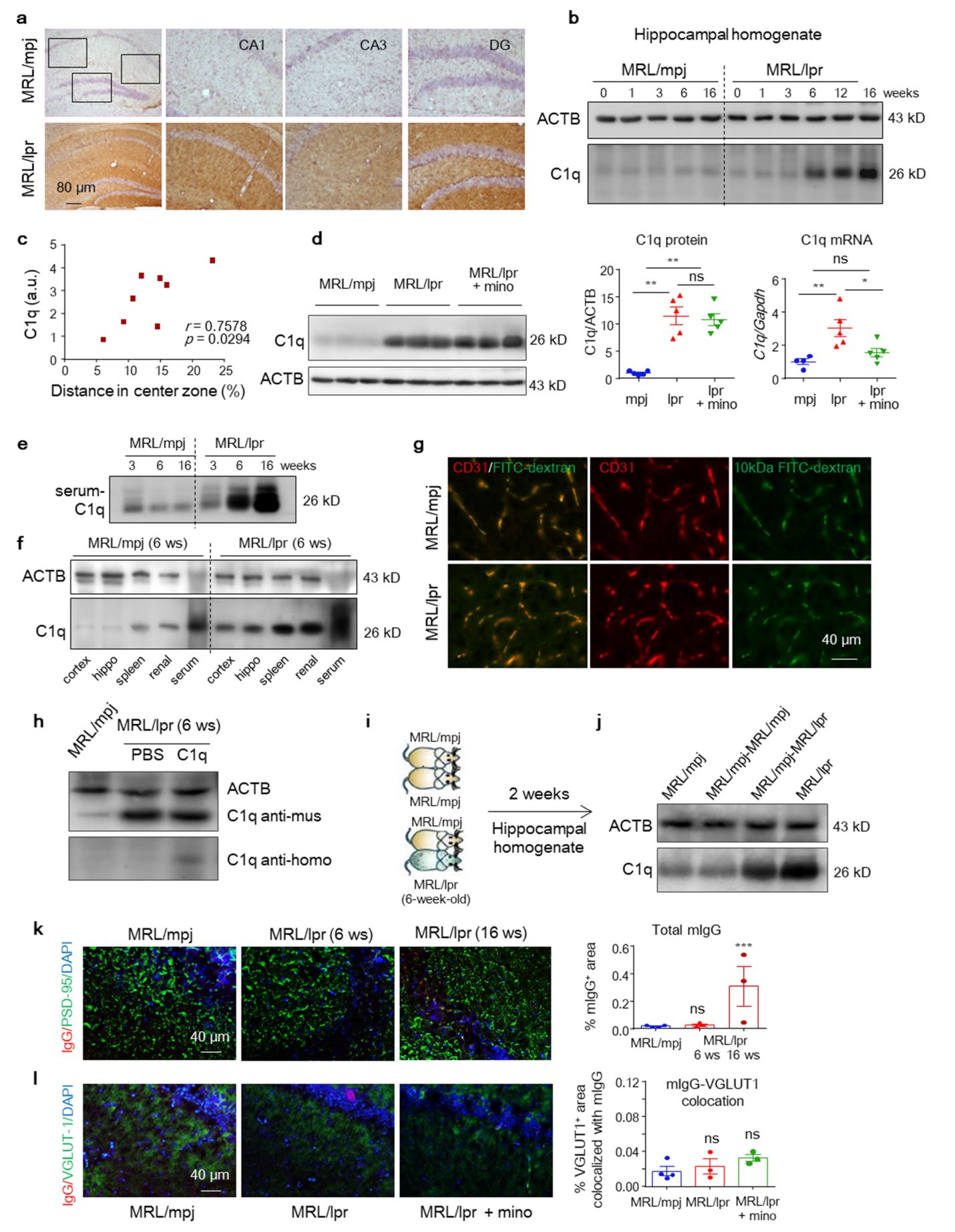
**

Figure. S6. Major serum-sourced, synapse-deposited C1q rather than IgG correlates with behavioral defects in MRL/lpr mice, related to Figure 4.

(**a** and **b**) (**a**) Representative image of anti-C1q immunostaining in the hippocampus of 6-week-old MRL/lpr and MRL/mpj mice. Scale bar, 80 μm. (b) Immunoblotting of C1q in the hippocampus of 0-, 1-, 3-, 6-, 12- and 16-week-old MRL/lpr and MRL/mpj mice.

(**c**) Correlation of C1q and behavioral performance in the OFT of MRL/lpr mice at 6 weeks of age (a.u., arbitrary units).

(**d**) Immunoblotting and quantification of C1q protein and mRNA levels in the hippocampus of MRL/mpj and MRL/lpr mice treated with or without minocycline (mino). *n* = 4-5 mice per group. **P* < 0.05; ***P* < 0.01 according to one-way ANOVA with Tukey’s multiple comparisons test.

(**e** and **f**) (**e**) Immunoblotting of C1q in the serum of 3-, 6-, and 16-week-old MRL/lpr and MRL/mpj mice. (**f**) WB image demonstrating the burden of C1q in the cortex, hippocampus (hippo), peripheral tissues, and sera of 6-week-old MRL/mpj and MRL/lpr mice (*n* = at least 3 biologically independent samples).

(**g** and **h**) Intravenous injection of 40-kDa FITC-dextran tracer revealed no leakage of the dye from blood vessels (CD31^+^) in MRL/lpr mice at 6 weeks of age. (**g**) Colocalization of FITC-dextran with CD31 (blood vessels) showed that similar colocalization was observed in MRL/lpr mice and controls. *n* = 3 mice per group, P > 0.05, unpaired t-test. (**h**) Western blotting analysis of C1q revealed an increase in injected C1q deposition within the CNS in MRL/lpr mice compared to mock-injected and MRL/mpj controls.

(**i** and **j**) (**i**) Scheme illustrating parabiosis. (**j**) WB analysis confirmed that the C1q protein was increased in the hippocampus of MRL/mpj mice in the MRL/lpr parabiosis model.

(**k** and **l**) (**k**) Immunostaining for PSD-95 and endogenous murine IgG (mIgG) in MRL/mpj or 6- and 16-week-old MRL/lpr mice. Quantification was performed on the total percent of mIgG staining area as well as the percent of PSD-95^+^ staining area colocalized with mIgG. *n* = 3-4. (**l**) Immunostaining for VGLUT1 and endogenous mIgG in 8-week-old MRL/mpj or MRL/lpr mice treated with or without minocycline. *n* = 3-4. Quantification was performed on the total percent of mIgG staining area as well as the percent of VGLUT1 staining area colocalized with mIgG. Significance was determined by Student’s *t*-test.

**Figure. S7.
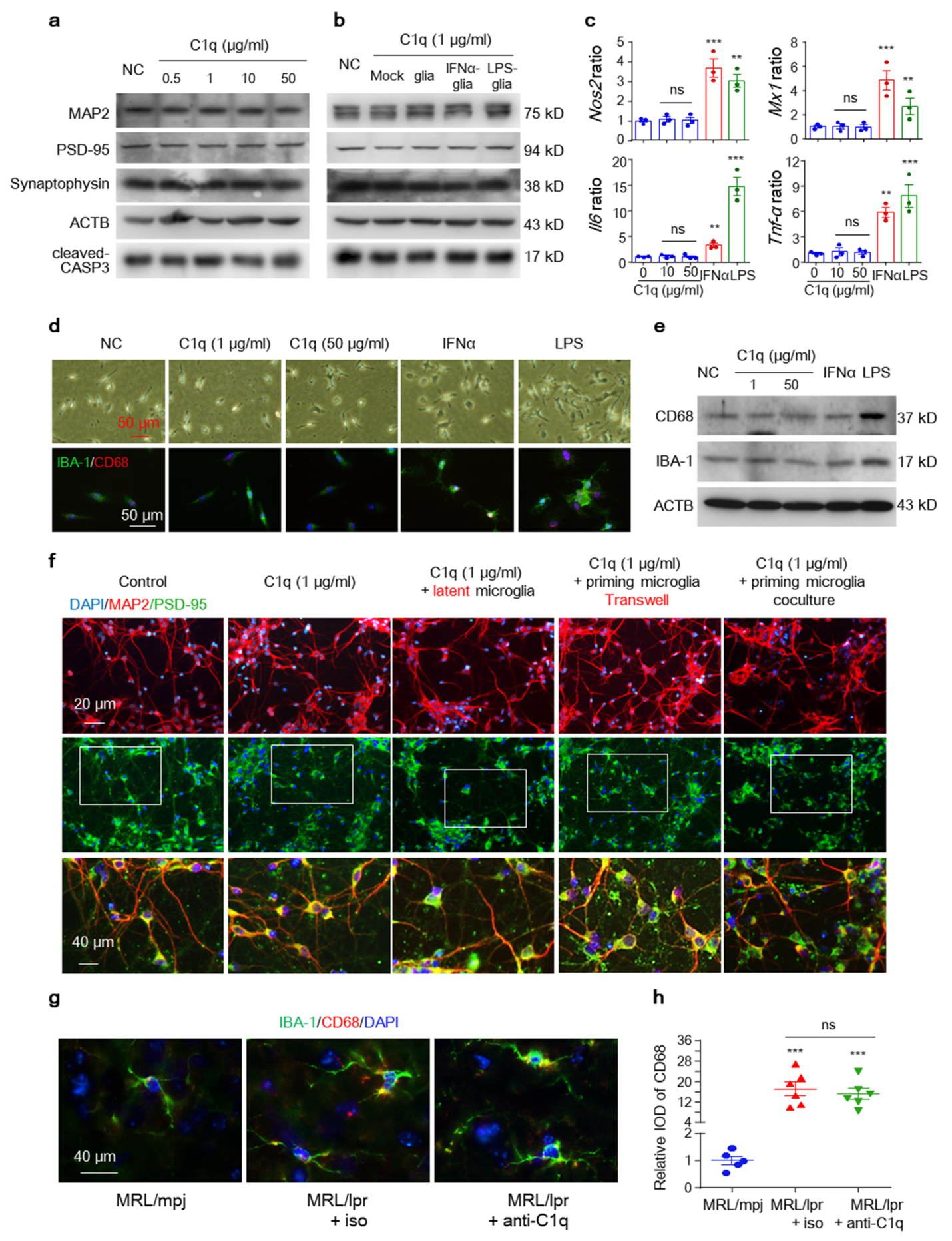
**

Figure. S7. C1q coordinates activated microglia pruning neuronal synapses via direct contact, related to Figure 5.

**(a** and **b**) MRL/mpj mouse-derived hippocampal neurons were cultured in serum-free medium to avoid contamination by C1q in serum and were incubated with exogenous C1q (concentration: 0.5-50 μg/ml) (**a**) or incubated with exogenous C1q (1 μg/ml) added to Transwell inserts cultured with different primed microglia (**b**) for 24 h. All cell lysates were harvested for MAP2, PSD-95, synaptophysin and cleaved CASP3 Western blotting.

(**c**) Messenger RNA (mRNA) levels of *Nos2*, *Mx1*, *Il6*, and *Tnf-α* in microglia stimulated with IFNα (10 ng/mL), LPS (100 ng/mL) or various concentrations of C1q (μg/mL) for 6 h in serum-free medium (at least 3 biologically independent samples).

(**d** and **e**) Representative images of microglial morphology and immunoblots of IBA-1 or CD68 in microglia treated with C1q (0-50 μg/ml), IFNα (10 ng/mL) or LPS (100 ng/mL) for 24 h. Scale bar, 50 μm.

(**f**) Primary neurons of MRL/lpr mice were cocultured (directly or transwell) with C1q and latent or primed microglia and stained for PSD-95 (green) and MAP2 (red) 3 days later. Scale bar as indicated.

(**g** and **h**) Representative images and quantification of CD68 intensity in IBA-1^+^ microglia in brain sections of MRL/mpj and MRL/lpr mice treated with either isotype (iso) or anti-C1q antibody (anti-C1q). Scale bar, 40 μm. *n* = 5-6 mice per group. The data shown are representative of three independent experiments.

Data are mean ± SEM. ***P* < 0.01; ****P* < 0.001; and ns, not significant according to one-way ANOVA with Tukey’s multiple comparisons test in (**c**), (**h**).

**Figure. S8.**

**
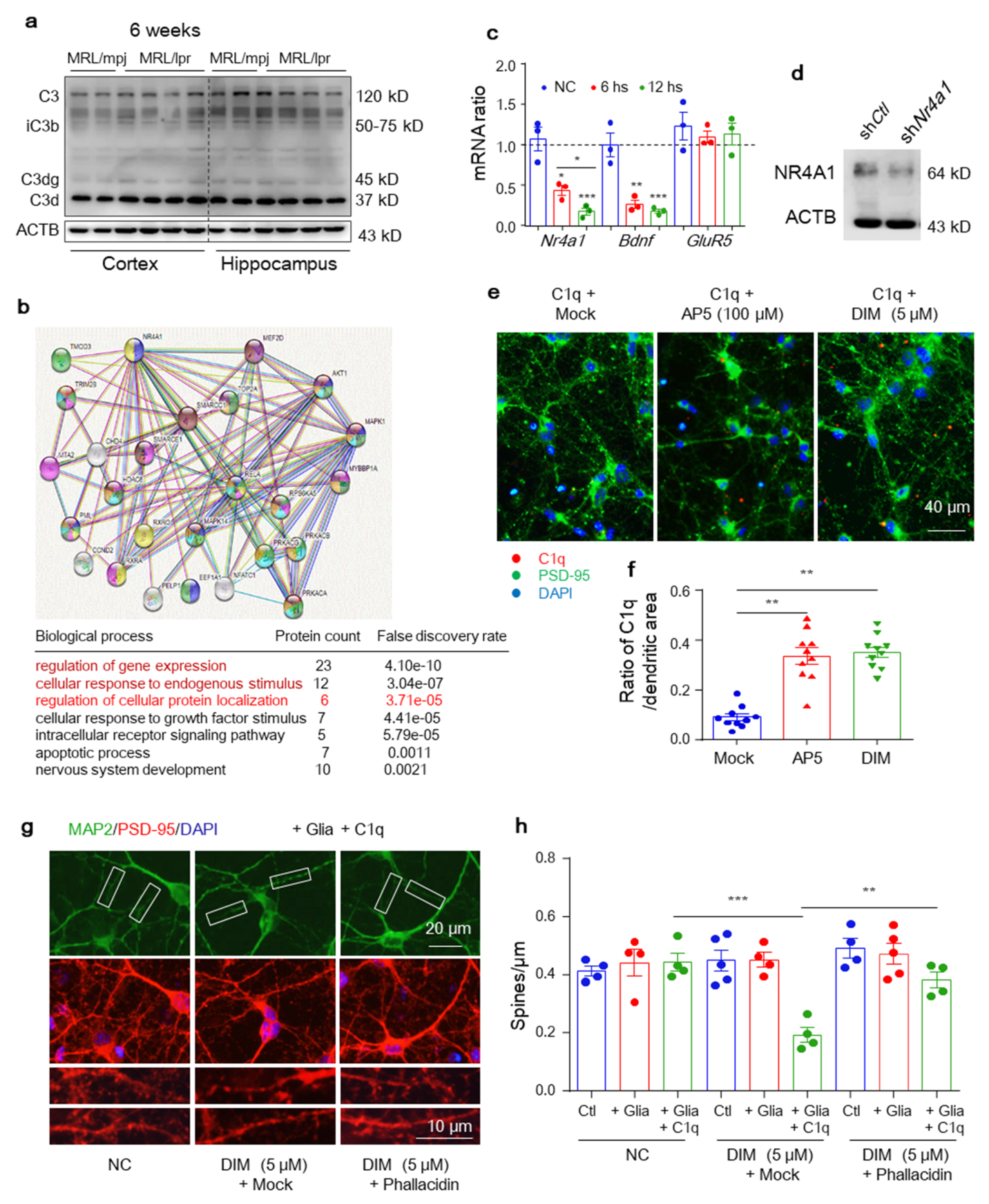
**

Figure. S8. Reduced Nr4a1-mediated C1q tag at synapses in neurons of MRL/lpr mice, related to Figure 6.

(**a**) Immunostaining of C3 and active segments in the brains of MRL/mpj and MRL/lpr mice at 6 weeks of age.

(**b**) Network analysis of the NR4A1, MED2, and TMOD interactomes using the STRING tool. Connecting lines indicate direct or functional interactions of the proteins. Gene ontology analysis by STRING shows biological processes that are enriched in the protein network.

(**c** and **d**) mRNA changes in the indicated genes caused by 6 or 24 h of AP5 (100 μM) treatment. Fold changes are normalized to vehicle-treated controls (Student’s t-test, data shown are representative of three independent experiments). (**d**) Immunoblotting of NR4A1 from shRNA-treated primary hippocampal neuron lysates. ACTB served as a loading control.

(**e** and **f**) Representative images of dendritic segments and C1q colocation after treatment of hippocampal neurons with NMDAR antagonist AP5 (100 µM) or with the NR4A1 antagonist DIM-C-pPhCO2Me (DIM, 5 µM) for 24 h. Scale bar, 40 μm.

(**g** and **h**) Dendritic segments of neurons after treatment with control or DIM (5 µM) and incubation with phallacidin or vehicle (**g**). Scale bar as indicated. The histogram shows the spine density along dendrites (**h**).

Data are mean ± SEM. ***P* < 0.01; ****P* < 0.001 according to one-way ANOVA with Tukey’s multiple comparisons test in (**f**), (**h**).

**Figure. S9.**
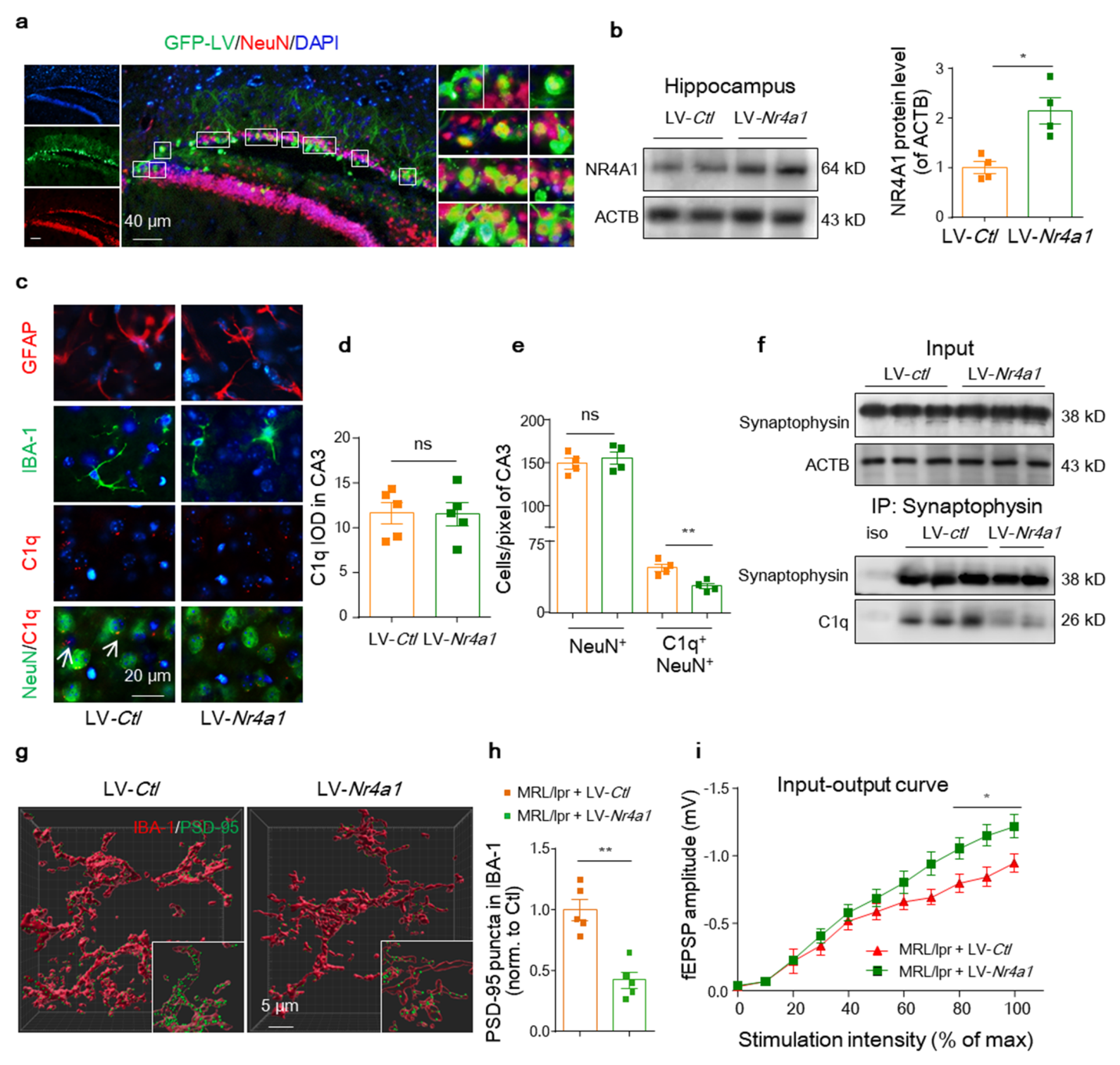


Figure. S9. Overexpression of Nr4a1 in hippocampal neurons, related to Figure 7.

(**a**) Representative images of MRL/lpr mice microinjected with either control or *Nr4a1*-GFP lentivirus (1 μl of 1×10^9^ viral genomes/µl) into the hippocampus. Scale bar, 40 μm.

(**b**) Expression of NR4A1 in the hippocampus of mice microinjected with lentivirus was determined by immunoblotting. *n* = 4 mice/group, **P* < 0.05 versus control using Student’s *t*-test.

(**c-e**) Experimental setup as in Fig. 7a. Mice were microinjected with the *control/Nr4a1*-GFP lentivirus in the hippocampus. Three weeks later, the hippocampal samples were collected for IHF/WB analysis. Representative images and quantification of neuronal (NeuN) and glial markers (GFAP for astrocytes and IBA-1 for microglia) and C1q intensity with NeuN in hippocampal sections, *n* = 4-5 mice per group. Scale bar, 20 μm. ns, not significant, ***P* < 0.01 according to one-way ANOVA with Tukey’s correction for multiple comparisons. The data shown are representative of three independent experiments.

(**f**) Coimmunoprecipitation analysis of C1q with synaptophysin antibodies from control or sh*Nr4a1* lentivirus injected MRL/lpr hippocampal lysates.

(**g** and **h**) Representative confocal stacks of IBA-1 (red) and engulfed PSD-95 (green) puncta in the CA1 region of MRL/lpr mice injected with control or sh*Nr4a1* lentivirus. Scale bar, 5 μm. (**h**) Quantitation of the relative number of engulfed PSD-95 in IBA-1^+^ cells normalized to LV-*Ctl*-injected mice. 15-18 cells/group, with 5 mice per group. ***P* < 0.01 versus control according to Student’s *t*-test.

(**i**) Input–output relationship measuring basal synaptic function in LV-*Ctl*- and LV-*Nr4a1*-injected MRL/lpr mice (*n* = 8 - 10 slices from three mice per group), **P* < 0.05 according to two-way ANOVA, Bonferroni’s test.

Data are the mean ± SEM.

**Figure. S10.**

**
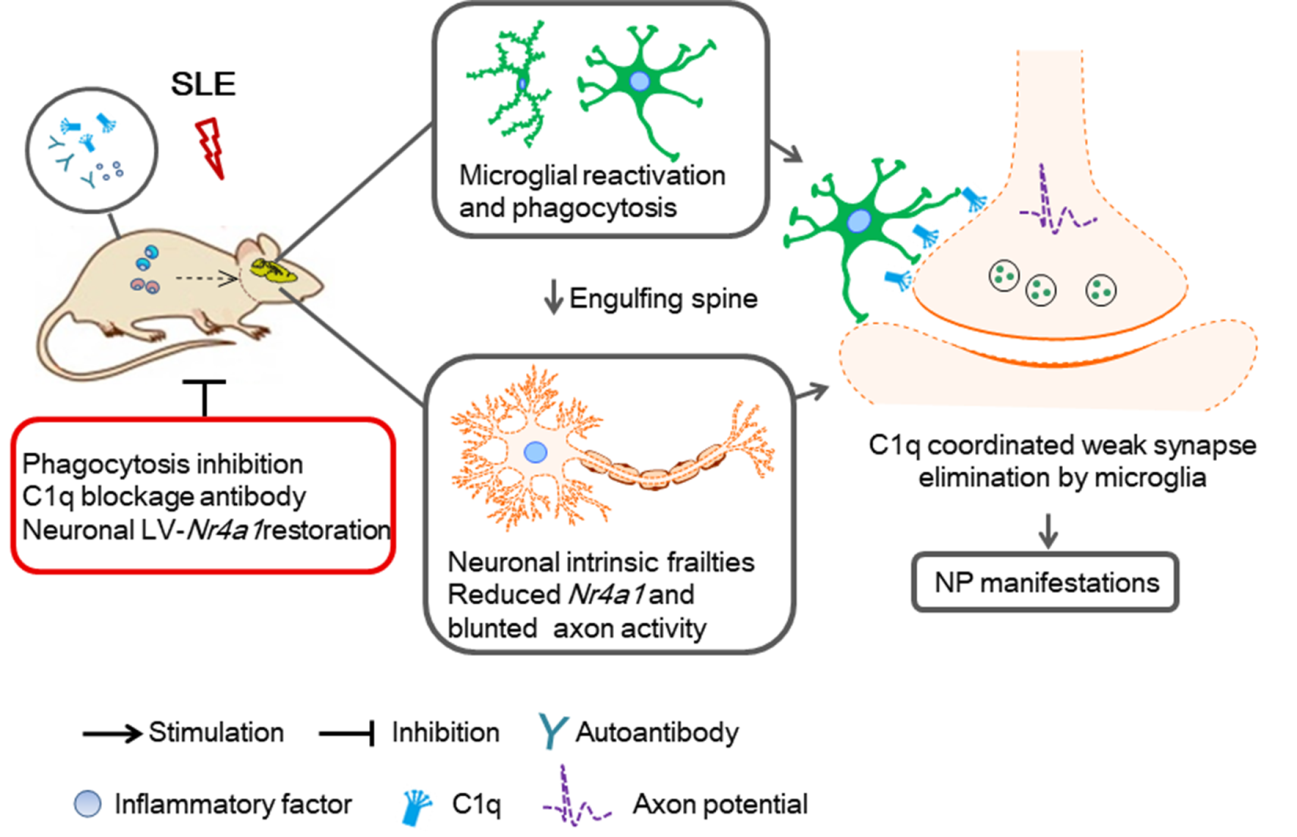
**

**Figure. S10. Graphical Abstract: Schematic of neuron–complement–microglia-axis-coordinated synaptic loss in CNS lupus.**

In brief, our data inform a model in which neurons act as intermediaries between complement and microglia, synergistically driving their own synaptic loss, and subsequently regulating neuropsychiatric behaviors in lupus mice.

**Table. S1. Biological pathways enriched in MRL/mpj versus MRL/lpr mice by GSEA analysis with KEGG modules. Top 20 pathways of GSEA using gene sets derived from KEGG pathway database with false discovery rate (FDR) q-value <0.05 in MRL/mpj versus MRL/lpr mice**

Pathways genes P-value P-adj Select genes within pathway

| Cytokine-cytokine receptor interaction | 45 | 1.48E-07 | 2.8E-05 | Ccl8/Kdr/Cxcl13/Cxcl10/Ccl5/Ngfr/Ccr1/Tnfsf10/Ltb/Ccr2/Il3ra/Tgfbr2/Cxcl11/Kitl/Il18r1/Tnfrsf1b/Ctf1/Cxcr4/Ccl3/Cxcl16/Il2rb/Il4ra/Ghr/Pdgfc/Il9r/Il10rb/Bmp2/Met/Il21r/Il13ra1/Cxcr6/Lepr/Ccr5/Csf2rb/Cxcl9/Kit/Edar/Ccr7/Xcr1/Ccl28/Tnfrsf14/Cxcr3/Cd27/Tnfsf8/Ifng |
| --- | --- | --- | --- | --- |
| Antigen processing and presentation | 21 | 7.55E-07 | 7.13E-05 | H2-Aa/H2-Ab1/B2m/Cd74/H2-Eb1/H2-T22/H2-T9/Tap1/Hspa1b/H2-T23/ Cd8a/Cd4/Ciita/Tap2/H2-M2/Tapbp/H2-DMb2/H2-T24/H2-DMb1/H2-K1/Ifng |
| Allograft rejection | 15 | 6.21E-06 | 0.000265 | H2-Aa/H2-Ab1/H2-Eb1/H2-T22/H2-T9/H2-T23/H2-M2/H2-DMb2/H2-T24/Cd28/Prf1/Gzmb/H2-DMb1/H2-K1/Ifng |
| Graft-versus-host disease | 15 | 6.21E-06 | 0.000265 | H2-Aa/H2-Ab1/H2-Eb1/H2-T22/H2-T9/H2-T23/H2-M2/H2-DMb2/H2-T24/Cd28/Prf1/Gzmb/H2-DMb1/H2-K1/Ifng |
| Type I diabetes mellitus | 16 | 7.76E-06 | 0.000265 | H2-Aa/H2-Ab1/H2-Eb1/H2-T22/H2-T9/H2-T23/Ica1/H2-M2/H2-DMb2/H2-T24/Cd28/Prf1/Gzmb/H2-DMb1/H2-K1/Ifng |
| Viral myocarditis | 21 | 8.41E-06 | 0.000265 | H2-Aa/H2-Ab1/Casp9/Myh7/H2-Eb1/H2-T22/H2-T9/Itgal/H2-T23/Rac2/H2-M2/Casp3/H2-DMb2/Myh11/H2-T24/Cd28/Myh7b/Prf1/H2-DMb1/H2-K1/Casp8 |
| Neuroactive ligand-receptor interaction | 45 | 2E-05 | 0.000541 | Htr2c/Mchr1/Grin1/Avpr1a/Crhr2/Ntsr2/Adra2b/Cnr1/Trhr/Chrm3/Chrna5/Grm8/Gabra5/Npffr1/Drd2/Grik3/Glp1r/S1pr3/Sstr4/Mc3r/Tspo/Htr2a/Adra2a/Htr5b/Gabre/Galr1/Glra1/Calcrl/Lpar6/Ghr/Gpr35/Htr7/Oprd1/Lhb/Sstr5/Ptafr/Chrm1/Oxtr/Grm4/Gpr50/Lepr/Agtr1a/Oprk1/Grin2d/Hrh1 |
| Cell adhesion molecules (CAMs) | 29 | 2.29E-05 | 0.000541 | H2-Aa/H2-Ab1/Cldn5/Ptprc/H2-Eb1/H2-T22/H2-T9/Itgal/Cd34/H2-T23/Sdc2/Cldn3/Cd8a/Cd2/Cd274/Cd4/H2-M2/Spn/H2-DMb2/Itgb7/Cldn19/H2-T24/Cd28/Pdcd1/F11r/H2-DMb1/H2-K1/Cdh4/Cd6 |
| Primary immunodeficiency | 12 | 3.59E-05 | 0.000755 | Ptprc/Tap1/Cd3e/Cd8a/Cd3d/Cd4/Ciita/Tap2/Cd79a/Lck/Cd19/Jak3 |
| Autoimmune thyroid disease | 14 | 5.42E-05 | 0.001024 | H2-Aa/H2-Ab1/H2-Eb1/H2-T22/H2-T9/H2-T23/H2-M2/H2-DMb2/H2-T24/Cd28/Prf1/Gzmb/H2-DMb1/H2-K1 |
| Toxoplasmosis | 26 | 8.29E-05 | 0.001323 | H2-Aa/H2-Ab1/Pla2g4e/Casp9/Lama3/Igtp/Pla2g5/H2-Eb1/Pla2g2f/Hspa1b/Bcl2/Nfkbia/Tlr2/Ciita/Socs1/Stat1/Casp3/H2-DMb2/Il10rb/Pla2g2d/Lamc2/Ccr5/H2-DMb1/Lamb3/Casp8/Ifng |
| Hematopoietic cell lineage | 19 | 8.4E-05 | 0.001323 | Cd3g/H2-Eb1/Cd34/Cd24a/Cd3e/Il3ra/Cd8a/Cd2/Cd3d/Cd5/Kitl/Cd4/Cd44/ Itga2/ Cd59b/Il4ra/Il9r/Cd19/Kit |
| Phagosome | 28 | 0.000756 | 0.010985 | H2-Aa/H2-Ab1/H2-Eb1/H2-T22/H2-T9/Thbs4/Tap1/Dync1i1/H2-T23/ Cybb/Clec7a/Thbs1/Atp6v1g2/Cyba/Tlr2/Tap2/H2-M2/Itga2/H2-DMb2/ Tubb6/H2-T24/C1ra/Cd209b/Cd209a/Fcgr4/H2-DMb1/Ncf2/H2-K1 |
| Calcium signaling pathway | 29 | 0.001828 | 0.022957 | Htr2c/Pde1a/Itpka/Prkcg/Atp2b1/Camk4/Grin1/Ppp3r1/Avpr1a/Itpkb/Adcy9/Prkcb/Trhr/Adcy7/Chrm3/Atp2b4/Gna14/Cacna1g/Htr2a/Plcb4/Htr5b/Htr7/Pde1c/Ptafr/Chrm1/Oxtr/Agtr1a/Grin2d/Hrh1 |
| Chemokine signaling pathway | 29 | 0.001999 | 0.022957 | Ccl8/Cxcl13/Cxcl10/Adcy9/Ccl5/Prkcb/Grk5/Ccr1/Adcy7/Rac2/Ccr2/Cxcl11/Nfkbia/Stat1/Plcb4/Cxcr4/Hck/Ccl3/Cxcl16/Jak3/Cxcr6/Ccr5/Gnb4/Cxcl9/Ccr7/Xcr1/Prkcd/Ccl28/Cxcr3 |
| ECM-receptor interaction | 17 | 0.002175 | 0.022957 | Spp1/Lama3/Col6a3/Thbs4/Sdc2/Sv2c/Thbs1/Col5a2/Sv2b/Cd44/Itga2/Itgb7/Col4a4/Col5a3/Lamc2/Lamb3/Itgb6 |
| T cell receptor signaling pathway | 20 | 0.002179 | 0.022957 | Ppp3r1/Cd3g/Ptprc/Tec/Cd3e/Cd8a/Rasgrp1/Cd3d/Nfkbia/Cd4/Lat/Cd247/Cd28/Pdcd1/Lck/Card11/Prkcq/Ptpn6/Ifng/Grap2 |
| Leishmaniasis | 14 | 0.002186 | 0.022957 | H2-Aa/H2-Ab1/H2-Eb1/Prkcb/Cyba/Nfkbia/Tlr2/Stat1/H2-DMb2/Fcgr4/H2-DMb1/Ncf2/Ptpn6/Ifng |
| Cytosolic DNA- sensing pathway | 10 | 0.004014 | 0.039927 | Cxcl10/Ccl5/Zbp1/Nfkbia/Irf7/Mavs/Ddx58/Ifi202b/LOC100044068/Casp1 |
| Staphylococcus aureus infection | 11 | 0.005153 | 0.048696 | H2-Aa/H2-Ab1/H2-Eb1/Itgal/C1s1/H2-DMb2/C1ra/Ptafr/Fcgr4/H2-DMb1/Hc |

**Table. S2. Oligonucleotide primers used for mRNA real-time PCR**

| Gene | Sense primer (5’ to 3’) | Antisense primer(5’ to 3’) | |  |
| --- | --- | --- | --- | --- |
| *Cx3cr1*  *Tlr2*  *Tap1*  *Fcgr2b*  *Itga2*  *Thbs1*  *C1qa*  *C1qc*  *Cfp*  *C1 s1*  *C3*  *C6*  *Nr4a1*  *IL-6*  *TNFα*  *IFNγ*  *MX-1*  *Gapdh* | CGCCAACTCCATGAACAACC  CGGACTGTTTCCTTCTGACCA  AGCGGCAACCTTGTCTCATT  GTCACAGAAGTGGACGGAGTG  TGGTAGTTGTGACCGATGG  AACCAGTGTGTGGAAAGGCA  AGGACTGAAGGGCGTGAAAG  ATCCATCGGCCCTGTATCTC  TTGCTTCCATTCTGACCCCC  ATGACCAGAGGCAGGAGAGG  CCCCTTACCCCTTCATTCCTT  CTTAGCGTGCGTGTCTTTGG  GAAAGTTGGGGGAGTGTGCT  CCCCAATTTCCAATGCTCTCCT  TTGCTCTGTGAAGGGAATGG  GGGTGGGGAAGAGATTGTCC  TCATCAGAGTGCAAGCGAGG  CCAGCTACTCGCGGCTTTA | | GATGAGTCTGACGGCTCTGG  ACGCTTTGTCTGAGGTTTCG  ACCTGCACTTTGAATCAGGTGGTC  ATGTTTGAGGGTGCCAACGA  CTGCTATGCCGAACCTCAGT  CTGCTATGCCGAACCTCAGT  GGGAAGCCAAAGGAGTCCAA  TTTCTCTATCTCACCACCTGAAC  CAGGTTCCCCCTTCGTCATT  ACCCAGGGCTCAGTGTCA  GCCGTAGGACATTGGGAGTA  TCCAGACCCTTCAAATCGACC  GGGTCTCATCTAATGGGCCG  CATAACGCACTAGGTTTGCCG  GGCTCTGAGGAGTAGACAATAAAG  CCCAGATACAACCCCGCAAT  GGCTGTCTCCCTCTGATACG  GTTCACACCGACCTTCACCA | |

**Table. S3. Key resources table**

| **Reagent or resource** | **Source** | **Identifier** |
| --- | --- | --- |
| **Antibodies** |  |  |
| beta-actin (HRP-conjugate) | Sigma-Aldrich | Cat# AB_262011 |
| C1q | Abcam | Cat# ab71940 |
| C1q | Santa Cruz Biotechnology | Cat# sc-53544 |
| C1q | Santa Cruz Biotechnology | Cat# sc-58920 |
| C1q | Proteintech | Cat# 11602-1-AP |
| C1q-blocking antibody | This paper | Dejanovic, B. et al. Neuron |
| CD68 | Bio-Rad | Cat# AB_322219 |
| CD68 | Proteintech | Cat# 66231-2-Ig |
| LAMP-1 | R&D Systems | Cat# AF4320-SP |
| LAMP-1 (clone 1D4B) | Santa Cruz Biotechnology | Cat# sc-19992 |
| IBA-1 | Wako | Cat# 019-19741 |
| TMEM119 | Abcam | Cat# ab209064 |
| GFAP | Abcam | Cat# ab7260 |
| MAP2 | Abcam | Cat# ab11267 |
| MAP2 | Proteintech | Cat# 17490-1-AP |
| beta III Tubulin (TUJ1) | Abcam | Cat# 78078 |
| NeuN | Millipore | Cat# MAB377 |
| NeuN | Abcam | Cat# ab177487 |
| PSD-95 | Abcam | Cat# ab2723 |
| PSD-95 | Proteintech | Cat# 20665-1-AP |
| Synaptophysin | Proteintech | Cat# 17785-1-AP |
| Synaptophysin | Synaptic Systems | Cat. No. 101 002 |
| VGLUT1 | Synaptic Systems | Cat# N1602-At488-S |
| VGAT-1 | Millipore | Cat# AB1570W |
| Gephyrin (clone G-6) | Santa Cruz Biotechnology | Cat# sc-25311 |
| C3 | Santa Cruz Biotechnology | Cat# sc-8399 |
| CD3 | eBioscience | Cat# 85-17-0032-82 |
| CD45 | eBioscience | Cat# 69-0451-82 |
| B220 | eBioscience | Cat# 11-0452-85 |
| CD45R/B220 (RA3-6B2) | Biolegend | Cat# 103236 |
| IgG | Proteintech | Cat# 66360-3-Ig |
| Nr4a1 | Santa Cruz Biotechnology | Cat# sc-365113 |
| Nr4a2 | Santa Cruz Biotechnology | Cat# sc-376984 |
| Nr4a3 | Santa Cruz Biotechnology | Cat# sc-393902 |
| C5b-9 | Santa Cruz Biotechnology | Cat# sc-66190 |
| horseradish peroxidase-conjugated goat anti-mouse IgG secondary antibody | Thermo Fisher Scientific | Cat# 31430 |
| horseradish peroxidase-conjugated goat rabbit IgG secondary antibody | Thermo Fisher Scientific | Cat# 31460 |
| Alexa Fluor 555 goat anti-mouse IgG | Invitrogen | Cat# A 21422 |
| Alexa Fluor 555 goat anti-rabbit IgG | Invitrogen | Cat# A21429 |
| Alexa Fluor 555 donkey anti-goat IgG | Invitrogen | Cat# A21432 |
| Alexa Fluor 488 goat anti-rabbit IgG | Invitrogen | Cat# A11008 |
| Alexa Fluor 488 donkey anti-mouse IgG | Invitrogen | Cat# A21202 |
| **Chemicals, peptides, and recombinant proteins** |  |  |
| Phallacidin | Abcam | Cat# ab143532 |
| Purified mouse C1q | Complement Tech | Cat# M099 |
| Purified human C1q | Complement Tech | Cat# A099 |
| ProLong Gold Antifade Mountant | Invitrogen | Cat# P36930 |
| Minocycline | Sigma-Aldrich | Cat# M2280000 |
| Neurobasal medium | Invitrogen, GIBCO | Cat# 21103-049 |
| B27 | Invitrogen | Cat# 17504044 |
| PL | Sigma-Aldrich | Cat# P0899 |
| Protein A/G PLUS-Agarose | Santa Cruz Biotechnology | Cat# sc-2003 |
| NMDAR antagonist AP5 | Sigma-Aldrich | Cat# 165304 |
| Nr4a1 antagonist DIM-C-pPhCO2Me | Sigma-Aldrich | Cat# SML1976 |
| **Experimental models:** |  |  |
| Mouse: MRL/MpJ-Fas^lpr^ | Slac Laboratory Animal Center | The Jackson Laboratory: stock #006825 |
| Mouse: MRL/MpJ | Slac Laboratory Animal Center | The Jackson Laboratory: stock # 000486 |
| Mouse: C57BL/6J | Model Animal Research Center of Nanjing University | N/A |
| **Oligonucleotides** |  |  |
| LV: CON-RNAi | Hanbio |  |
| LV: Nr4a1-RNAi | Hanbio |  |
| **Recombinant DNA** |  |  |
| LV: ZsGreen-PURO-CON | Hanbio |  |
| LV-m-Nr4a1-3xflag-ZsGreen-PURO | Hanbio |  |
| **Software and algorithms** |  |  |
| Prism 6 Software | GraphPad | https://www.graphpad.com/scientific-software/prism/ |
| ImageJ Software | NIH | https://imagej.nih.gov/ij/ |
| Imaris 8.3.1 | Bitplane | http://www.bitplane.com/imaris |
| TopScan Software | CleverSys | http://cleversysinc.com/products/software/topscan/ |
| **Other** |  |  |
| FD Rapid GolgiStain Kit | FD Neurotechnologies | Cat# PK401 |
| Proteinase and Phosphatase Inhibitor Mini Tablets | Invitrogen | Cat# A32959 |
| TUNEL | Keygen | Cat# KGA7062 |
| Bradford Protein Detection Kit | Keygen | Cat# KGA801-804 |

**Table. S4. Antibodies for immunoblotting and for immunohistochemistry or immunofluorescence**

| **Antibodies used for immunoblotting studies** | | | |
| --- | --- | --- | --- |
| Antibody | Cat. | Company | Dilution |
| C1q | ab71940 | Abcam | 1:1000 |
| C1q | sc-53544 | Santa Cruz Biotechnology | 1:500 |
| C1q | sc-58920 | Santa Cruz Biotechnology | 1:500 |
| C5b-9 | sc-66190 | Santa Cruz Biotechnology | 1:500 |
| CD68 | #66231-2-Ig | Proteintech | 1:1000 |
| IBA-1 | #019-19741 | Wako | 1:1000 |
| MAP2 | #17490-1-AP | Proteintech | 1:1000 |
| Synaptophysin | #17785-1-AP | Proteintech | 1:1000 |
| NR4A1 | sc-365113 | Santa Cruz Biotechnology | 1:1000 |
| NR4A2 | sc-376984 | Santa Cruz Biotechnology | 1:1000 |
| NR4A3 | sc-393902 | Santa Cruz Biotechnology | 1:1000 |
| ACTB | #4970 | Cell Signaling Technology | 1:1000 |
| **Antibodies used for co-IP studies** | | | |
| C1q | #11602-1-AP | Proteintech | 1:250 |
| IgG | #66360-3-Ig | Proteintech | 1:250 |
| PSD-95 | #20665-1-AP | Proteintech | 1:500 |
| Synaptophysin | #17785-1-AP | Proteintech | 1:500 |
| **Antibodies used for immunohistochemistry or immunofluorescence** | | | |
| C1q | ab71940 | Abcam | 1:500 |
| C1q | #11602-1-AP | Proteintech | 1:250 |
| CD68 | AB_322219 | Bio-Rad | 1:500 |
| LAMP1(clone 1D4B) | Santa Cruz Biotechnology | Santa Cruz Biotechnology | 1:500 |
| IBA-1 | #019-19741 | Wako | 1:500 |
| TMEM119 | ab209064 | Abcam | 1:500 |
| MAP2 | ab11267 | Abcam | 1:500 |
| NeuN | ab177487 | Abcam | 1:500 |
| PSD-95 | ab2723 | Millipore | 1:500 |
| Synaptophysin | N101002 | Synaptic Systems | 1:500 |
| VGLUT1 | N1602-At488-S | Synaptic Systems | 1:500 |
| VGAT-1 | AB1570W | Millipore | 1:500 |
| Gephyrin (clone G-6) | sc-25311 | Santa Cruz Biotechnology | 1:500 |
| C3 | sc-8399 | Santa Cruz Biotechnology | 1:500 |
| CD3 | #85-17-0032-82 | eBioscience | 1:500 |
| CD45 | #69-0451-82 | eBioscience | 1:500 |
| B220 | #11-0452-85 | eBioscience | 1:500 |
| CD45R/B220 (RA3-6B2) | #103236 | Biolegend | 1:500 |
| Alexa Fluor 555 goat anti-mouse IgG | # A 21422 | Invitrogen | 1:500 |
| Alexa Fluor 555 goat anti-rabbit IgG | # A21429 | Invitrogen | 1:500 |
| Alexa Fluor 555 donkey anti-goat IgG | # A21432 | Invitrogen | 1:500 |
| Alexa Fluor 488 goat anti-rabbit IgG | # A11008 | Invitrogen | 1:500 |
| Alexa Fluor 488 donkey anti-mouse IgG | # A21202 | Invitrogen | 1:500 |
