# Supplementary material for: Neuronal NR4A1 deficiency drives complement-coordinated synaptic stripping by microglia in a mouse model of lupus
Source: Signal Transduct Target Ther. 2022 Feb 18;7:50. doi: 10.1038/s41392-021-00867-y (PMC8854434; doi:10.1038/s41392-021-00867-y)

Figure 1l, 3a, 3c, 3g, 4b, 4c, 5a, 5b, 6e, 6g, 7d, S5a-e, S6g, S6k, S6l, S9c.

Figure 1 l

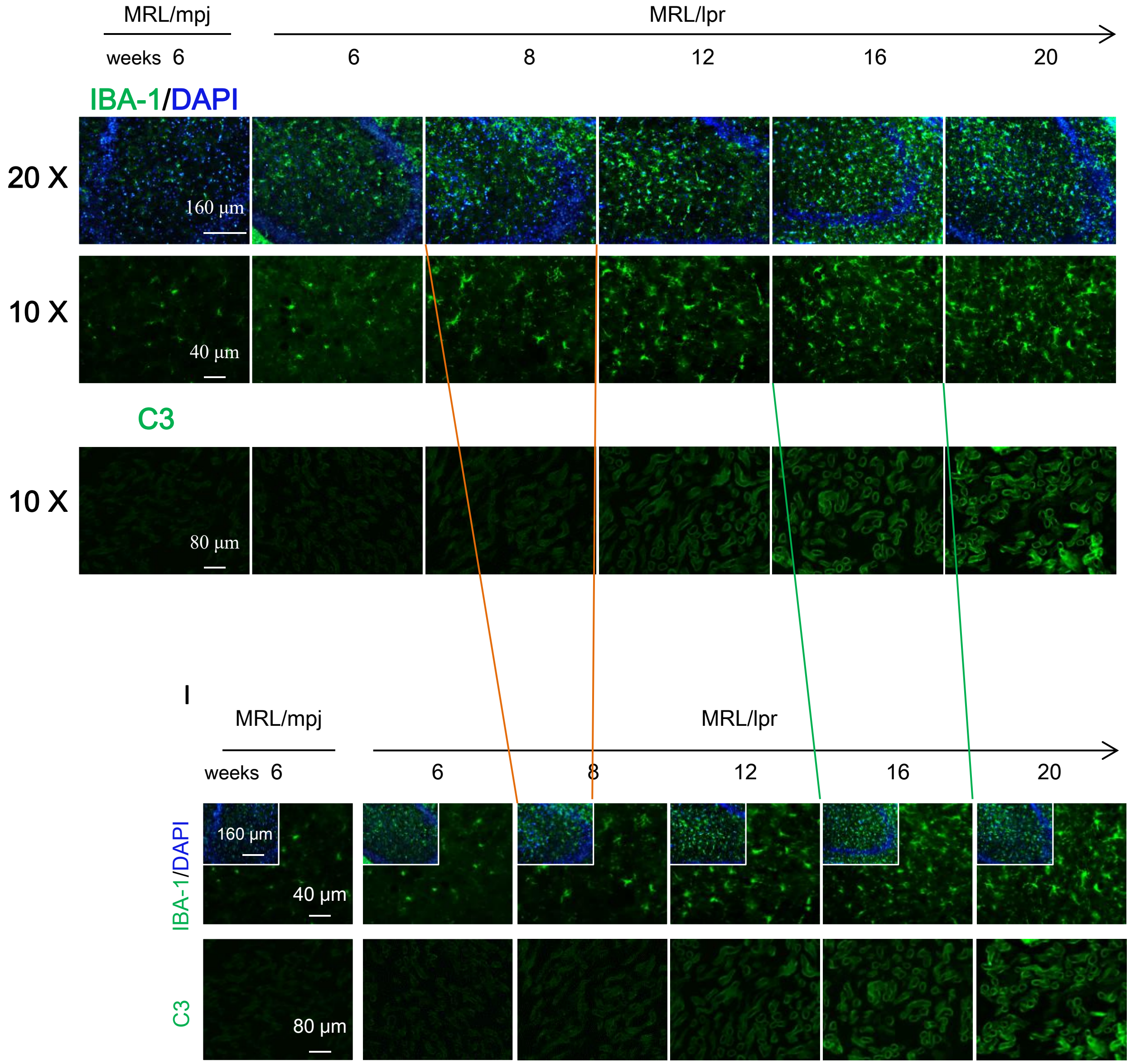

Figure 1l, 3a, 3c, 3g, 4b, 4c, 5a, 5b, 6e, 6g, 7d, S5a-e, S6g, S6k, S6l, S9c.

Figure 3a

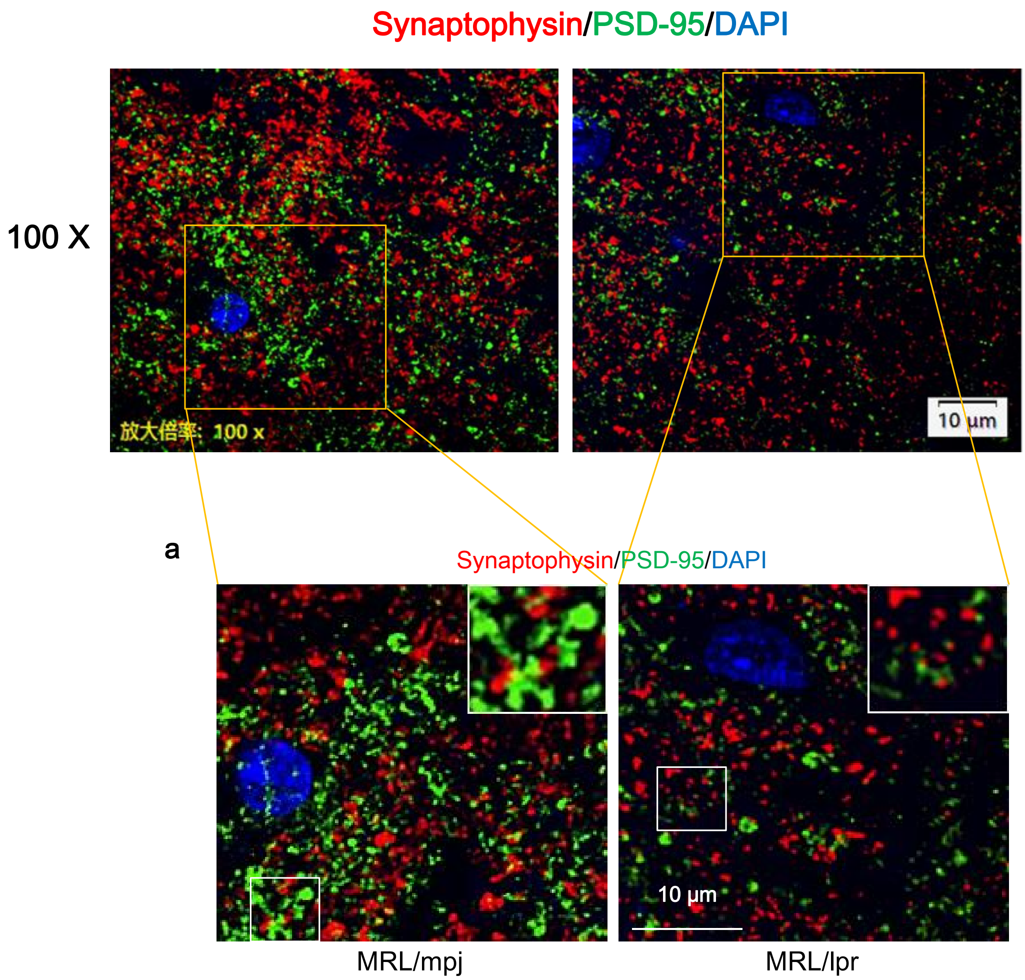

Figure 1l, 3a, 3c, 3g, 4b, 4c, 5a, 5b, 6e, 6g, 7d, S5a-e, S6g, S6k, S6l, S9c.

Figure 3c

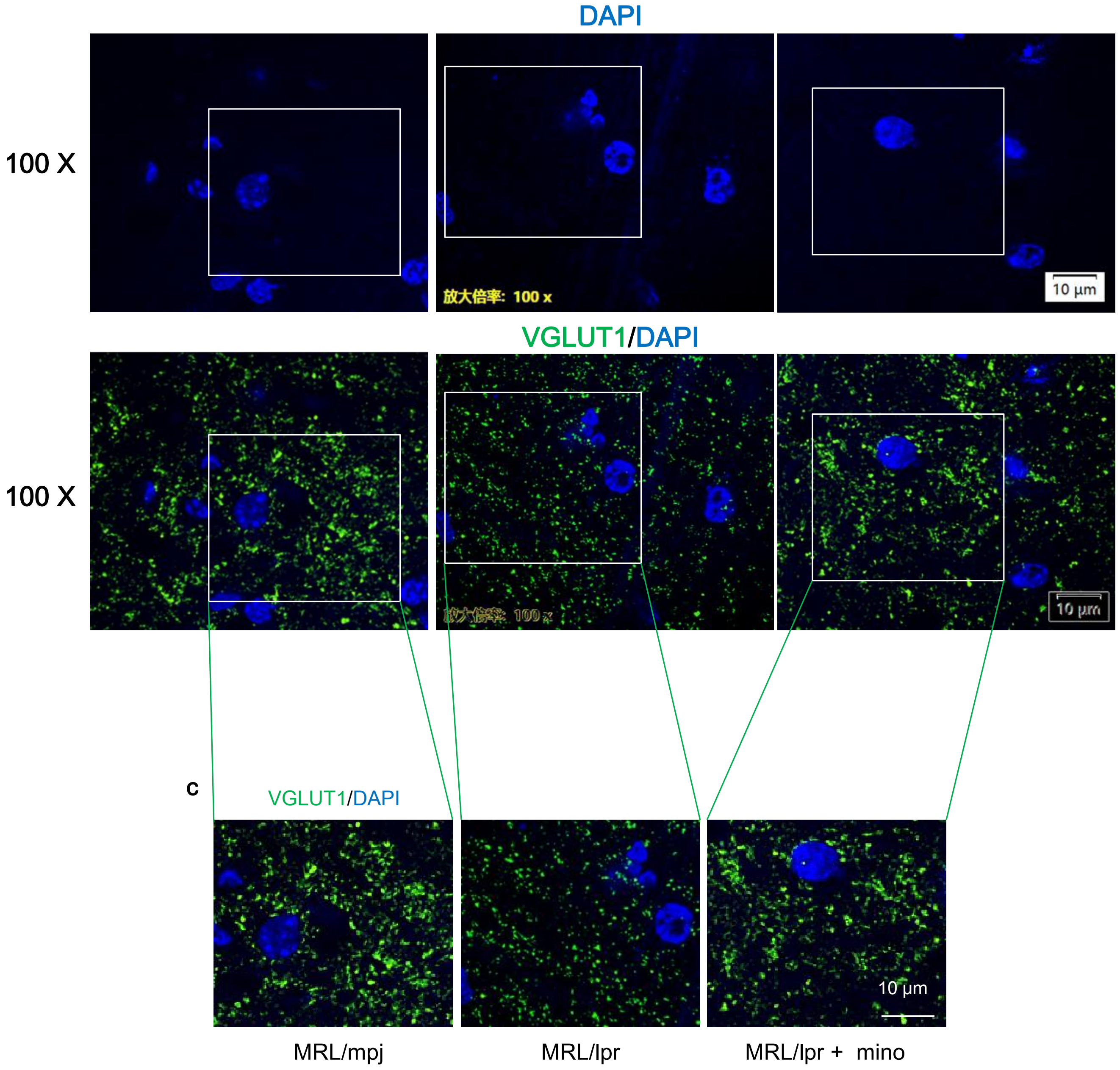

Figure **1l, 3a, 3c, 3g,** 4b, 4c, 5a, 5b, 6e, 6g, 7d, S5a-e, S6g, S6k, S6l, S9c.

Figure 3g

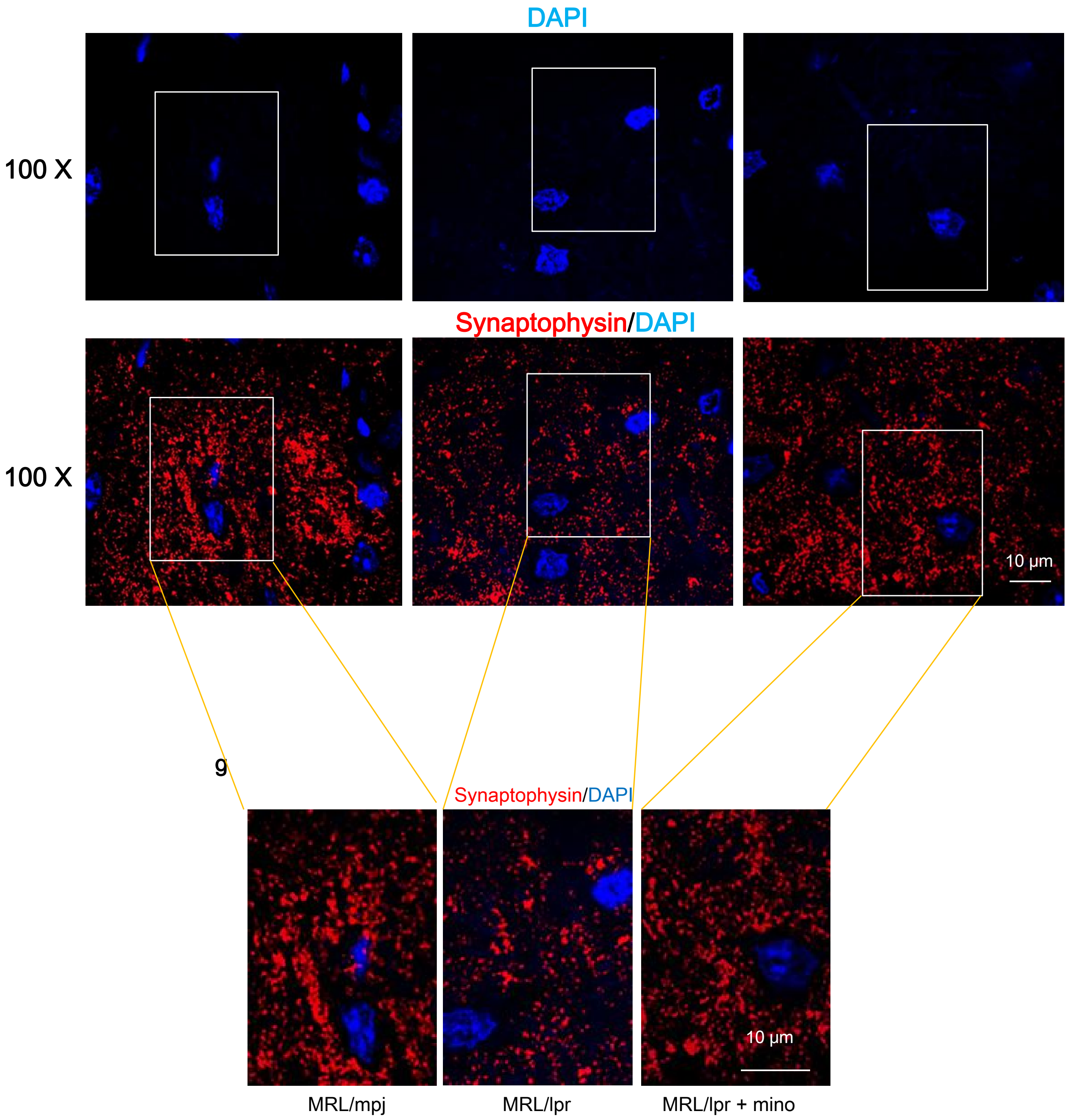

Figure 1l, 3a, 3c, 3g, 4b, 4c, 5a, 5b, 6e, 6g, 7d, S5a-e, S6g, S6k, S6l, S9c.

Figure 4b

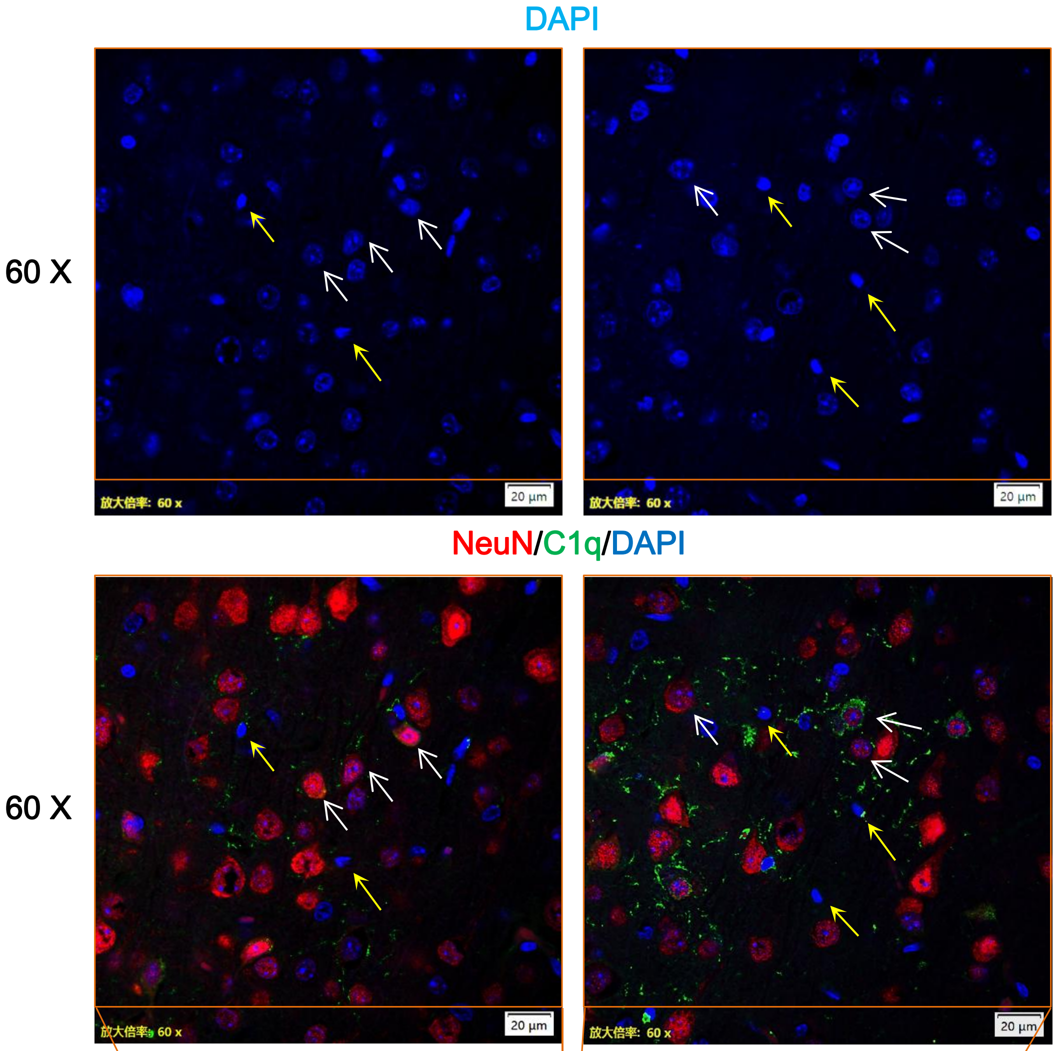

白色箭头-核大、圆形多为神经元（NeuN阳性）
黄色箭头-核小多为胶质细胞

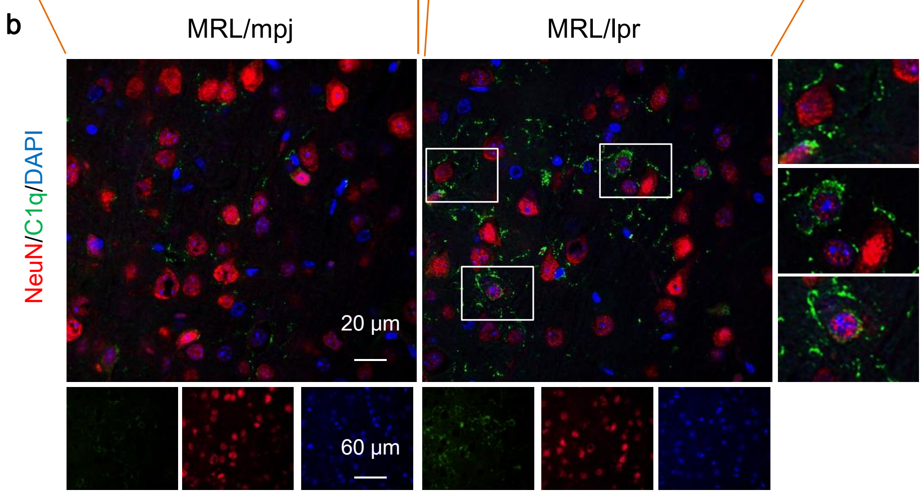

Figure 1l, 3a, 3c, 3g, 4b, 4c, 5a, 5b, 6e, 6g, 7d, S5a-e, S6g, S6k, S6l, S9c.

Figure 4c

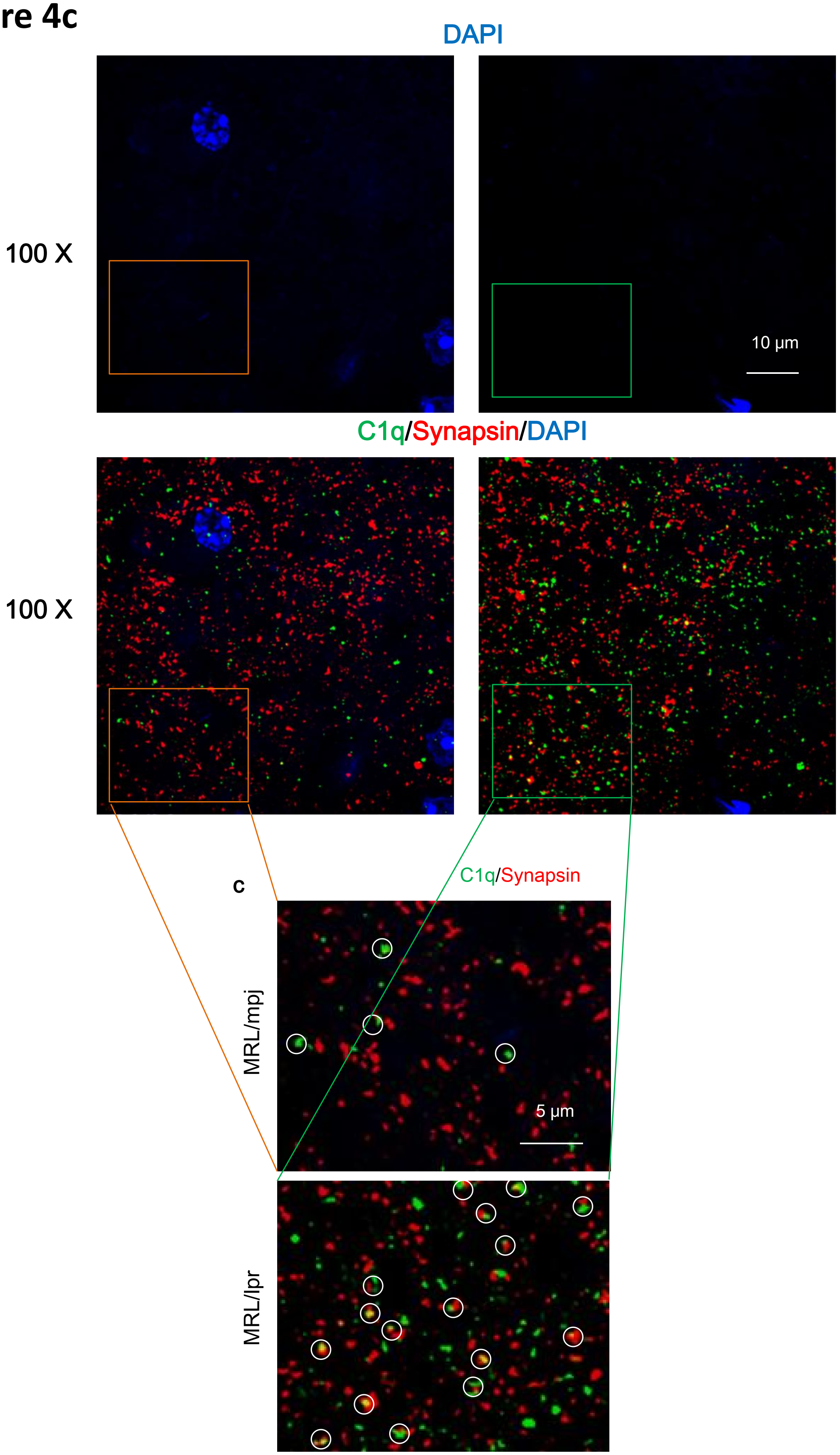

Figure 1l, 3a, 3c, 3g, 4b, 4c, 5a, 5b, 6e, 6g, 7d, S5a-e, S6g, S6k, S6l, S9c.

Figure 5a

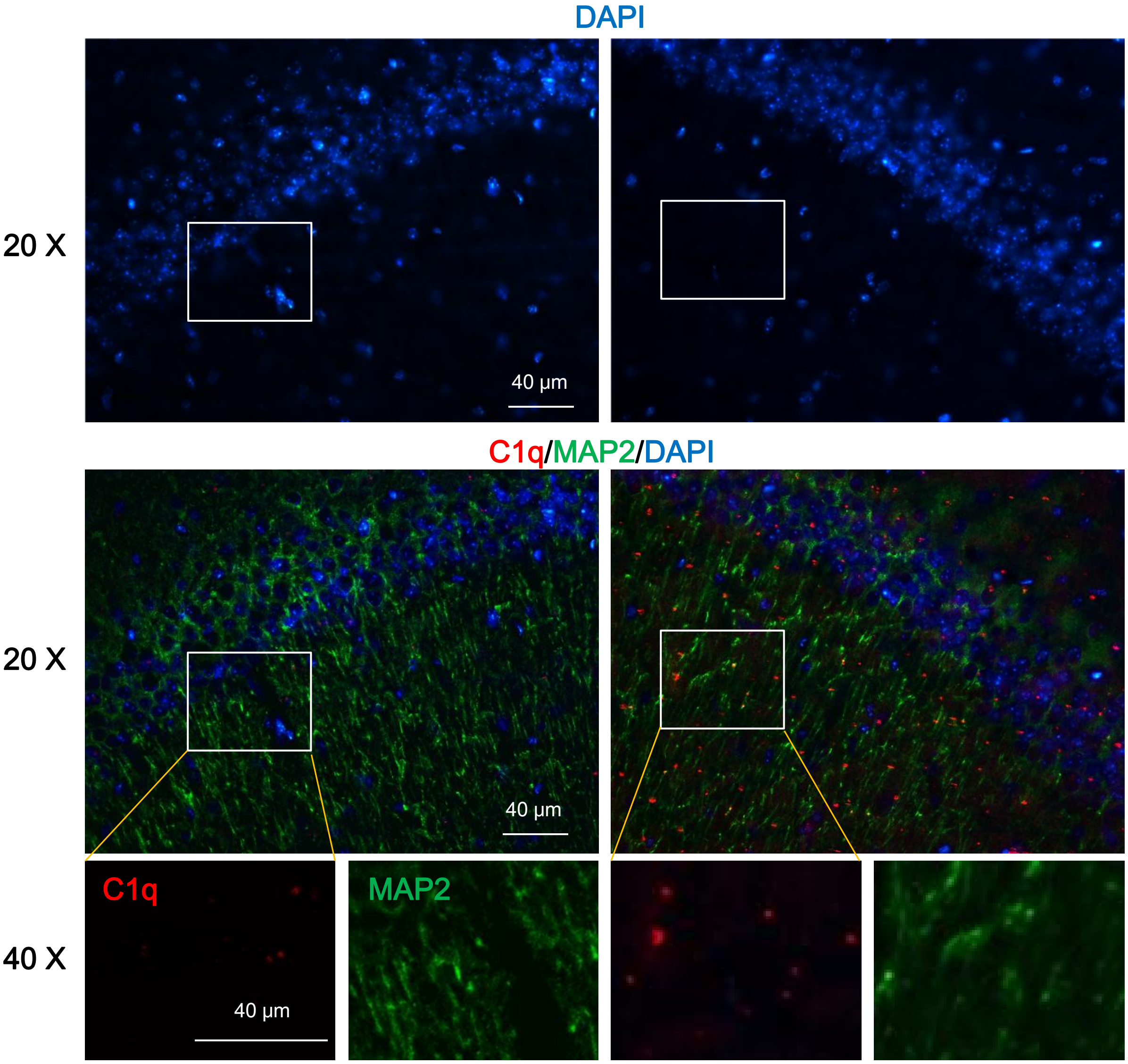

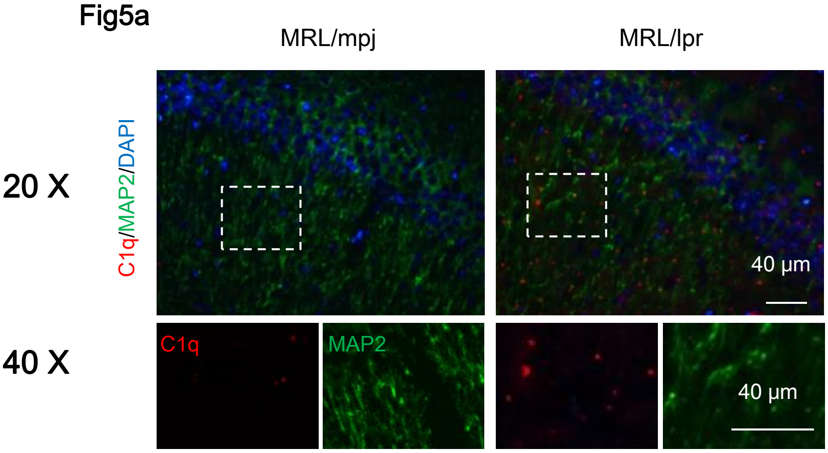

Figure 1l, 3a, 3c, 3g, 4b, 4c, 5a, 5b, 6e, 6g, 7d, S5a-e, S6g, S6k, S6l, S9c.

Figure 5b

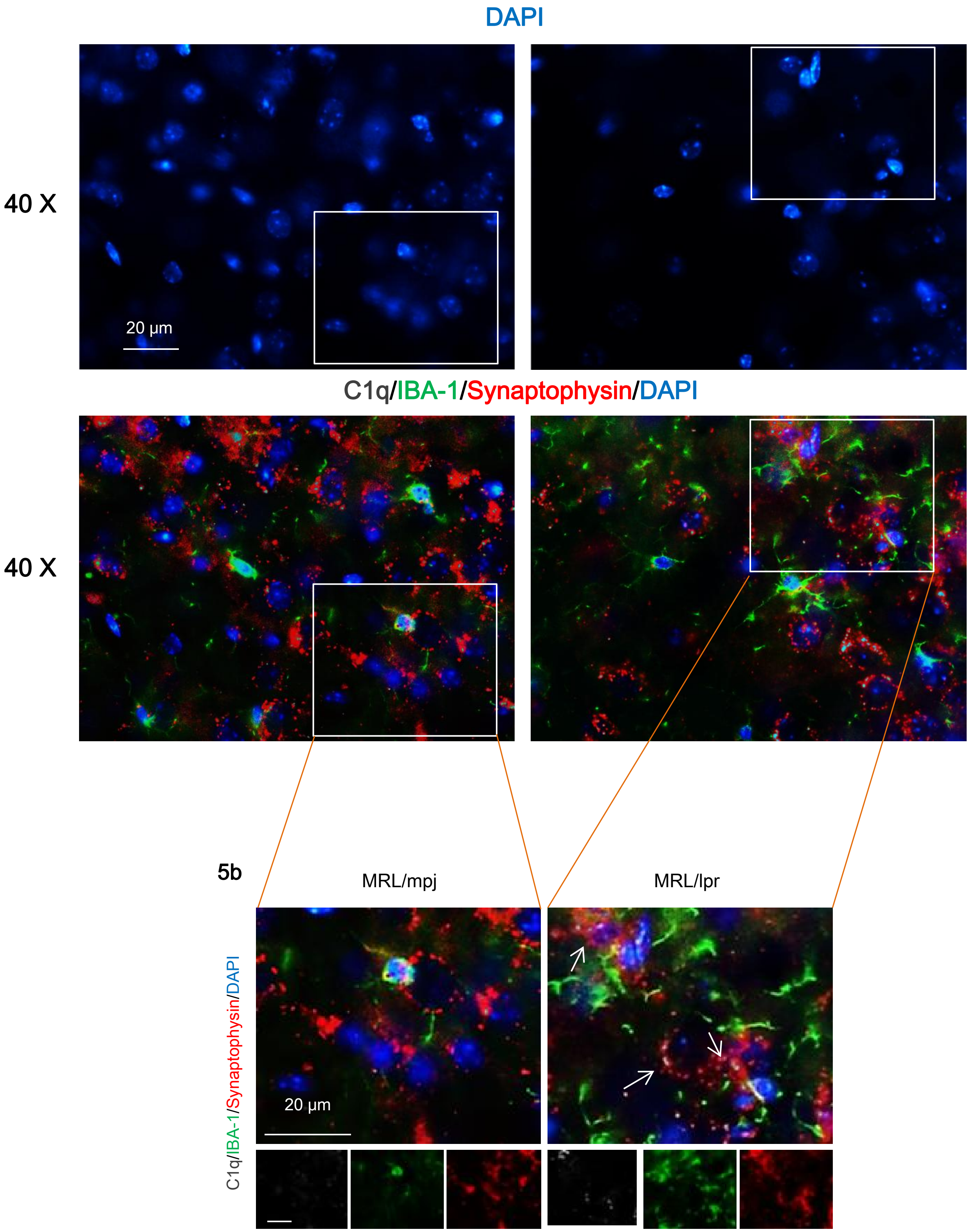

Figure 1l, 3a, 3c, 3g, 4b, 4c, 5a, 5b, 6e, 6g, 7d, S5a-e, S6g, S6k, S6l, S9c.

Figure 6e

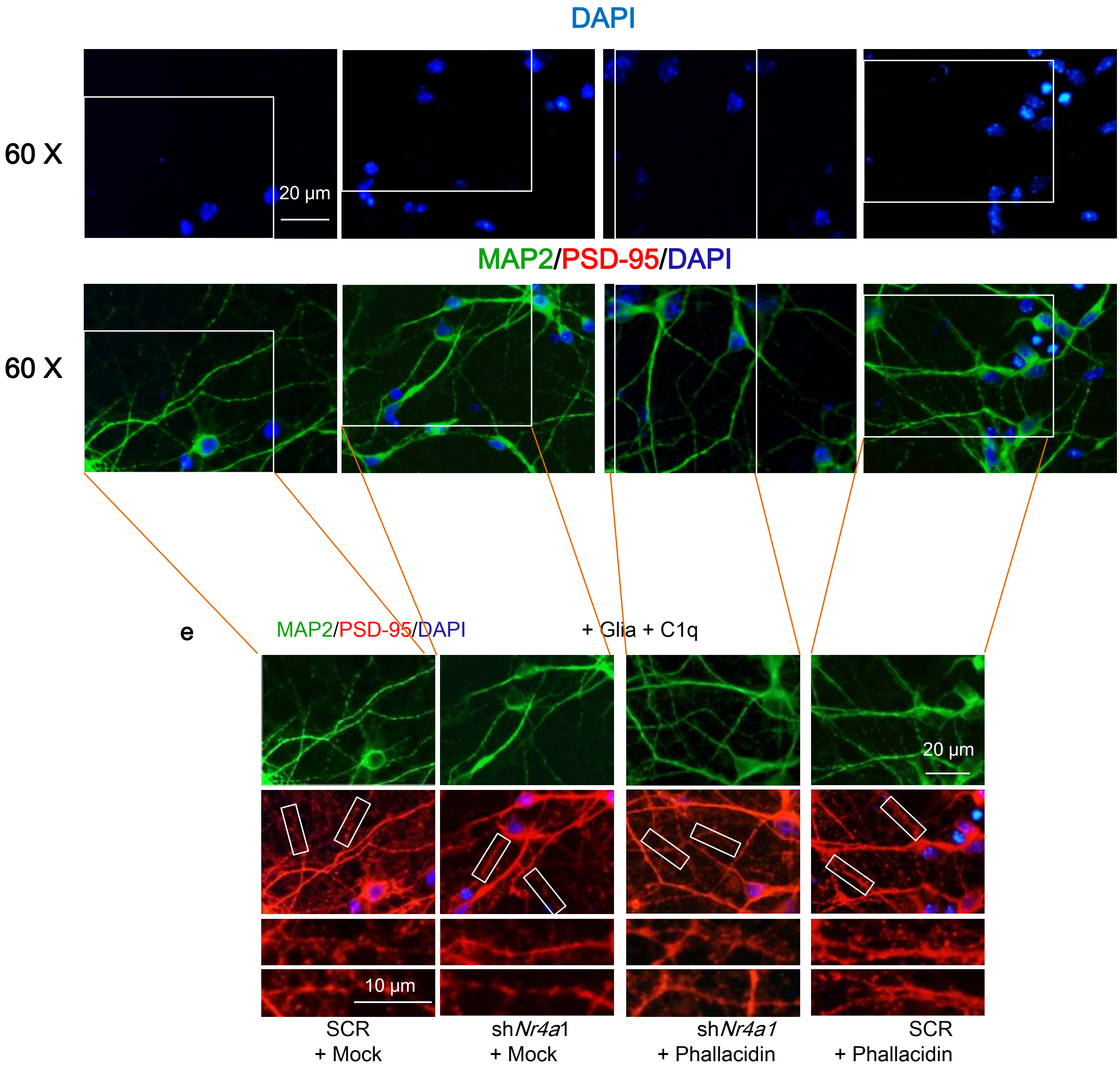

Figure 1l, 3a, 3c, 3g, 4b, 4c, 5a, 5b, 6e, 6g, 7d, S5a-e, S6g, S6k, S6l, S9c.

Figure 6g

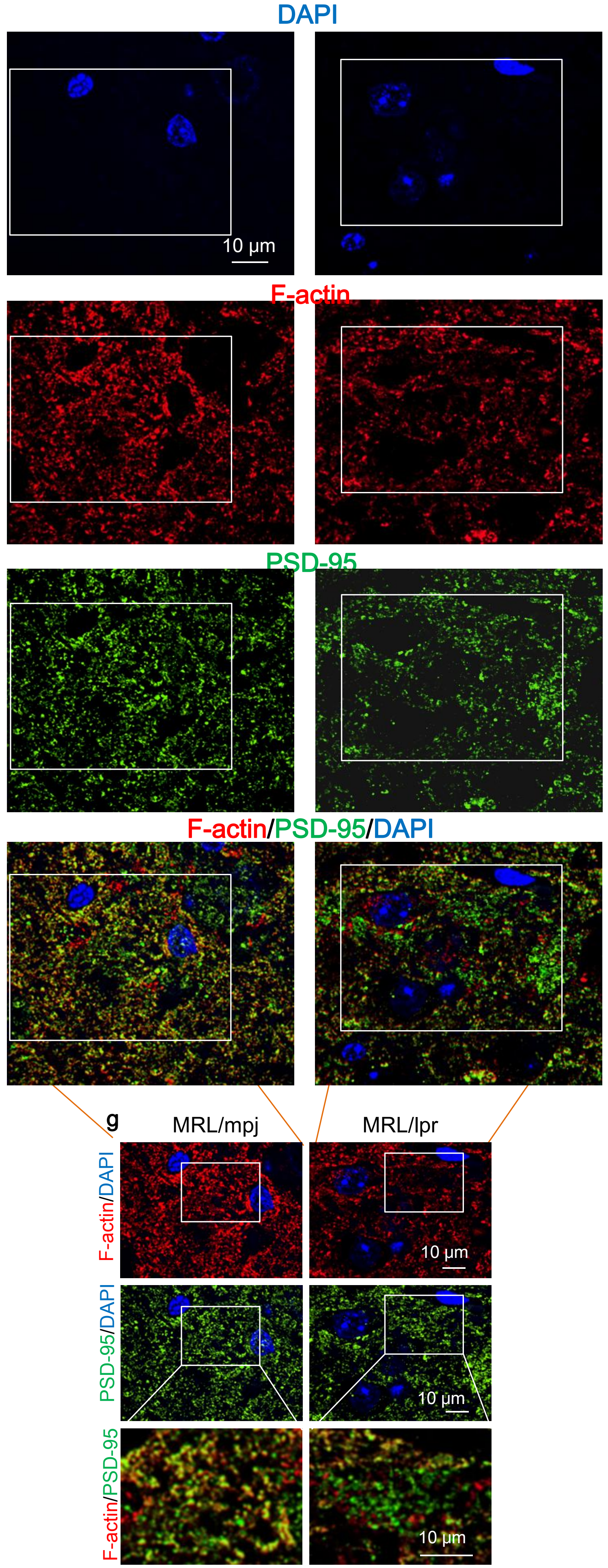

Figure 1l, 3a, 3c, 3g, 4b, 4c, 5a, 5b, 6e, 6g, 7d, S5a-e, S6g, S6k, S6l, S9c.

Figure 7d

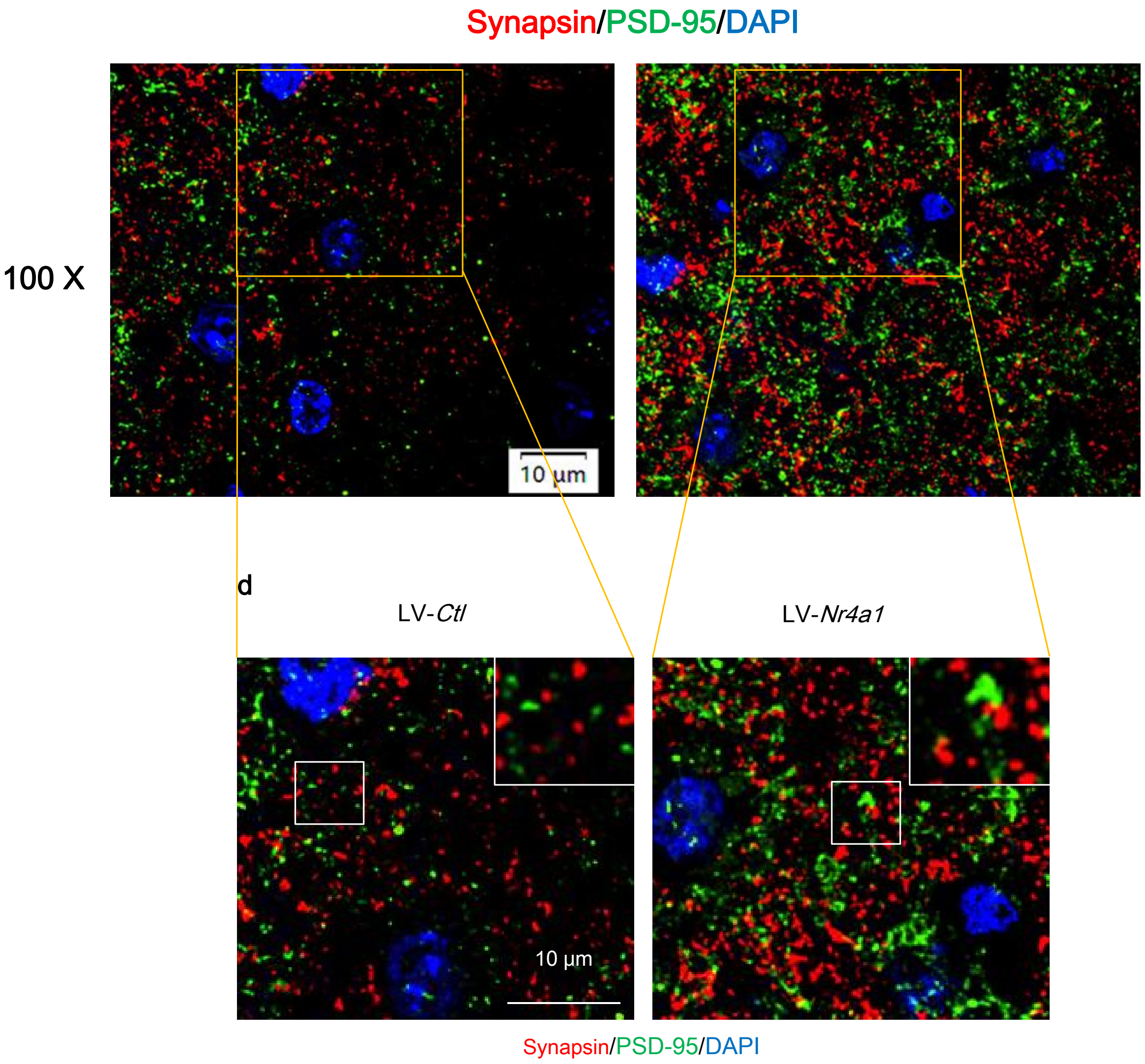

Figure 1l, 3a, 3c, 3g, 4b, 4c, 5a, 5b, 6e, 6g, 7d, S5a-e, S6g, S6k, S6l, S9c.

Figure S5a

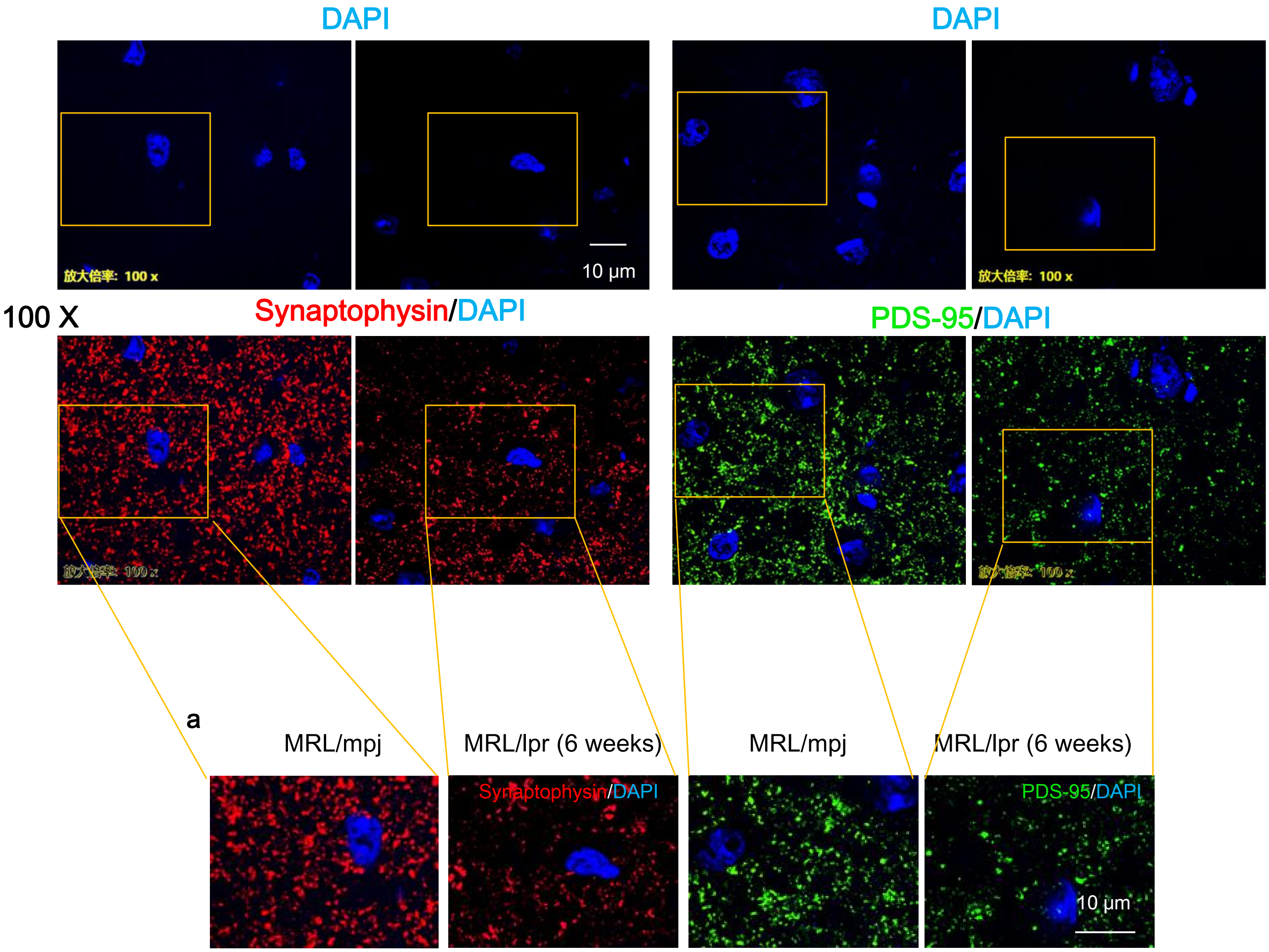

Figure 1l, 3a, 3c, 3g, 4b, 4c, 5a, 5b, 6e, 6g, 7d, S5a-e, S6g, S6k, S6l, S9c.

Figure S5b

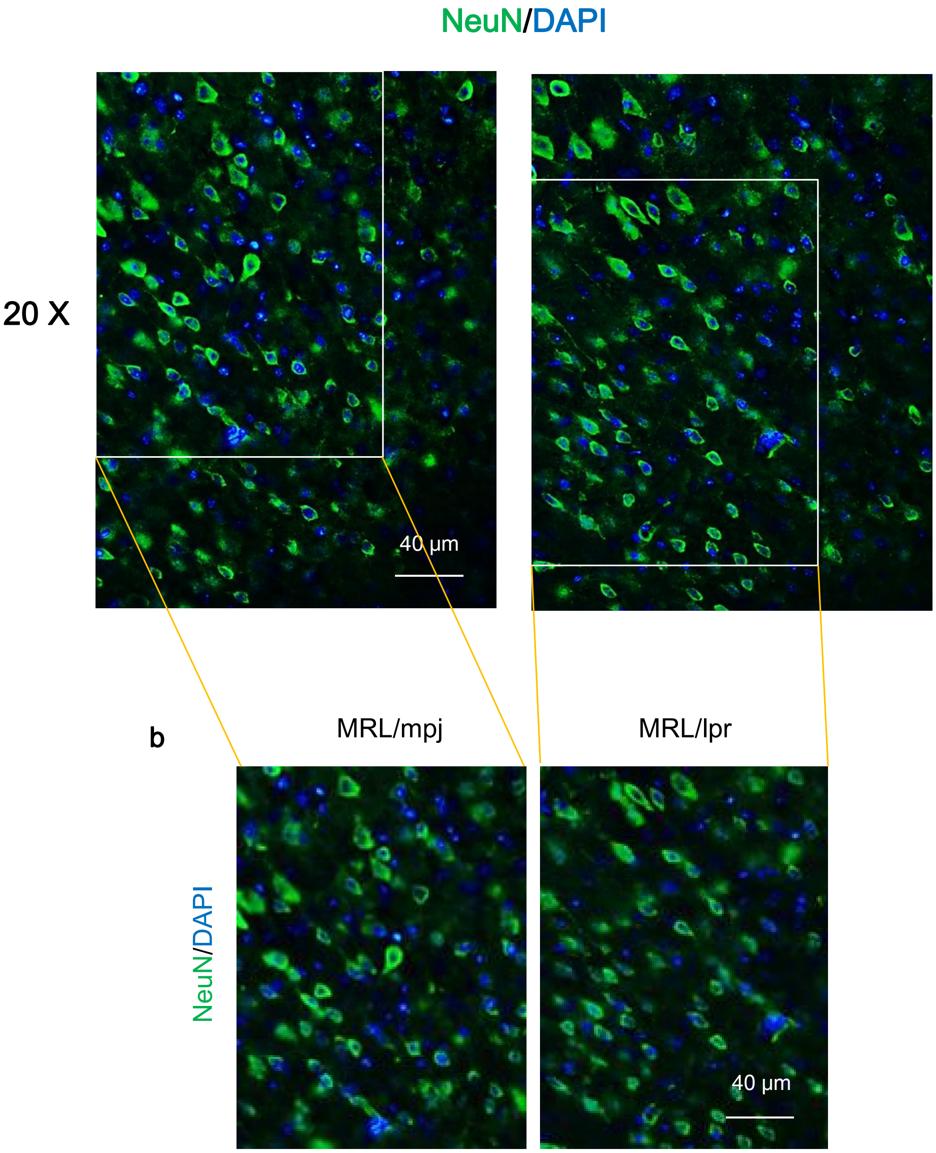

Figure 1l, 3a, 3c, 3g, 4b, 4c, 5a, 5b, 6e, 6g, 7d, S5a-e, S6g, S6k, S6l, S9c.

Figure S5c

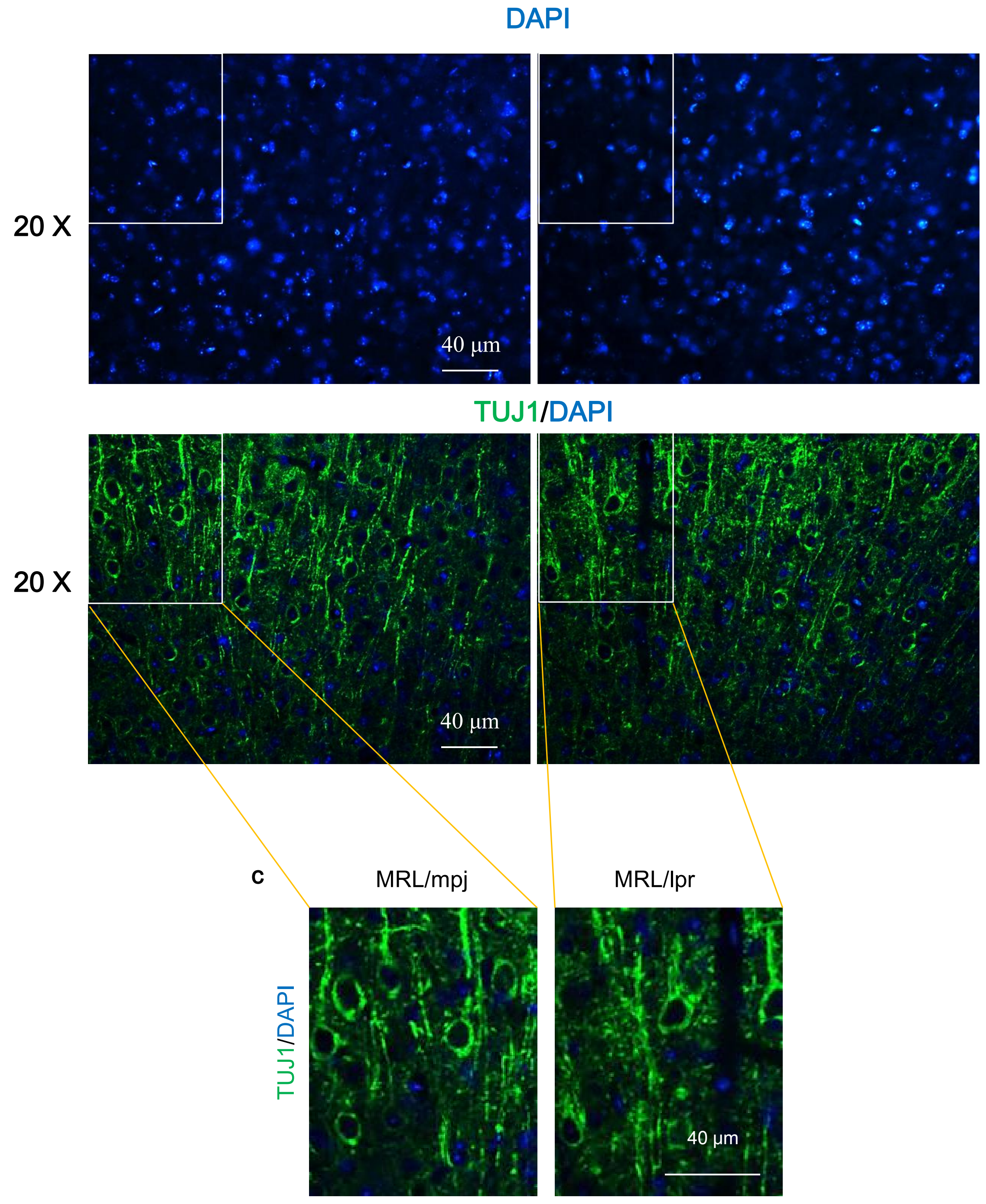

Figure 1l, 3a, 3c, 3g, 4b, 4c, 5a, 5b, 6e, 6g, 7d, S5a-e, S6g, S6k, S6l, S9c.

Figure S5d

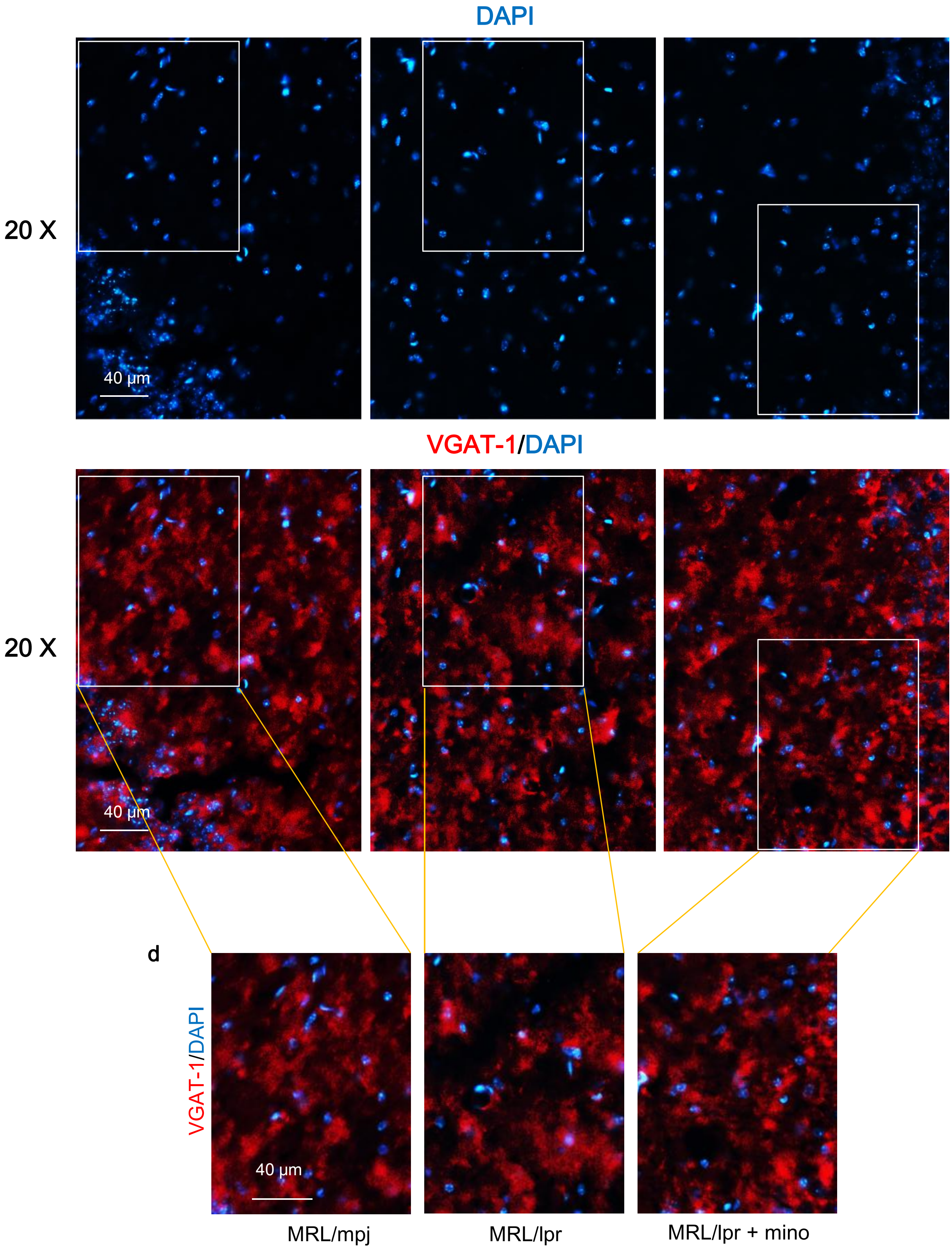

Figure 1l, 3a, 3c, 3g, 4b, 4c, 5a, 5b, 6e, 6g, 7d, S5a-e, S6g, S6k, S6l, S9c.

Figure S5e

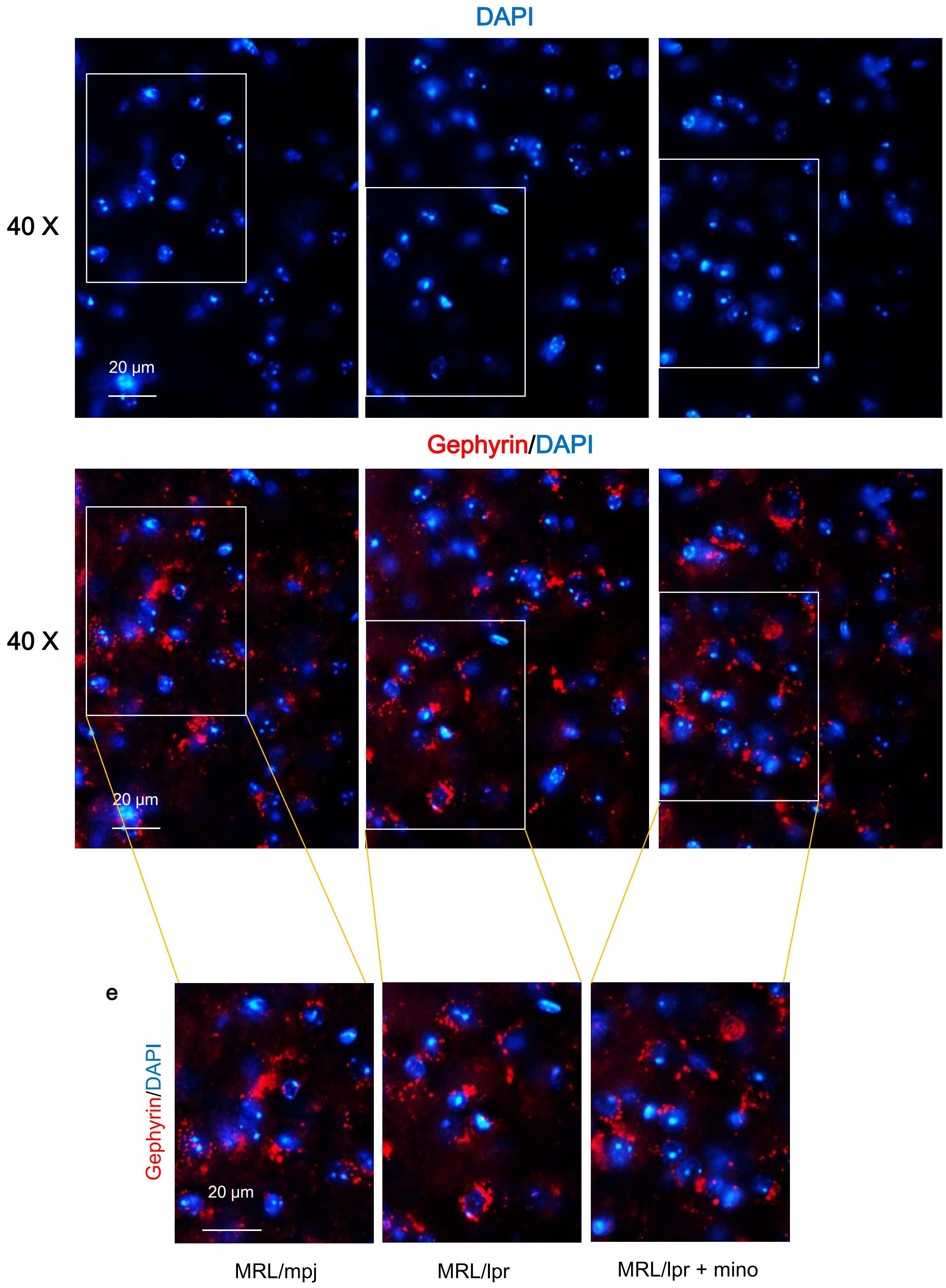

Figure 1l, 3a, 3c, 3g, 4b, 4c, 5a, 5b, 6e, 6g, 7d, S5a-e, S6g, S6k, S6l, S9c.

Figure S6g

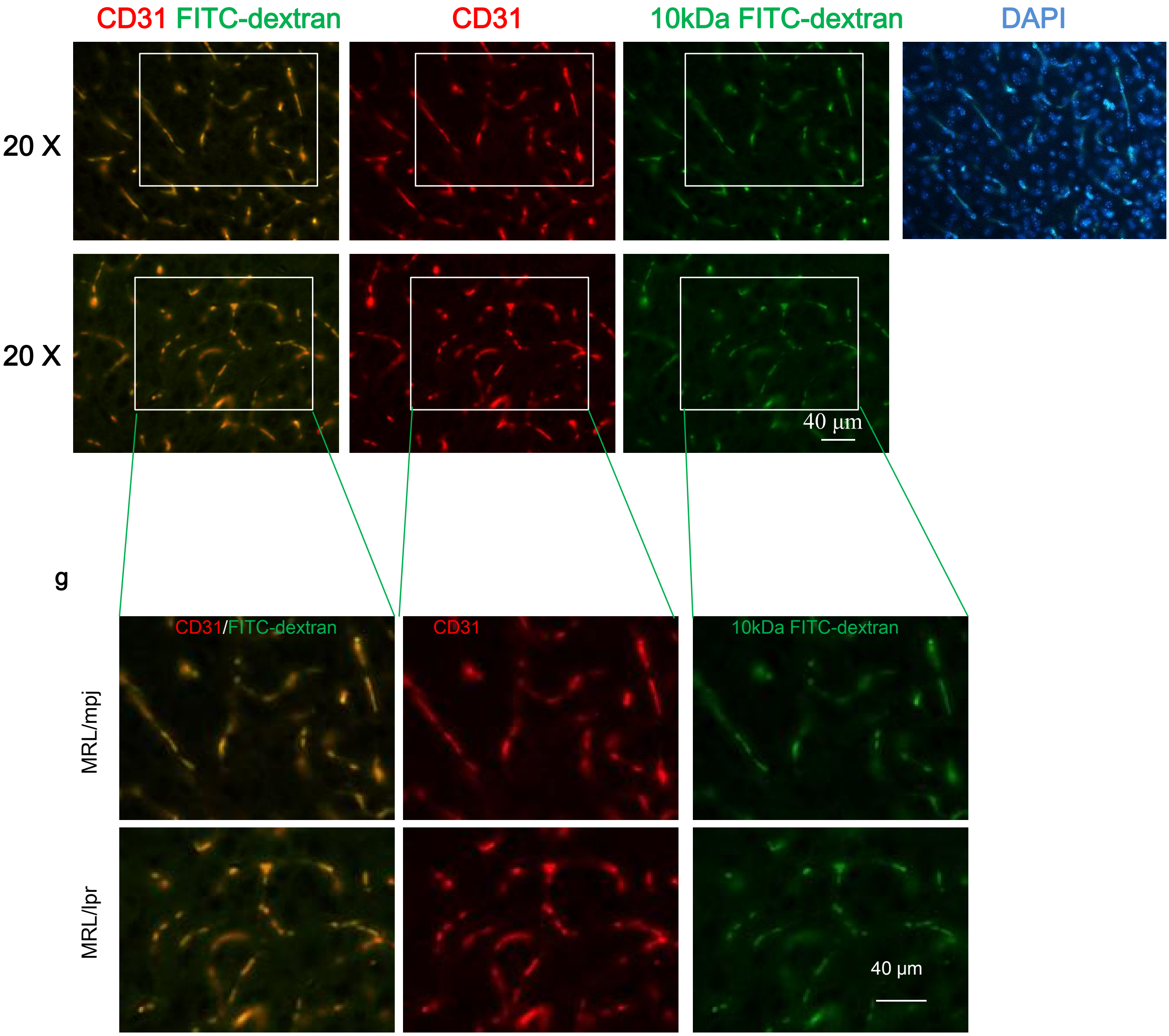

Figure 1l, 3a, 3c, 3g, 4b, 4c, 5a, 5b, 6e, 6g, 7d, S5a-e, S6g, S6k, S6l, S9c.

Figure S6k

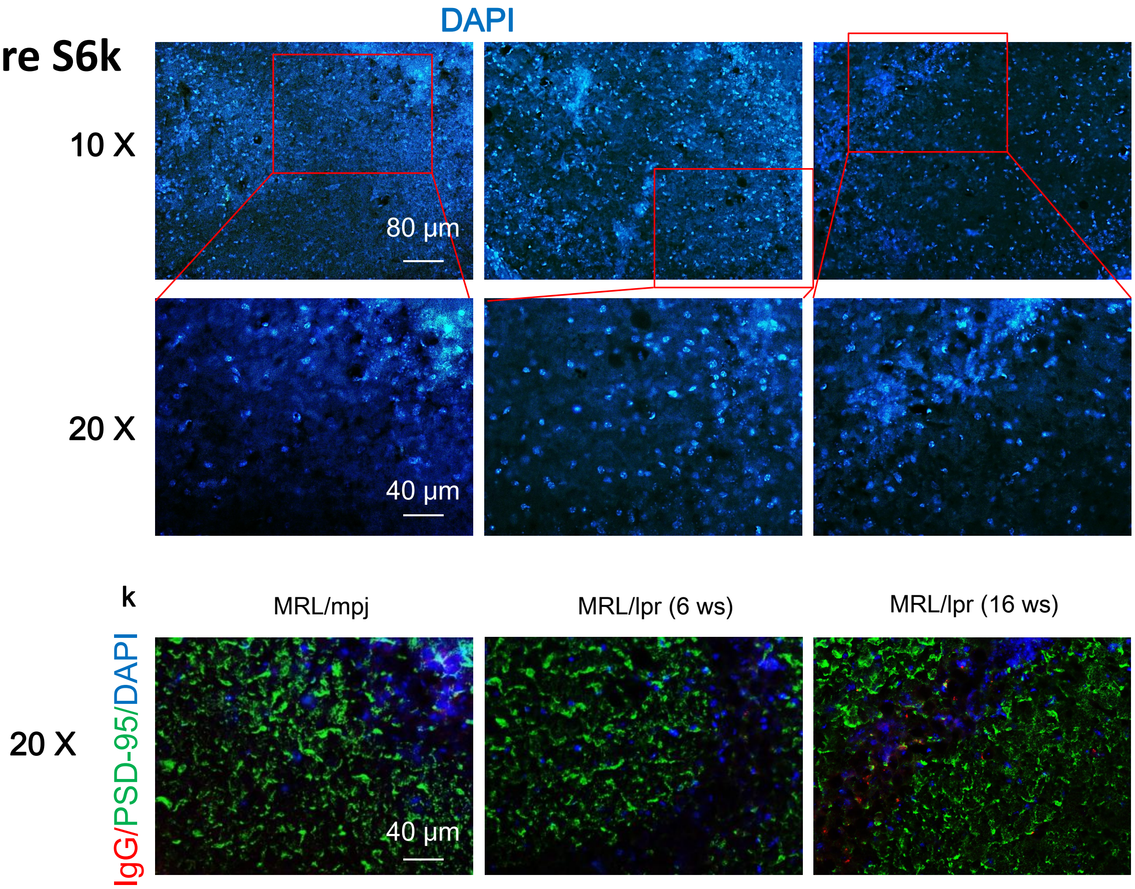

Figure S6l

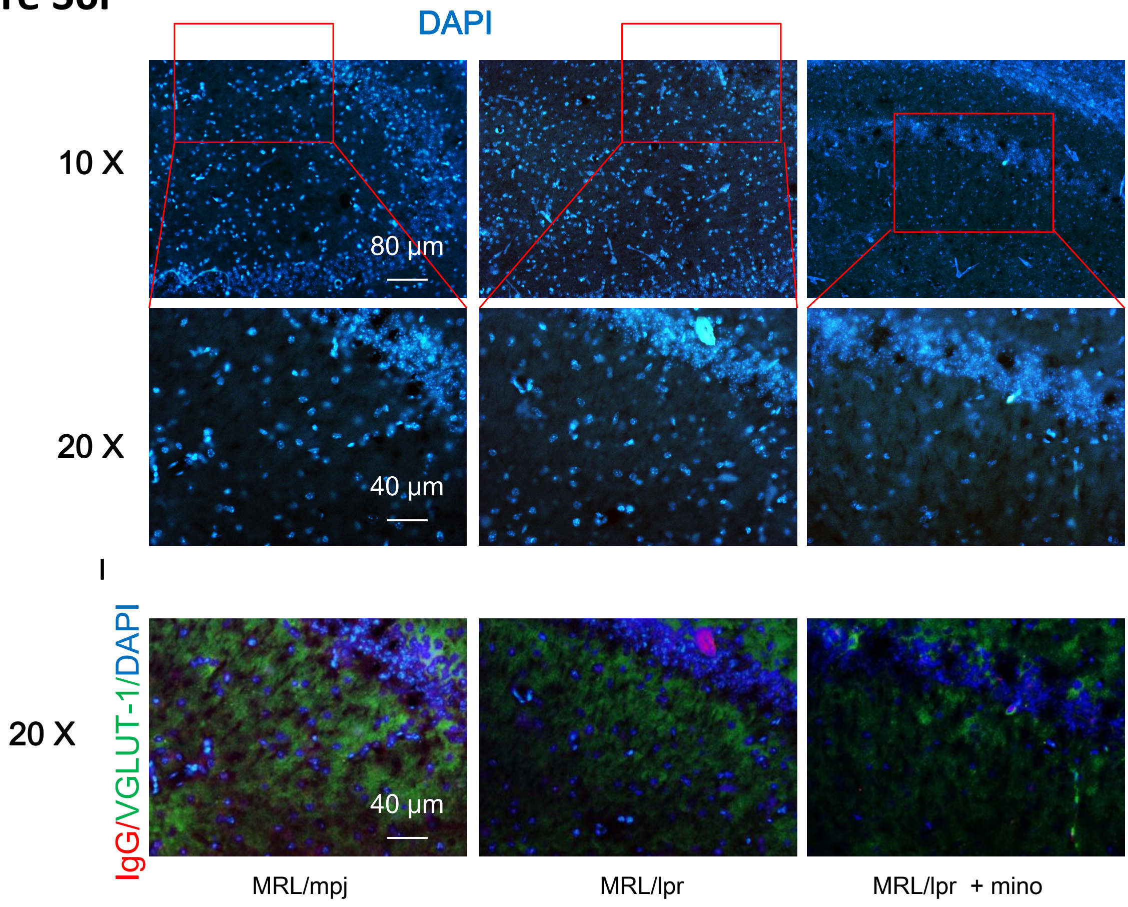

Figure 1l, 3a, 3c, 3g, 4b, 4c, 5a, 5b, 6e, 6g, 7d, S5a-e, S6g, S6k, S6l, S9c.

Figure S9c

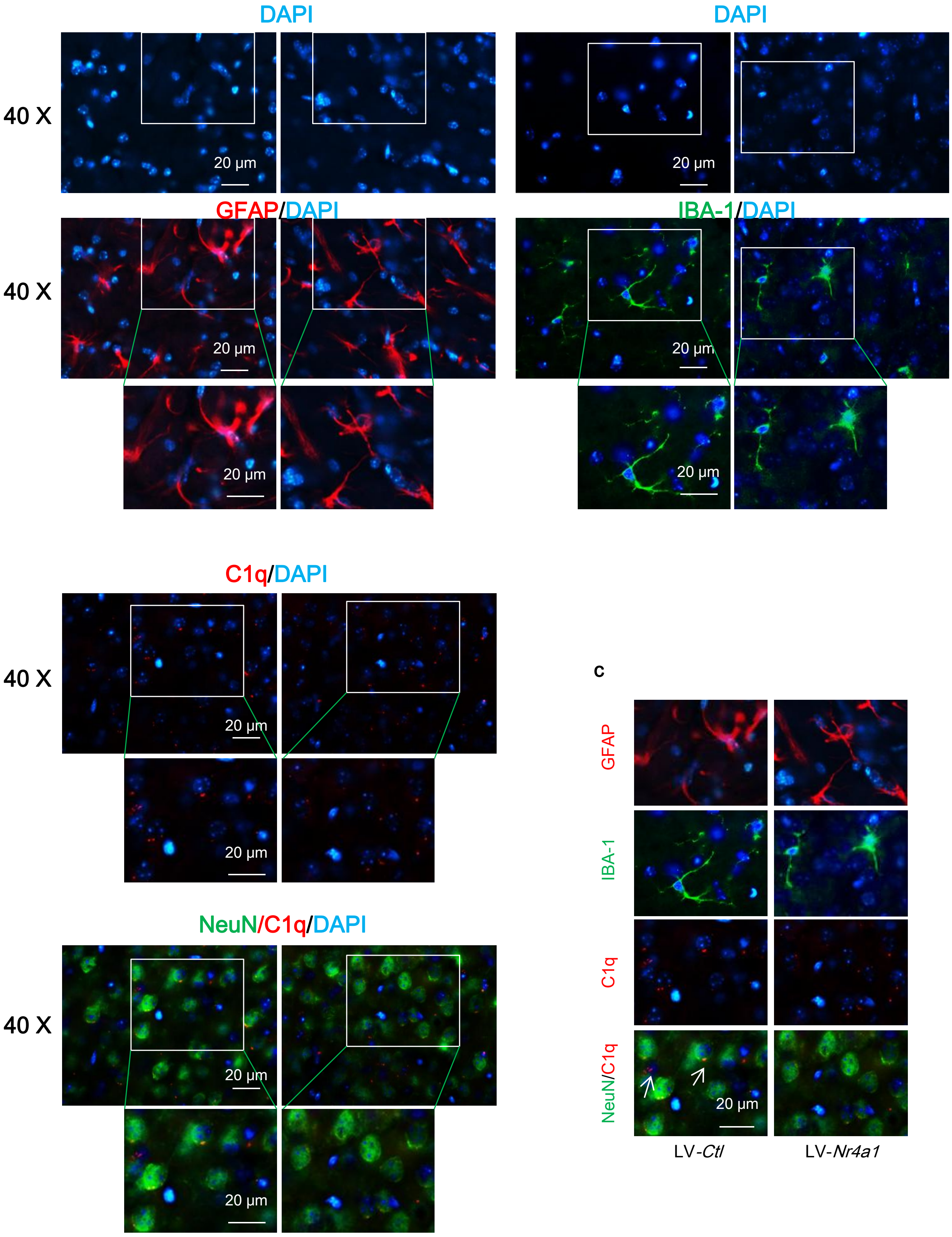

Supplement: Supplementary file 3 — Supplementary Data 2 [file 41392_2021_867_MOESM3_ESM.pdf]
